# Twist and Shine: The Impact of Halogen Substitution on Thiele Hydrocarbon's Optical Properties

**DOI:** 10.1002/anie.202524043

**Published:** 2025-12-17

**Authors:** Angela Punzi, Tobias Ullrich, Michele Orza, Davide Mesto, Anna Moliterni, Vincent Olieric, Sylvain Engilberge, Cinzia Giannini, Fabrizia Negri, Dirk M. Guldi, Davide Blasi, Gianluca M. Farinola

**Affiliations:** ^1^ Dipartimenti di Chimica Università degli Studi di Bari Aldo Moro, Via E. Orabona 4 Bari 70125 Italy; ^2^ Department of Chemistry and Pharmacy and Interdisciplinary Center for Molecular Materials (ICMM) Friedrich Alexander University Erlangen‐Nuremberg 91058 Erlangen Germany; ^3^ Dipartimento di Chimica “Giacomo Ciamician” Università di Bologna Via Piero Gobetti 85 Bologna 40129 Italy; ^4^ Center for Chemical Catalysis—C3 Università di Bologna Bologna 40129 Italy; ^5^ Istituto di Cristallografia CNR, Via Amendola, 122/O Bari 70126 Italy; ^6^ Swiss Light Source Paul Scherrer Institute Villigen PSI Forschungsstrasse 5232 Switzerland; ^7^ Université Grenoble Alpes, CNRS, CEA Institut de Biologie Structurale Grenoble France; ^8^ INSTM UdR Bologna Bologna 40129 Italy

**Keywords:** polyhalogenated trityl radicals, singlet diradicaloids, sudden polarization, Thiele hydrocarbon, zwitterionic excited states, mechanofluorochromism

## Abstract

In this work, two new mixed‐halide Thiele hydrocarbons were synthesized and characterized to elucidate the influence of halogenation patterns on their photophysical properties, addressing the role exerted by steric constraints and electronic effects. Interestingly, their interplay governed a unique spectroscopic behavior that is driven by pronounced geometric rearrangements upon photoexcitation. Quantum‐chemical calculations revealed that derivatives with limited diradical character, and correspondingly shorter exocyclic C═C bonds, are likely to adopt a folded boat‐like conformation in the ground state, a structural motif not previously observed in Thiele hydrocarbons. Upon photo‐excitation, these exocyclic bonds were subject to a significant elongation, facilitating mixing between the bright singly excited (SE) state and the dark, doubly excited (DE) state. These interactions enabled the formation of a zwitterionic excited state, a finding that is consistent with recent observations for halogenated derivatives. Transient absorption spectroscopy confirmed this mechanism, which provides a promising strategy for designing fluorophores with exceptionally large Stokes shifts (more than 2 eV) and tailored photophysical and electronic properties. In addition, a pronounced mechanofluorochromic response has been observed for the first time in the case of folded derivative, opening the way to the use of these species as multi‐stimuli‐responsive molecular materials.

## Graphical abstract

Halogenation in Thiele hydrocarbons strongly influences structure and excited‐state dynamics. Depending on substitution, “boat” folded forms show large Stokes shifts and strong solvatochromism, while planar “butterfly” derivatives exhibit near‐unity photoluminescence yields. Halogen substitution thus enables precise tuning of charge separation and responsiveness in functional materials.

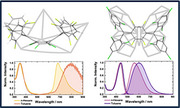

## Introduction

**Figure 1:**
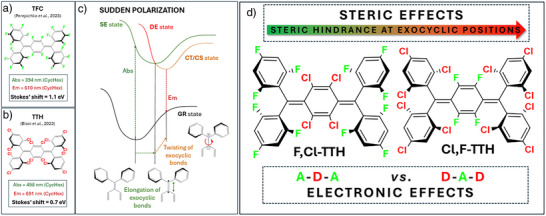
Molecular structures and main photophysical properties of a) **TFC** and b) **TTH** in cyclohexane. c) Pictorial representation of the sequential relaxation pathways with a sudden polarization that leads to the formation of charge‐transfer (CT) or charge‐separated (CS) states (by mixing of the SE and DE states) and that is responsible for the luminescence of PHTHs. d) Molecular structures of **F,Cl‐TTH** and **Cl,F‐TTH**, underlining the two main effects induced by changes in the halogenation pattern.

The Thiele hydrocarbon, the first‐ever synthesized stable para‐quinodimethane (pQDM) diradicaloid,^[^ ^1^ ^]^ remains the subject of intense studies more than a century after its discovery due to its intriguing properties and simple molecular architecture.^[^ ^2^, ^3^, ^4^ ^]^ Recently, highly fluorescent and photostable fluorinated and chlorinated Thiele hydrocarbons (**TFC** and **TTH** in Figure 1a,b) have been reported.^[^ ^5^, ^6^ ^]^ Both have exhibited impressive Stokes shifts (StS) and remarkable solvatochromism, despite their centrosymmetric and non‐polar molecular structure. This behavior has been ascribed to a symmetry breaking in the excited state. The latter is attributed to a mixing between the bright, singly excited (SE) state with the nearly degenerate and the formally dark, doubly excited (DE) state, resulting in a zwitterionic configuration (Figure 1c). Different degrees of mixing can lead to either an emissive charge‐transfer (CT) state or a nearly quenched charge‐separated (CS) state. Both scenarios are favored by molecular twisting along the elongated exocyclic C═C bonds, in analogy to the sudden polarization observed in olefins.^[^ ^7^, ^8^, ^9^ ^]^ Such photophysical properties render these chromophores particularly promising for photonic and optoelectronic applications.^[^ ^10^ ^]^ In this context, the zwitterionic nature of the excited state might imply the absence of closely lying triplet states, suggesting new potential applications in electroluminescent devices for these open‐shell singlet species.^[^ ^2^, ^11^ ^]^ A comprehensive understanding of the structure–property relationships in polyhalogenated Thiele hydrocarbons (PHTHs) remains, however, elusive. Despite similar photophysics, notable differences between **TFC** and **TTH** emerge. For instance, **TFC** exhibits a larger StS, exceeding 1 eV in cyclohexane, compared to 0.7 eV for **TTH**. The underlying difference becomes even more pronounced in polar solvents like tetrahydrofuran (THF), where **TFC** reaches a StS of 1.5 eV compared to 0.9 eV for **TTH**.

Perepichka and colleagues attributed this enhanced shift to the superior ability of fluorine substituents as they stabilize the photogenerated carbocation and carbanion via mesomeric and inductive effects, respectively. Although plausible, this explanation considers electronic effects but neglects steric hindrance as well as conformational effects introduced by different halogen substitution patterns. In this context, the magnitude of StS is influenced by the geometrical changes occurring upon excited‐state relaxation. According to time‐resolved spectroscopical studies on **TTH** and **TFC**, relaxation involves two steps. There is an initial structure relaxation along the bright excited state that does not involve symmetry breaking. This is followed by an exocyclic C═C twisting that breaks the symmetry and generates the emitting CT/CS state. In short, the formation of the CT/CS excited state depends on the twisting around the elongated exocyclic C═C bonds (note that excitation reduces the double bond character of exocyclic bonds), which the halogenation patterns may either promote or inhibit. Notably, the correlation between torsion angles and photophysics has been investigated for polyhalogenated trityl radicals.^[^ ^12^, ^13^, ^14^, ^15^, ^16^ ^]^ Studies for trityl radical‐based singlet diradicaloids remain limited.

This work aims to elucidate the roles of electronic as well as steric effects in PHTHs by synthesizing derivatives with different halogenation patterns. Specifically, 1,3,5‐trifluorobenzene and 1,3,5‐trichlorobenzene were utilized as peripheral substituents, linked to 2,3,5,6‐tetrachlorobenzene and 2,3,5,6‐tetrafluorobenzene as central bridges, respectively. The resulting **F,Cl‐TTH** and **Cl,F‐TTH** (Figure 1d) are at the forefront of this study. When considering the optical features of previously reported PHTHs alongside **F,Cl‐ TTH** and **Cl,F‐TTH**, their photophysics does not exhibit a simple linear trend that can be explained solely by halogen substitution. Consequently, we combined experimental and computational investigations and found that halogen substitution has a pronounced impact on structural variations in the ground state. This, in turn, impacts the bond length variation of the exocyclic C═C bonds upon photo‐excitation next to charge separation in the emitting state.

## Results and Discussion

**F,Cl‐TTH** and **Cl,F‐TTH** were synthesized with yields of 42% and 66%, respectively (Scheme S1a,b).^[^ ^17^ ^]^ The ^1^H‐NMR spectrum of **F,Cl‐TTH** revealed an almost free rotation of the peripheral phenyl rings around their axis at room temperature (Figures S3 and S4), whereas for **Cl,F‐TTH**, rotation was hindered due to steric effects (Figures S8 and S9) exerted from the four ortho‐chlorine atoms, becoming unrestricted only above 50 °C (Figure S10). The electronic effects facilitated the synthesis of both derivatives, allowing conversion of p‐xylene precursors to their dianionic forms with a slight excess of base (2.2 equiv) over a few hours. This contrasts with the previously reported **TTH**, which required a 15‐fold excess of base and four days of reaction time to achieve partial conversion. These observations highlight the role of fluorine atoms in stabilizing negative charges and reducing coulombic repulsion.^[^ ^18^ ^]^ Despite this advantage, the dianionic form of **F,Cl‐TTH** decomposed slowly in, for example, cyclic voltametric experiments (Figure S13). **Cl,F‐TTH** demonstrated reversible reductions at −1.18 and −1.60 V versus Fc/Fc^+^ and oxidation at +1.33 V versus Fc/Fc^+^, while **F,Cl‐TTH** just showed a reversible reduction at −1.28 V versus Fc/Fc^+^. The second reduction at −1.63 V versus Fc/Fc^+^ and oxidation at +0.97 V versus Fc/Fc^+^ resulted in decomposition byproducts. Prompt oxidation to the quinoidal form is necessary to maintain the **F,Cl‐TTH** structural integrity. Both adducts showed a remarkable thermal stability (Figure S14) that increases linearly by increasing the number of chlorine atoms.^[^ ^5^, ^6^, ^19^ ^]^

**Figure 2:**
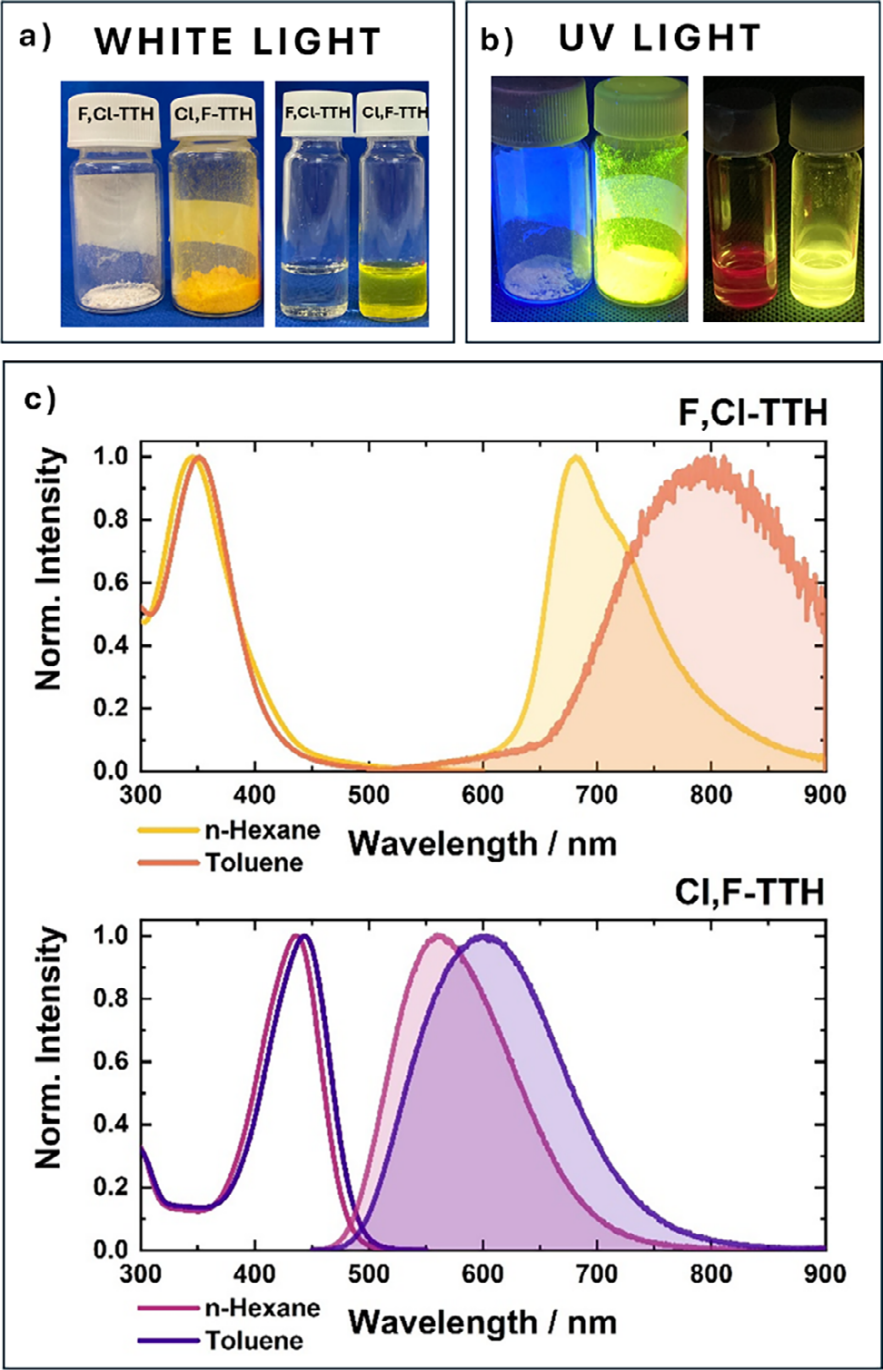
a) Photographs of **F,Cl‐TTH** and **Cl,F‐TTH** under white light illumination as a powder and in hexane solution; b) under UV light (365 nm) illumination; c) normalized absorption (non‐shaded) and emission spectra (shaded) for **F,Cl‐TTH** and **Cl,F‐TTH** in *n*‐hexane and toluene.

Figure 2a,b corroborate that subtle variations in the halogenation patterns induce significant differences in their photophysics. **Cl,F‐TTH** exhibits, for example, a solvent‐independent absorption maximum at around 440 nm (Figure 2c) and a molar extinction coefficient of 50 000 M^−1^ cm^−1^ (Figure S15). A blue shift is noted compared to chlorinated **TTH** and a red shift relative to fully fluorinated **TFC**. **F,Cl‐TTH** reveals, however, its lowest energy absorption in the ultraviolet at ∼350 nm and the molar extinction coefficient is as low as ∼20 000 M^−1^ cm^−1^ (Figure S15). This is again insensitive to the solvent environment. Here, the absorption maximum blue shifts compared to all other halogenated Thiele derivatives, **TTH**, **Cl,F‐TTH**, and even **TFC**. As such, the energetic position of the absorption maxima for PHTHs does not follow a straightforward trend depending on the number of chlorine versus fluorine atoms. To shed light on these absorption shifts, we investigated the molecular structure of the two PHTHs by synchrotron single‐crystal X‐ray diffraction (SCXRD) at 100 K. Details on data collection, structure solution, and refinement for **F,Cl‐TTH** and **Cl,F‐TTH** are reported in Supporting Information and summarized in Table S7.^[^ ^20^, ^21^ ^]^ Tables S8 and S9 for **F,Cl‐TTH**, Tables S10 and S11 for **Cl,F‐TTH** list refined fractional atomic coordinates, displacement parameters, bond distances and angles, torsion angles, and hydrogen bonds. **F,Cl‐TTH** and **Cl,F‐TTH** crystallized in *P*‐1 and *P*2_1_ */c*, respectively, with quinoidal configurations evident from the bond length alternation in the p‐xylylene framework. Exocyclic bond lengths of 1.3508(17) and 1.3522(16) Å for **F,Cl‐TTH** (see C3–C7 and C1–C4 in Table S8) and 1.382(3) Å for **Cl,F‐TTH** (see C6–C18 and C9–C30 in Table S10) indicate that steric hindrance drives the stretching in **Cl,F‐TTH**. A similar trend is seen in the quantum‐chemically optimized ground state structures (Figures S46–S48). The computed exocyclic C═C bond for **F,Cl‐TTH** is shorter.

**Figure 3:**
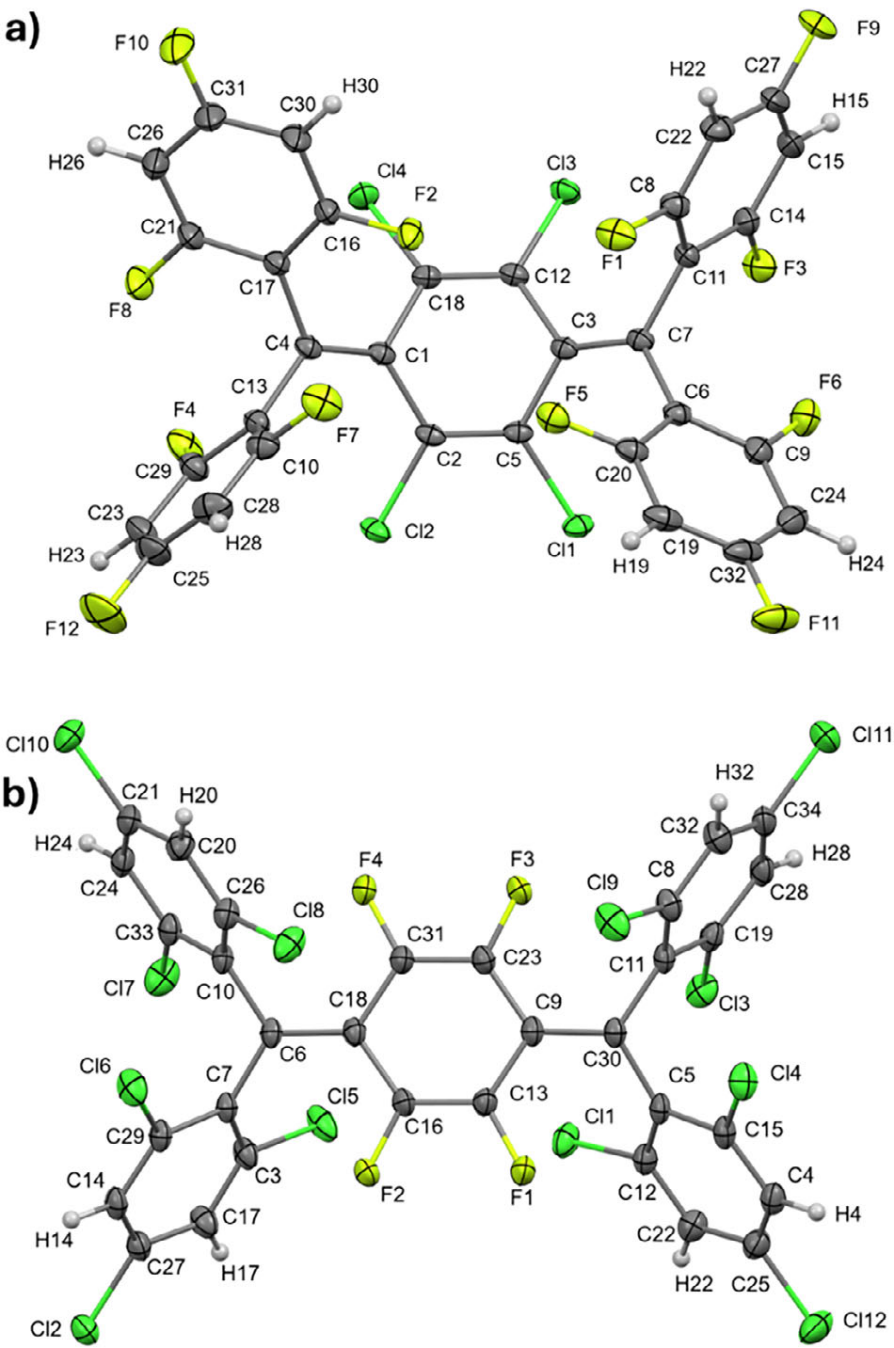
A view of the asymmetric unit of **F,Cl‐TTH** a) and **Cl,F‐TTH** b) showing the atom‐labeling scheme and the color setting by atomic species (i.e., green, yellow, grey, and white for Cl, F, C, and H atoms, respectively). In the case of **Cl,F‐TTH**, the hexane solvent is omitted for clarity. Ellipsoids are drawn at 50% probability level.

**Figure 4:**
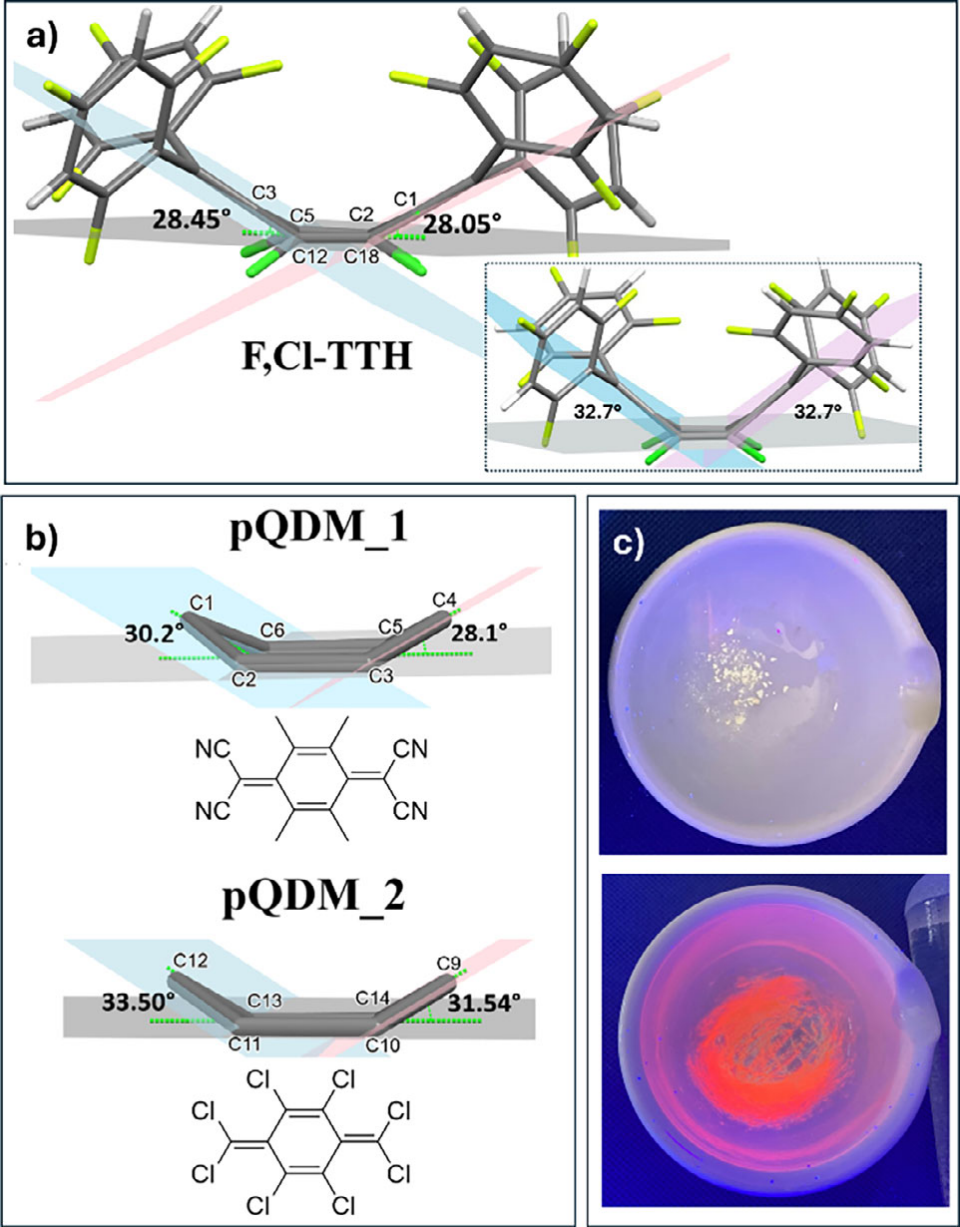
a) The folded structure of **F,Cl‐TTH** from SCXRD; inset: DFT (M062X‐D3/def2SVP) optimized ground state structure of **F,Cl‐TTH**; b) similarly folded pQDM derivatives. Structures retrieved from the CSD; c) photograph of **F,Cl‐TTH** as a powder under UV light (365 nm) before (top) and after (bottom) grinding.

Notably, the enhanced quinoidal structure is associated with a remarkably reduced diradical character, which is quantified by the diradical index and ranges from 0 for a closed‐shell structure to 1 for a pure diradical. The calculated values were *y*_0_ = 0.093 for **F,Cl‐TTH** and *y*_0_ = 0.275 for **Cl,F‐TTH** (Table S12). In the case of **Cl,F‐TTH**, structure determination also revealed the unexpected presence of hexane. A view of the asymmetric unit of **F,Cl‐TTH** and **Cl,F‐TTH** is shown in Figure 3a,b, respectively. The distortion in the central bridge of **Cl,F‐TTH** was minimal, with a nearly planar core ring geometry and bending angles (BAs) of 2.62° and 3.70° (see Supporting Information for additional details). Similarly, a slight distortion was noted for **TTH** with BAs of 2.51° and 8.08°. In stark contrast, **F,Cl‐TTH** adopted a folded boat conformation, with significant deviations from planarity. This is quite uncommon for derivatives having a single phenyl ring in the pQDM bridge.^[^ ^22^, ^23^ ^]^ The BAs between the reference plane defined by atoms C2, C5, C12, and C18 (light grey in Figure 4a) and the inclined planes defined by C3, C5, and C12 (light‐blue plane in Figure 4a) and C1, C2, and C18 (pink plane in Figure 4a) were 28.45° and 28.05°, respectively. These values are comparable to those observed in only two known examples of pQDMs, that is, **pQDM_1** ^[^ ^24^ ^]^ and **pQDM_2** ^[^ ^25^, ^26^, ^27^ ^]^ (Figure 4b), based on their crystal structures retrieved from the Cambridge Structural Database (CSD)^[^ ^28^ ^]^ and identified using the software Mogul^[^ ^29^ ^]^ (additional details can be found in the Supporting Information). The DFT‐optimized geometries of **F,Cl‐TTH** (Figure 4a inset) and of the two pQDM derivatives (Figures S47–S49) align well with the experimentally determined folded structures, with predicted BAs of 32.7°, 29.4°, and 33.5° for **F,Cl‐TTH**, **pQDM_1**, and **pQDM_2**, respectively. Such bending strongly impacts the conjugation and blue shifts the absorption of **F,Cl‐TTH**.

It represents, to the best of our knowledge, the first report for a folded derivative that shares the same conjugated skeleton as Thiele's hydrocarbon. In contrast, most known folded hydrocarbons feature an anthraquinodimethane scaffold.^[^ ^30^ ^]^ Notably, Feringa et al. reported a Thiele hydrocarbon with a bent boat configuration, in which the distortion did not affect the central p‐xylylene unit but the exocyclic bis‐thioxanthene substituents.^[^ ^31^ ^]^ More recently, a folded Thiele hydrocarbon dimer nanoring, bearing an anthraquinodimethane bridge, has been reported by Nishiuchi et al.; however, its optical properties considerably differ from Thiele's hydrocarbon ones.^[^ ^32^ ^]^ The crystal packing of **F,Cl‐TTH** revealed the presence of several cooperative non‐covalent interactions^[^ ^33^, ^34^, ^35^, ^36^, ^37^ ^]^ (see the Supporting Information for additional details and Figures S43 and S44). They assist in stabilizing the crystal structure and contribute (Figure S44) to the distortion of the central ring, mainly through halide–halide interactions.^[^ ^33^, ^34^, ^35^ ^]^ Comparing **F,Cl‐TTH**, **pQDM_1**, and **pQDM_2** suggests that the boat‐like rearrangement is primarily driven by the electron‐withdrawing nature of the peripheral substituents. The diradical character is, however, influenced by the degree of conjugation and is larger for more extensively conjugated frameworks. Thus, the computed values of 0.093, 0.149, and 0.057 for **F,Cl‐TTH**, **pQDM_1**, and **pQDM_2**, respectively, agree well with the fact that the structures are folded.

Comparing the emission of the two PHTHs reveals several trends. For **Cl,F‐TTH**, the emission is solvatochromic, namely ranging from 560 nm in hexane with a quantum yield (QY) of 100% up to 652 nm in dichoromethane and a QY of 58%. Hereby, the emission is blue‐shifted relative to **TTH** (as can be expected due to electronic effects determined by partially replaced chlorine atoms with fluorines) and to **TFC**. StSs are similar to those observed for **TTH**, that is, 0.63 eV in hexane and 0.97 eV in dichloromethane. Relative to **Cl,F‐TTH**, **F,Cl‐TTH** displays a red‐shifted emission at 681 nm in hexane with a QY of 65% and a lifetime of 24.9 ns. The StS is remarkable, with a value of 1.78 eV (337 nm). StSs even further increase when changing the solvent polarity. For example, the emission of **F,Cl‐TTH** red‐shifts in toluene to 791 nm and in chloroform to 808 nm, where the StS is as large as 2.05 eV (462 nm) (Figure 2c). Such a StS exceeds even that seen for **TFC** with its fully fluorinated halogenation pattern. As a consequence, the QY drops to 1.1% in toluene and <1% in chloroform. Going beyond chloroform, like dichloromethane, **F,Cl‐TTH** is virtually non‐emissive. Overall, **F,Cl‐TTH** is far more solvent sensitive than its analogue PHTHs. Regarding the photostability, both derivatives display remarkable photostability, showing no signs of photodegradation after 1 h of continuous UV irradiation (Figure S17).

The emissive properties do not exhibit a systematic correlation with the number of chlorine or fluorine substituents, suggesting that additional electronic and/or structural factors must be considered at this stage. A “giga” StS in molecular systems is typically linked to substantial alterations in the molecular or electronic structure between the Franck–Condon excited state and the relaxed excited state. To this end, structural reorganizations in the excited state are followed by energy lowering, driven by the formation of a zwitterionic twisted CT emissive state.^[^ ^38^ ^]^ In PHTHs, the initial relaxation takes place in the SE state, followed by mixing with the formally dark DE state. Key is, on one hand, their near degeneracy and, on the other hand, the twisting around the remarkably elongated C═C bonds. This depends, hereby, on the extent of geometry relaxation in the SE state. The key role of structural reorganizations becomes evident in temperature‐dependent fluorescence measurements. In the case of **Cl,F‐TTH**, a decrease in temperature leads to a continuous blue‐shift from, for example, 563–555 nm when cooling from room temperature to 160 K (see Figures S19–S24 for temperature‐dependent spectroscopic experiments). Notably, the blue‐shift is much stronger once a frozen matrix is formed, that is, at 80 K, where the blue‐shift reaches 492 nm. In stark contrast, the absorption is subject to minor red shifts and to an evolution of vibronic fine structure when lowering the temperature. Consequently, the StS is reduced from 0.63 eV in 3‐methylpentane at room temperature down to 0.27 eV at 80 K. At the same time, the fluorescence lifetime is reduced from 12.6 to 2.2 ns. This temperature dependence is a consequence of a hindered relaxation. Turning to **F,Cl‐TTH**, a similar trend is observed. In fact, the lifetime decreases from 29.5 ns at room temperature to 20.4 ns at 120 K. For **F,Cl‐TTH**, a slight red‐shift of the 683 nm emission maximum to 699 nm is obtained upon lowering the temperature. In combination with a minor blue shift of the absorption, the StS of 1.80 eV remains unaffected within this temperature range. Interestingly, the absorption spectra lack any fine structure, a finding that is rationalized by the fact of co‐existing conformers for **F,Cl‐TTH** (for **F,Cl‐TTH** two stable structures were computed having C_2_ (Figure S47) and C_s_ (Figure S48) symmetry). This situation changes, however, at cryogenic temperatures of 80 K. Here, a blue‐shift to 680 nm together with a substantially quenched fluorescence intensity sets in. Beyond that, the fluorescence decay is biexponential with lifetimes of 3.9 and 18.1 ns. We conclude that any structural reorganizations are significantly slowed down and, therefore, become observable in the fluorescence profiles. Moreover, a newly emerging emission at around 465 nm is noted. We attribute this emission to an initially populated, but short‐lived, SE state. As a matter of fact, the fluorescence lifetime in this spectral region is likely to be shorter than the temporal resolution of our experimental setup. Overall, the temperature‐dependent emission of **F,Cl‐TTH** is found to be like that previously reported for **TTH**. But, any temperature effects on the emission are much weaker. Our experimental data suggest that for **F,Cl‐TTH** structural reorganizations are still operative at 80 K. Altogether, the temperature dependence highlights the impact of structural relaxation on the fluorescence mechanism of the studied PHTHs, which are key to allowing for mixing with the formally dark DE state and to aligning toward near degeneracy.

TDDFT‐optimized geometries of the SE state show that both **Cl,F‐TTH** and **F,Cl‐TTH** relax to afford planar structures (Figures S51–S53). However, the degree of their geometric and energetic relaxations differs significantly. The reorganization energy associated with the SE state relaxation is with 0.45 eV for **Cl,F‐TTH** remarkably smaller compared to 1.61 eV for **F,Cl‐TTH**. Considering the relaxed geometry, the increase in the exocyclic C═C bond length parallels the relaxation energy. It changes from 0.058 Å for **Cl,F‐TTH** to 0.109 Å for **F,Cl‐TTH**. Both reorganization energies and exocyclic C═C bond lengths agree well with the StS magnitude in hexane: 0.63 eV for **Cl,F‐TTH** and 1.78 eV for **F,Cl‐TTH**. What contributes to StS, at least in non‐polar solvents, is the relaxation in the SE state. It is worth pointing out that **Cl,F‐TTH** features a shorter exocyclic C═C bond in the excited state than **F,Cl‐TTH** (Figures S51–S53), despite having a longer bond in the ground state. This indicates that the excited‐state bond length is not directly correlated with its ground‐state value but rather reflects the degree of electronic reorganization upon excitation. In **Cl,F‐TTH**, the overall reorganization is limited by the more delocalized nature of the excitation. In the second step of the excited‐state relaxation, mixing of the SE and DE states, which governs the degree of charge separation, is essential. Both SE and DE are charge resonance states.^[^ ^39^ ^]^ Their mixing leads to the emergence of a net charge separation. If mixing is, however, incomplete, charge separation will be largely suppressed and the solvatochromic effect will be weaker, as in the case of more extended polyhalogenated Chichibabin and Müller hydrocarbons.^[^ ^39^, ^40^, ^41^, ^42^ ^]^ In terms of the energy gap spanning between the SE and DE states, the elongated exocyclic C═C bond in the relaxed SE state affects the energy gap between SE and DE states. Longer bonds promote near‐degeneracy. This near‐degeneracy, in turn, enhances state mixing and facilitates the formation of CS states. Independent confirmation for this trend came from CASSCF + NEVPT2 calculations performed at the relaxed SE geometry (Tables S13 and S14). The SE/DE energy difference is ca. 0.4 eV larger for **Cl,F‐TTH**, consistent with its less elongated exocyclic C═C bond, whereas it is significantly smaller for **F,Cl‐TTH** (ca. 0.1 eV), which features the longest exocyclic bond in the excited state. Consequently, state mixing is less effective in **Cl,F‐TTH**. It reduces the charge separation efficiency and explains the weaker solvatochromic response next to the higher QYs of **Cl,F‐TTH**, relative to **F,Cl‐TTH**. **F,Cl‐TTH** exhibits a more effective state mixing owing to the near‐degeneracy of the SE and DE states. It enhances the charge separation and explains the stronger solvatochromic response observed for **F,Cl‐TTH**. The charge distribution in both the ground and SE states of the two adducts was analyzed to assess the quadrupolar character of the new PHTHs and to gain deeper insight into the relationship between electronic effects and conformational changes. Details are provided in Figure S56 and Table S15. The results confirm the A–D–A nature of **F,Cl‐TTH** and the D–A–D nature of **Cl,F‐TTH**. Interestingly, in the SE state, the electronic excitation, accompanied by the elongation of the exocyclic C═C bonds, leads both derivatives to adopt a D–A–D configuration. While **Cl,F‐TTH** is fluorescent as a powder (Figure 2b), F**,Cl‐TTH** is a white non‐emissive solid, that can be converted into an emissive form upon application of mechanical stress (Figure 4c), reverting to a non‐fluorescent state after solvent annealing. We hypothesized that this solid‐state fluorescence may originate from a metastable planar form of the molecule. The computed structure (Figure S55, details in the Supporting Information) lies 7.4 kcal mol^−1^ above the C_2_ minimum, and vibrational frequency analysis indicates that, as an isolated molecule, it corresponds to a saddle point. Although this structure is not stable in solution, it may become a metastable state in the solid phase. The charge distribution analysis for this planar structure (**F,Cl‐TTH** (saddle) in Table S15) shows a reduced A–D–A character. The mechanofluorochromism, and more broadly the solid‐state photophysics of PHTHs, are currently under investigation.

**Figure 5:**
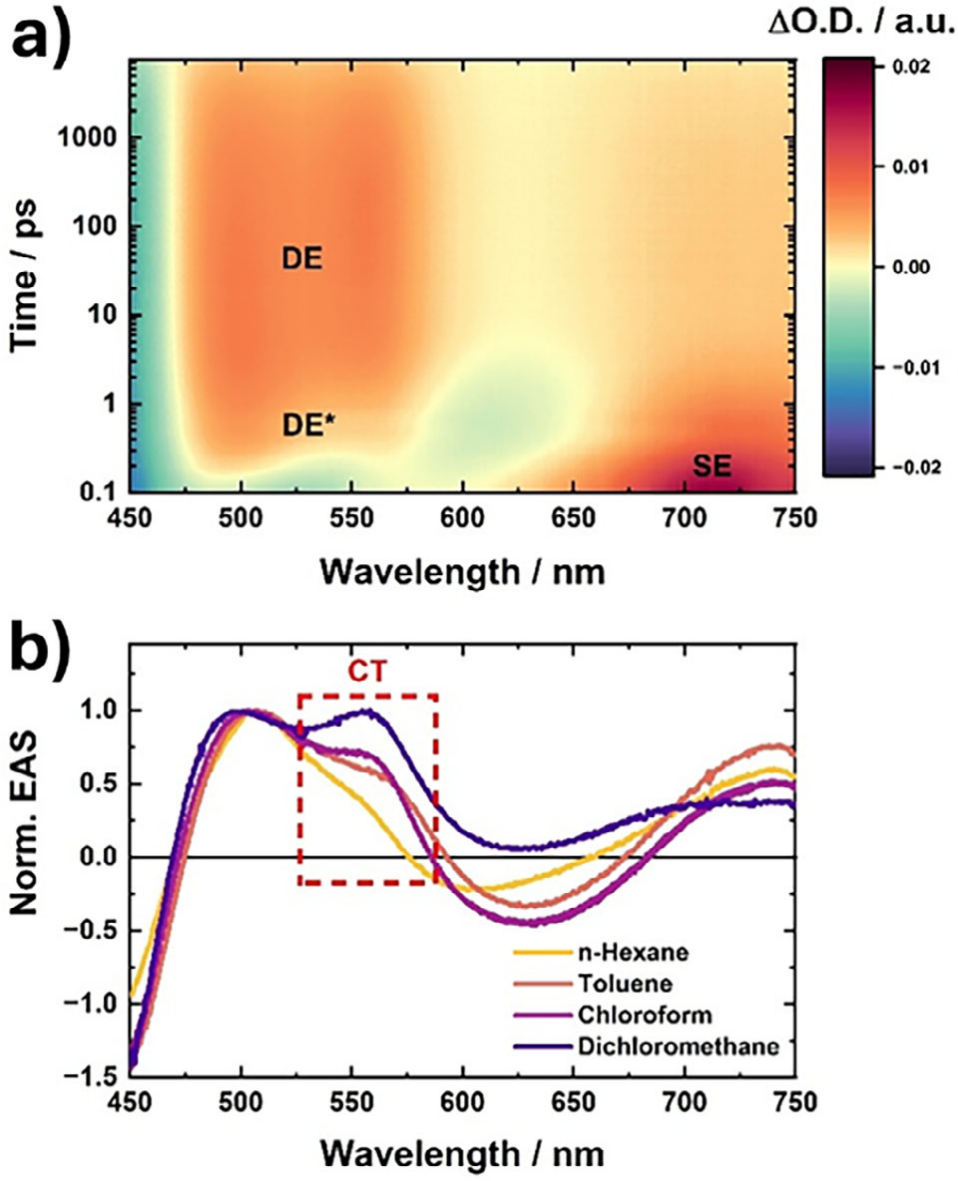
a) Representative fs‐transient absorption (TA) contour plot of **Cl,F‐TTH** in dichloromethane obtained upon photoexcitation at 440 nm. TA data are time‐zero as well as chirp corrected. Obtained and assigned excited‐state species are indicated. b) Normalized evolution‐associated spectra (EAS) of the deconvoluted DE species obtained via sequential global fitting of the fs‐TA raw data measured in different solvents with highlighted CT signatures.

To gather insights on the excited‐state dynamics of both **Cl,F‐TTH** and **F,Cl‐TTH**, we performed transient absorption (TA) spectroscopy with both femtosecond and nanosecond time resolution. Starting with **Cl,F‐TTH**, an overall similar trend to **TTH** and **TFC** is seen following photo‐excitation (Figure 5a). First, photo‐excitation leads to the population of the SE state with excited state absorption (ESA) located at 710 nm together with ground‐state bleaching (GSB) and stimulated emission at around 500 nm. Notably, stimulated emission from the SE state exerts only a minor impact on the observed emission due to an ultrafast internal conversion, as already outlined by Liu et al. for **TFC**.^[^ ^5^ ^]^ Second, the transition to the unrelaxed DE* state starts with ESAs at 500 and 730 nm. The spectral characteristics of the SE and DE* states are solvent independent and underline the absence of a net charge separation. The subsequent DE* state relaxation induces twisting and results in the formation of a 560 nm ESA. It is attributed to a CT state that is a consequence of SE/DE state mixing. An energetic stabilization of the CT state is reflected by the red‐shift of the stimulated emission in the spectral region between 560 and 660 nm. The shift of stimulated emission and the evolution of CT features both depend on the surrounding solvating shell. They are both more pronounced in polar solvents like dichloromethane. The net effect is the stabilization of the CT state (Figure 5b). In contrast to **TTH**, both internal conversion and relaxation processes proceed for **Cl,F‐TTH** and **TFC** significantly faster in the sub‐picosecond and picosecond time domains, respectively (see Table S5 for a summary of all time constants). Here, the less sterically demanding fluorine groups facilitate structural reorganizations, in general, and twisting around the exocyclic bonds, in particular. Beyond these considerations, the initially longer exocyclic bonds constitute a nuclear geometry that is favorable for a faster conversion and relaxation. Subsequent to these initial ultrafast processes, we note the decay of the relaxed DE state with lifetimes of 13.2 ns in *n*‐hexane and 9.59 ns in dichloromethane. Interestingly, QYs, DE lifetimes, and solvent polarities do not strictly correlate with each other. For **Cl,F‐TTH**, all lifetimes are substantially shorter when compared to those for **TTH**, despite higher or similar QYs.

**Figure 6:**
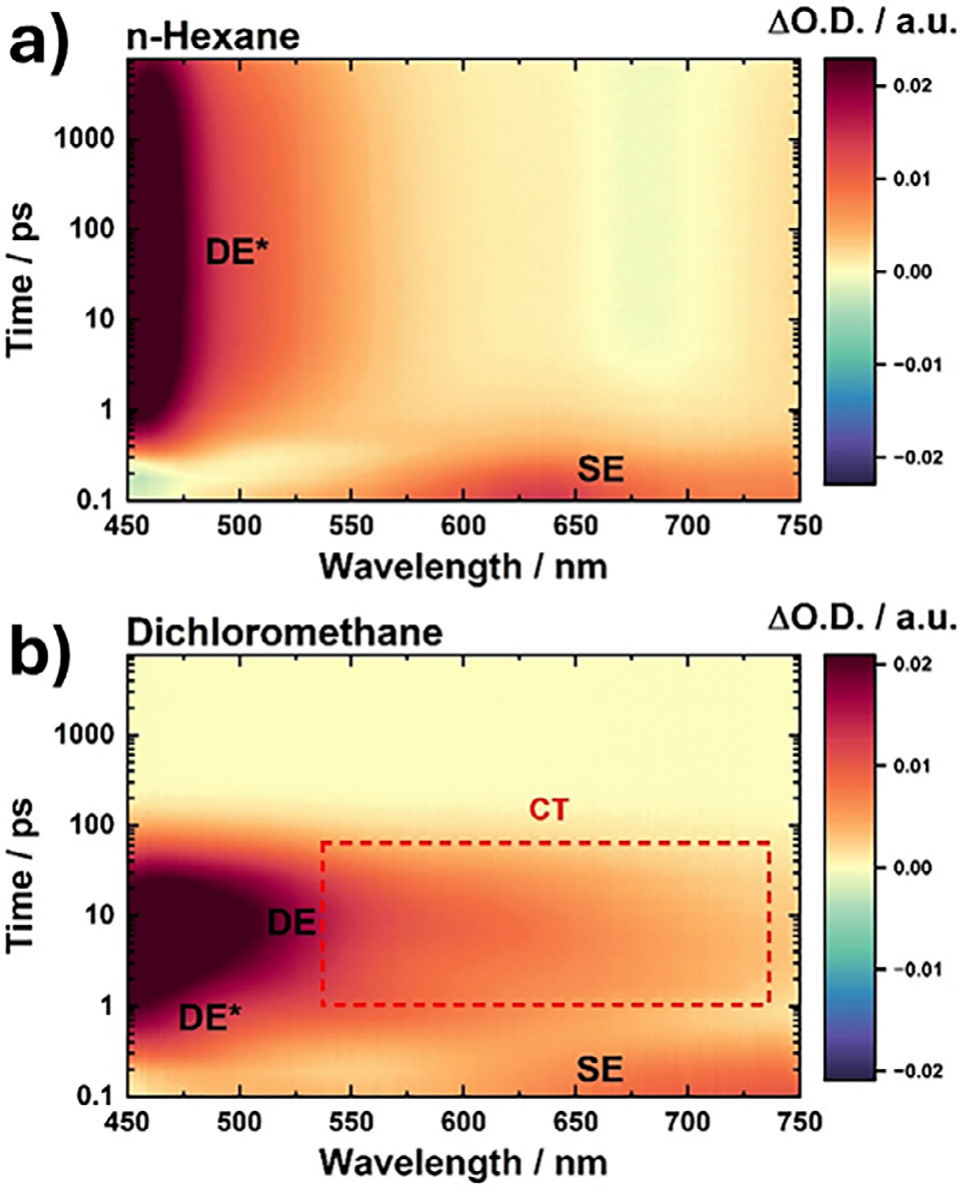
Representative fs‐transient absorption (TA) contour plot of **F,Cl‐TTH** in a) *n*‐hexane and b) dichloromethane obtained upon photoexcitation at 360 nm. TA data are time‐zero as well as chirp corrected. Obtained and assigned excited‐state species are indicated with the CS signature highlighted in (b).

A decreased steric hinderance and a suitable geometry are likely to be responsible for a more efficient internal conversion back to the electronic ground state. Of great relevance is the SE/DE state mixing mechanism to create the transition dipole moment that drives radiative decay. Turning to **F,Cl‐TTH**, substantial differences evolved in the excited deactivation pattern (Figure 6a). Initially, the SE state is populated, with stimulated emission at round 450 nm and a broad ESA in the red part of the spectrum. What follows is the ultrafast population of the DE* state. In *n*‐hexane, no DE state relaxation is seen. Indeed, **F,Cl‐TTH** is effectively trapped in the unrelaxed DE* configuration. Evidence for this stems from the lack of any spectral shifts in the 450 nm ESA and 680 nm stimulated emission. As such, a non‐polar solvent such as *n*‐hexane inhibits any stabilization of the DE* state. The situation changes, however, when using toluene or other solvents of higher polarity. Now, a stabilization of the DE state renders possible by the solvent environment. This is seen in the form of a red‐shifted 450 nm ESA next to the evolution of a broad ESA spreading between 520 and 750 nm. The latter feature is highly relevant as it reflects the pronounced CT/CS character of the relaxed excited state. Implicit is again a twisting‐induced SE/DE state mixing, which is now activated in these solvents. For **F,Cl‐TTH**, a number of important conclusions should be highlighted. First, the CT band is significantly broadened when compared to that for **Cl,F‐TTH** in toluene, chloroform, as well as dichloromethane. A likely rationale are the different conformers with C_2_ and C_s_ symmetry of **F,Cl‐TTH**, contributing to the overall ESA bandwidth. Also, the involvement of a CS state rather than a CT state seems to play a significant role. Lower QYs and smaller SE/DE energy gaps enable a more efficient state mixing. This, in turn, favors a fully CS state rather than only a CT state. Second, the lifetime of the DE state is much shorter in solvents other than *n*‐hexane with 23.3 ns and instead found in the picosecond regime like, for example, dichloromethane with 52.0 ps. Formation of a zwitterionic CS state in solvents when going beyond *n*‐hexane makes the difference. It is impossible to link such an increase in deactivation to just the energy gap law dependence of non‐radiative decay, especially considering that **F,Cl‐TTH** and **TTH** emit in the same spectral region and, at the same time, reveal substantially longer excited‐state lifetimes. Instead, the fast ground state recovery must be associated with a charge recombination of the CS state. In summary, the observed emission behavior strongly supports the hypothesis that (i) sudden polarization is the underlying mechanism driving the solvatochromic emission in both derivatives, consistent with previous findings for **TTH** and **TFC** and (ii) halogen substitution can modulate steric and electronic effects to favor a “giga” StS by enabling large reorganization energies in the photoexcited state, followed by efficient formation and stabilization of the mixed zwitterionic CT/CS state.

## Conclusions

In this work, we have synthesized and characterized two new halogen‐substituted Thiele hydrocarbons, **Cl,F‐TTH** and **F,Cl‐TTH**, which exhibit markedly distinct structural and photophysical properties despite their similar substitution patterns. The steric constraints imposed by the ortho‐chlorine atoms in **Cl,F‐TTH** stabilize a planar geometry, while **F,Cl‐TTH** adopts an uncommon folded boat conformation in the solid state, never observed before for a Thiele hydrocarbon. These structural differences directly impact their diradical character, excited‐state relaxation, and dynamics, leading to divergent optical signatures. **Cl,F‐TTH** displays high fluorescence quantum yields and moderate Stokes shifts (the smallest reported for PHTHs), consistent with a more delocalized electronic structure and the limited excited‐state reorganization predicted by quantum‐chemical calculations. In contrast, **F,Cl‐TTH** shows UV absorption, extremely large Stokes shift, and pronounced solvent‐dependent emission, arising from extensive geometric relaxation and efficient mixing between SE and DE states, which stabilizes a zwitterionic emissive state. In addition, the folded Thiele hydrocarbon exhibits pronounced mechanofluorochromism and shows strong potential as multi‐responsive molecular materials, as it can switch from a non‐aromatic folded structure to an antiaromatic planar form upon mechanical stress and to an aromatic zwitterionic configuration upon photoexcitation. Considering these unique features, such derivatives can find application in several fields such as photonics,^[^ ^43^ ^]^ fluorophores for luminescent solar concentrators,^[^ ^44^ ^]^ sensing,^[^ ^45^ ^]^ anti‐counterfeiting materials,^[^ ^46^ ^]^ strain, and light‐responsive electronics.^[^ ^47^, ^48^, ^49^ ^]^ In conclusion, these findings underscore how minimal variations in the halogenation pattern profoundly alter the balance between steric and electronic effects in Thiele hydrocarbons. This, in turn, governs their ground‐ and excited‐state properties, enabling control over charge separation and emission behavior. The results establish halogen‐substituted Thiele hydrocarbons as a versatile platform for the design of next‐generation functional organic materials with tailored photophysical and electronic properties and provide new insights on the molecular design of fluorophores having an open‐shell configuration based on pQDM scaffold.

## Supporting Information

Synthetic details, NMR spectra and analysis, cyclic voltammetry, thermogravimetric analysis, spectroscopic characterization, crystallographic structure analysis, computational details. Deposition Numbers 2492420 (for **F,Cl‐TTH**), 2492424 (for **Cl,F‐TTH**), contain the supplementary crystallographic data for this paper. These data are provided free of charge by the joint Cambridge Crystallographic Data Centre and Fachinformationszentrum Karlsruhe Access Structures service. The authors have cited additional references within the Supporting Information.^[^ ^50^, ^51^, ^52^, ^53^, ^54^, ^55^, ^56^, ^57^, ^58^, ^59^, ^60^, ^61^, ^62^, ^63^, ^64^, ^65^, ^66^, ^67^, ^68^, ^69^, ^70^ ^]^

## Author Contributions

Angela Punzi, Davide Blasi, and Davide Mesto performed synthetic work, NMR analysis, TGA, and cyclic voltammetries under the coordination of Gianluca M. Farinola; Vincent Olieric and Sylvain Engilberge collected SC‐XRD data; Anna Moliterni and Cinzia Giannini performed the structural analysis. Tobias Ullrich collected optical data and performed their analysis under the supervision of Dirk M. Guldi; Michele Orza performed computational calculations under the supervision of Fabrizia Negri; Davide Blasi conceptualized the project and together with Fabrizia Negri and Dirk M. Guldi, they wrote the first version of the manuscript. All authors discussed, commented, and revised the manuscript. All authors have given approval to the final version of the manuscript.

## Conflict of Interests

The authors declare no conflict of interest.

## Supporting information



Supporting Information

Supporting Information

## Data Availability

The data that support the findings of this study are available from the corresponding author upon reasonable request.
