# Supplementary material for: Twist and Shine: The Impact of Halogen Substitution on Thiele Hydrocarbon's Optical Properties
Source: Angew Chem Int Ed Engl. 2025 Dec 17;65(6):e24043. doi: 10.1002/anie.202524043 (PMC12865251; doi:10.1002/anie.202524043)
Supplement: Supplementary file 1 — Supporting Information [file ANIE-65-e24043-s002.docx]

**Supporting Information**

Twist and Shine: The Impact of Halogen Substitution on Thiele Hydrocarbon’s Optical Properties

Angela Punzi,^[a]^ Tobias Ullrich,^[b]^ Michele Orza,^[c,d]^ Davide Mesto,^[a]^ Anna Moliterni,^[e]^ Vincent Olieric,^[f]^ Sylvain Engilberge, ^[g]^ Cinzia Giannini,^[e]^ Fabrizia Negri,*^[c,d,h]^ Dirk Guldi,*^[b]^ Davide Blasi*^[a]^ and Gianluca M. Farinola^[a]^

[a] Dipartimenti di Chimica, Università degli Studi di Bari Aldo Moro, Via E. Orabona 4, 70125, Bari, Italy

[b] Department of Chemistry and Pharmacy and Interdisciplinary Center for Molecular Materials (ICMM), Friedrich-Alexander-University Erlangen-Nuremberg, 91058 - Erlangen, Germany

[c] Dipartimento di Chimica “Giacomo Ciamician”, Università di Bologna and INSTM UdR Bologna, Via Piero Gobetti 85, 40129, Bologna, Italy

[d] Center for Chemical Catalysis—C3, Università di Bologna, 40129 Bologna, Italy

[e] Istituto di Cristallografia, CNR, Via Amendola, 122/O, Bari, 70126, Italy

[f] Swiss Light Source, Paul Scherrer Institute, Villigen PSI, Forschungsstrasse 5232, Switzerland

[g] Université Grenoble Alpes, CNRS, CEA, Institut de Biologie Structurale, Grenoble, France

[h] INSTM UdR Bologna, 40129 Bologna, Italy

Contents

[General Methods S2](#_Toc201241731)

Synthesis…………………………………………………………………………………………………………………………………………………….S4

[Electrochemical and Thermal Characterization…………………………………………………………………………………………S19](#_Toc201241732)

Spectroscopic Characterization…………………………………………………………………………………………………………………S20

[Structure determination from synchrotron SCXRD S42](#_Toc201241733)

[Computational details S60](#_Toc201241734)

[References S73](#_Toc201241735)

# General Methods

All chemical reagents and solvents were purchased from commercial suppliers and used without further purification (unless otherwise stated). Tetrahydrofuran (THF) was distilled from sodium-benzophenone before used.

Column chromatography was performed with Macherey-Nagel flash silica gel (60, particle size 0.04−0.063 mm). Macherey-Nagel aluminum sheets with silica gel 60 F254 were used for TLC.

The ^1^H NMR spectra were recorded with an Agilent 500 NMR spectrometer for samples in CDCl_3_ and THF-d_8_ at room temperature or deuterated C_2_D_2_Cl_4_ in temperature range between 298-343K, using the CDCl_3_ residual proton peak at δ = 7.26 ppm, the THF-d_8_ residual proton peak at δ = 3.58 ppm or the C_2_D_2_Cl_4_ residual proton peak at δ = 6.00 ppm as internal standard. ^19^F NMR spectra were acquired on an Agilent 500 spectrometer at 470 MHz for samples in CDCl_3_, THF-d_8_ or C_2_D_2_Cl_4_ at room temperature. ^13^C NMR spectra were acquired on an Agilent 500 spectrometer at 126 MHz for samples in CDCl_3_ or C_2_D_2_Cl_4_ at room temperature, using the signals of CDCl_3_ at δ = 77.16 ppm or the signal of C_2_D_2_Cl_4_ at 73.78 ppm as internal standard.

High-resolution mass spectra were acquired with a Shimadzu high-performance liquid chromatography ion trap time-of flight (LC-IT-TOF) mass spectrometer *via* direct infusion of the samples by using methanol as the elution solvent (the samples were previously dissolved in THF, and few drops of the THF solution were added to methanol).

Thermal gravimetric analysis (TGA) was carried out on a Pyris 1-Perkin Elmer thermogravimetric analysis system, applying a nitrogen flow of 40 mL/min. Sample was maintained at 30°C for 5 min and then it was heated at a heating rate of 5°C min^−1^ in the range of temperature 30-500°C.

The cyclic voltammetry (CV) measurements were performed using an Autolab potentiostat (model PGSTAT128N) by Metrohm. A glassy carbon electrode was used as the working electrode. Platinum wires acted as the counter reference electrodes, together with the redox couple ferrocenium/ferrocene as external standard. The CV profiles were recorded at the rate of 100 mV·s^–1^. Tetrabutylammonium hexafluorophosphate in anhydrous dichloromethane (0.1 M) was used as the supporting electrolyte.

Single-crystal X-ray diffraction (SCXRD) data measurements were carried out at the beamline BM07 of the ESRF, Grenoble, France, using the 16-bunch mode of the ESRF machine at 64.1 mA. Data collection was performed at both low temperature (T=100 K) and ambient temperature (T=293 K) with a 250 × 200 µm^2^ beam at an energy of 18.048 keV (λ = 0.6869 Å) on selected crystals mounted on litholoops (Molecular Dimensions). For each crystal, five 360° data sets were collected in shutterless mode in steps of 0.5° at a speed of 0.1 s/step at Kappa angles of 0º, 10º, 20º, 40º and 60º on a Dectris Ltd. PILATUS 6M detector. Diffraction data were processed, corrected for absorption, and merged using XDS/XSCALE.^1^ The crystal structures were solved by Direct Methods^2^ using *SIR2019*^3^ and refined with *SHELXL2014/7.*^4^ Additional applied computer programs: *WinGX* ^5^ and *publCIF* ^6^ for preparing the published material, *Mercury*^7^ for molecular graphics and the CCDC (Cambridge Crystallographic Data Center) software *Mogul*^8^ for retrieving molecular geometry information. CIF files were validated using the IUCr web-based checkCIF service.^9^

Steady-state absorption was performed using a Shimadzu UV-1900i UV-Vis double-beam spectrometer (190 to 1100 nm). Steady-state emission spectra were acquired using an FS5 spectrofluorometer from Edinburgh Instruments. PLQYs were measured in a spectral window up to 950 nm with the SC-30 integrating sphere mounted on the FS5 spectrofluorometer. Additionally, PLQYs were double checked upon plotting the integrated emission areas *vs*. optical densities at different concentrations. PLQYs were then obtained by referencing to a suitable emissive dye and the refractive index of the solvents was taken into account. Time-resolved emission was measured with a FS5-TCSPC spectrofluorometer from Edinburgh Instruments equipped with a photomultiplier R928P emission detector. Samples of **F,Cl-TTH** were excited at 355 nm by a VISUV versatile picosecond laser module from Picoquant, whereas **Cl,F‑TTH** was excited using a SuperK Fianium FIU6PP supercontinuum laser from NKT Photonics. The repetition rate was 8 MHz and the temporal resolution of the experimental set up was 200 ps. Temperature dependent absorption, emission and TCSPC spectra were recorded with the FS5-TCSPC spectrofluorometer from Edinburgh Instruments using a cryostat Optistat DN from Oxford Instruments.

All fs- and ns-TA spectra were performed employing an Astrella-F-1K amplified Ti:sapphire femtosecond laser system from Coherent Inc. as pump laser source, operating at a repetition rate of 1 kHz, with an 800 nm output and pulse duration of 80 fs, reaching an output power of 5.5 W (5 mJ pulse energy). A fraction of 1.2 mJ was utilized for excitation pulse generation by a Topas Prime with standard NirUVis extension module from Light Conversion. Energy per excitation pulse was reduced to 400 nJ. Another fraction of 0.15 mJ was used for probe pulse generation and guided through a motorized delay line, achieving a temporal delay window of up to 8.0 ns. Both excitation and probe beams were guided to a customized TA pump/probe detection system from Ultrafast systems Inc.: Helios, Eos. Polarization of the excitation pulse was set to magic angle in respect to the probe pulse to avoid presence of rotational anisotropy dynamics. The concentration was chosen to achieve an optical density of around 0.2 in the spectral region of the excitation wavelength (360 and 440 nm for **F,Cl-TTH** and **Cl,F‑TTH**, respectively). Quartz cuvettes with 2 mm optical path length were used for TA experiments and solutions were continuously stirred with magnetic stirring bars to avoid photodegradation. Obtained data were treated by SVD and global analysis using the R- package TIMP and GloTarAn.^10-12^ The instrument response function (IRF) and dispersion (chirp of the white light pulse) were modelled and taken into account during the fitting procedure. TA data are presented after careful background, chirp as well as time-zero correction.

**Synthesis**

**Scheme S1.** Overall synthetic procedures for **F,Cl-TTH** and **Cl,F-TTH**.

***2,2',2'',2'''-((Perchloro-1,4-phenylene)bis(methanetriyl))tetrakis(1,3,5-trifluorobenzene)*** **2**.

A 25 mL two-necked round bottom flask, equipped with a reflux condenser and a magnetic stirrer, was charged with **1**^13^ (350 mg, 0.917 mmol) and trifluorobenzene (2 mL, 22.109 mmol). This mixture was heated to 65°C until complete dissolution of **1**, then AlCl_3_ (489 mg, 3.667 mmol) was added. The reaction mixture was stirred for 1.25 h at 65°C, then it was cooled to room temperature, quenched with a 1 N HCl solution and extracted with CHCl_3_ (2×30 mL). The combined organic phases were washed with brine, dried over anhydrous Na_2_SO_4_ and the solvent was removed under vacuum. The crude product was purified by column chromatography (silica gel, hexane/chloroform 9: 1) to give **2** as a withe solid (510 mg, 73% yield). ^1^H-NMR (500 MHz, CDCl_3_), δ (ppm): 6.63 (t, *J* = 8.7 Hz, 8H), 6.58 (s, 2H); ^19^F-NMR (470 MHz, CDCl_3_), δ (ppm): -108.55 (quintet, *J* = 7.8 Hz, 4F), -108.73 (t, *J* = 7.8 Hz, 8F); ^13^C-NMR (126 MHz, CDCl_3_), δ (ppm): 162.3 (dt, *J* = 248.8, 16.4 Hz), 161.8 (ddd, *J* = 251.3, 15.5 10.2 Hz), 137.1, 134.5, 111.4 (td, *J* = 15.8, 4.8 Hz), 100.9-100.4 (m), 36.8.

***2,2',2'',2'''-((Perchlorocyclohexa-2,5-diene-1,4-diylidene)bis(methanediylylidene))tetrakis(1,3,5-trifluorobenzene)*** **F,Cl-TTH**.

A 50 mL two-necked round bottom flask, equipped with a magnetic stirrer, was charged, under nitrogen atmosphere, with a solution of **2** (250 mg, 0.327 mmol) in anhydrous THF (40 mL), then a 55% w/w aqueous solution of tetrabutylammonium hydroxide (340 μL, 0.722 mmol) was added. The resulting mixture was stirred for 3.5 h at room temperature in the dark (after 2-2.5 h the solution turns blue), then tetrachloro-1,4-benzoquinone (186 mg, 0.756 mmol) was added. After stirring overnight, the reaction solvent was removed at reduced pression and the crude product was purified by column chromatography (silica gel, hexane/chloroform 8: 2) to give **F,Cl-TTH** as a white solid (142 mg, 57% yield). ^1^H-NMR (500 MHz, CDCl_3_), δ (ppm): 6.67 (br s, 8H); ^1^H-NMR (500 MHz, THF-d_8_), δ (ppm): 6.95 (br s, 4H), 6.93 (br s, 4H); ^19^F-NMR (470 MHz, CDCl_3_), δ (ppm): -105.82 (br s, 12F); ^19^F-NMR (470 MHz, THF-d_8_), δ (ppm): -104.75 (br s, 8F), -104.96 (br s, 4H); ^13^C-NMR (126 MHz, CDCl_3_), δ (ppm): 163.6 (br d, *J* = 248.9 Hz), 160.5 (br dd, *J* = 255.0, 16.2 Hz), 136.9, 130.2, 123.1, 112.6 (br s), 100.6 (br t, *J* = 21.1 Hz); HRMS (LC-IT-TOF, elution with methanol) m/z: M^-^ calculated for C_32_H_8_Cl_4_F_12_ 759.9194; found 759.9178.

***2,2',2'',2'''-((Perfluoro-1,4-phenylene)bis(methanetriyl))tetrakis(1,3,5-trichlorobenzene)* 4**.

A 50 mL two-necked round bottom flask, equipped with a reflux condenser and a magnetic stirrer, was charged with **3**^14^ (500 mg, 1.583 mmol) and trichlorobenzene (5.00 g, 27.557 mmol). This mixture was heated to 80°C until complete fusion of the reagents, then AlCl_3_ (843 mg, 6.332 mmol) was added. The reaction mixture was stirred for 1.5 h at 80°C, then it was cooled to room temperature, quenched with a 1 N HCl solution and extracted with CHCl_3_ (2×30 mL). The combined organic phases were washed with brine, dried over anhydrous Na_2_SO_4_ and the solvent was removed under vacuum. The crude product was purified by column chromatography (silica gel, hexane) and, after crystallization from hexane, compound **4** was obtained as a white solid (1.333 g, 94% yield). ^1^H-NMR (500 MHz, CDCl_3_), δ (ppm): 7.32 (br s), 6.62 (br s), 6.60 (br s) (signal integration ratio = 8:1:1); ^19^F-NMR (470 MHz, CDCl_3_), δ (ppm): -140.79 (br s), -141.43 (d, *J* = 22.6 Hz), -142.11 (d, *J* = 21.7 Hz), -142.78 (d, *J* = 5.4 Hz) (signal integration ratio = 1:1:1:1). The observed NMR signal pattern is consistent with the presence of atropisomers in a 1:1 ratio, arising from restricted rotation.^13^

***2,2',2'',2'''-((Perfluorocyclohexa-2,5-diene-1,4-diylidene)bis(methanediylylidene))tetrakis(1,3,5-trichlorobenzene)* Cl,F-TTH**.

A 100 mL two-necked round bottom flask, equipped with a magnetic stirrer, was charged, under nitrogen atmosphere, with a solution of **4** (500 mg, 0.558 mmol) in anhydrous THF (50 mL), then a 55% w/w aqueous solution of tetrabutylammonium hydroxide (580 μL, 1.228 mmol) was added dropwise. The resulting mixture was stirred for 4 h at room temperature in the dark (after 0.5 h the solution turns blue), then tetrachloro-1,4-benzoquinone (316 mg, 1.283 mmol) was added. After stirring overnight, the reaction solvent was removed at reduced pression and the crude product was purified by column chromatography (silica gel, hexane/chloroform 8.5:1.5). Crystallization from hexane gave compound **Cl,F-TTH** as an orange solid (349 mg, 70% yield). ^1^H-NMR (500 MHz, C_2_D_2_Cl_4_), δ (ppm): 7.41 (s, 4H), 7.31 (s, 4H); ^1^H-NMR (500 MHz, THF-d_8_), δ (ppm): 7.61 (br s, 4H), 7.53 (br s, 4H); ^19^F-NMR (470 MHz, C_2_D_2_Cl_4_), δ (ppm): -139.55 (br s, 4F); ^19^F-NMR (470 MHz, THF-d_8_), δ (ppm): -140.43 (br s, 4F); ^13^C-NMR (126 MHz, C_2_D_2_Cl_4_), δ (ppm): 142.4 (br dd, *J* = 259.1, 15.0 Hz), 138.8 (br s), 135.3, 133.9, 129.0 (br s), 127.9 (br s), 124.9 (br s); HRMS (LC-IT-TOF, elution with methanol) m/z: M^-^ calculated for C_32_H_8_Cl_12_F_4_ 887.6830; found 887.6801.

**Figure S1**. ^1^H NMR and ^19^F NMR spectra of compound **2** (500 and 470 MHz, CDCl_3_).

**Figure S2**. ^13^C NMR spectrum of compound **2** (126 MHz, CDCl_3_).

**Figure S3**. ^1^H NMR and ^19^F NMR spectra of **F,Cl-TTH** (500 and 470 MHz, CDCl_3_).

**Figure S4**. ^1^H NMR and ^19^F NMR spectra of **F,Cl-TTH** (500 and 470 MHz, THF-d_8_).

**Figure S5**. ^13^C NMR spectrum of **F,Cl-TTH** (126 MHz, CDCl_3_).

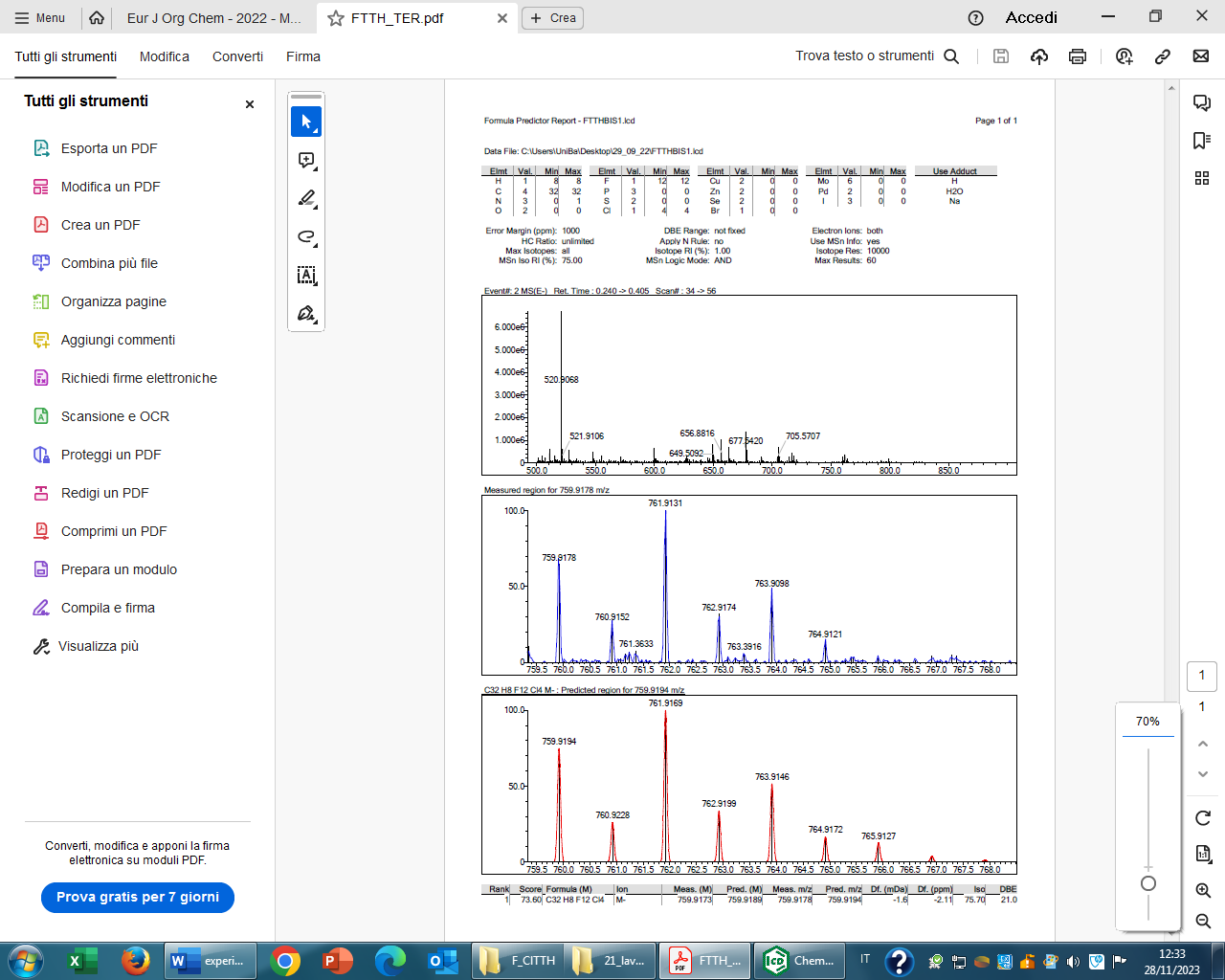


**Figure S6**. High resolution mass spectrum of **F,Cl-TTH**.

**Figure S7**. ^1^H NMR and ^19^F NMR spectra of compound **4** (500 and 470 MHz, CDCl_3_).

**Figure S8**. ^1^H NMR and ^19^F NMR spectra of **Cl,F-TTH** (500 and 470 MHz, C_2_D_2_Cl_4_).

**Figure S9**. ^1^H NMR and ^19^F NMR spectra of **Cl,F-TTH** (500 and 470 MHz, THF-d_8_).

C_2_D_2_Cl_4_

298 °K

303 °K

308 °K

313 °K

323 °K

333 °K

343 °K

**Figure S10**. ^1^H NMR spectra of **Cl,F-TTH** (500 MHz, C_2_D_2_Cl_4_) at different temperatures (bottom up: 25, 30, 35, 40, 50, 60, 70 °C).

**Figure S11.** ^13^C NMR spectrum of Cl,F-TTH (126 MHz, C_2_D_2_Cl_4_).

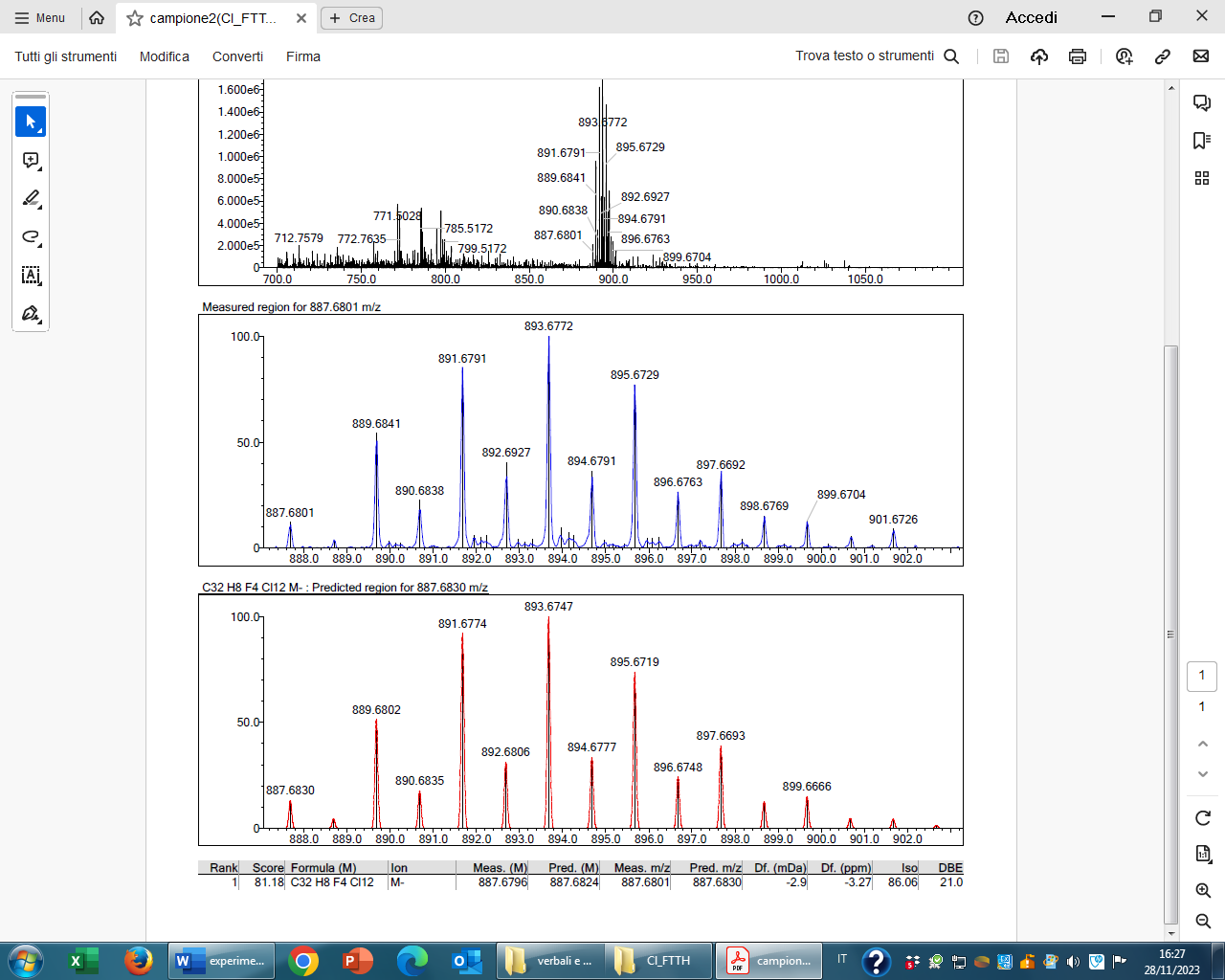


**Figure S12**. High resolution mass spectrum of **Cl,F-TTH**.

# Electrochemical and Thermal Characterization

**
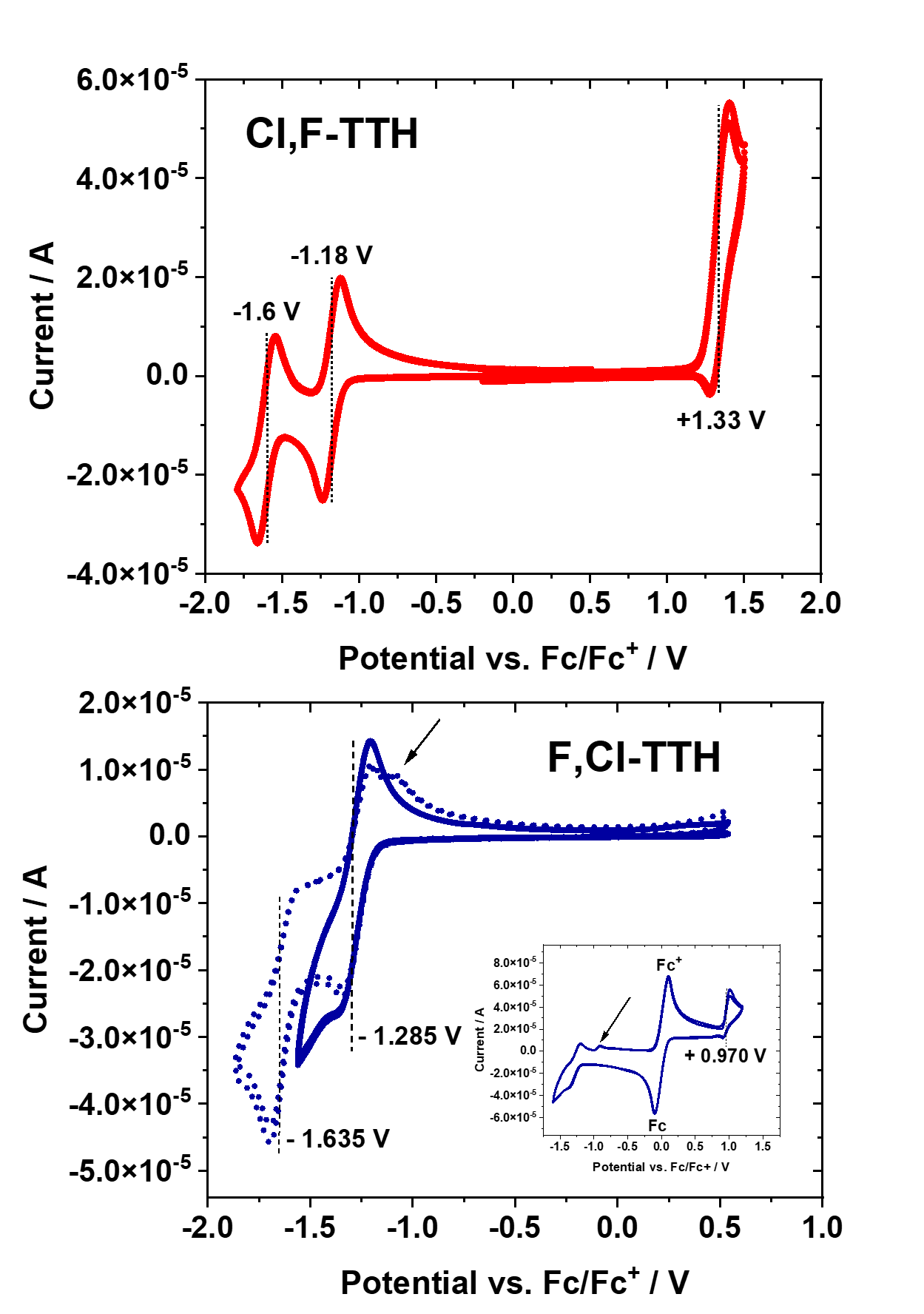
**

**Figure S13:** Cyclic voltammogram of a 0.5 mM solution of **Cl,F-TTH** (top) and **F,Cl-TTH** (bottom) with 0.1 M N(Bu)_4_PF_6_ in CH_2_Cl_2_ at the scan rate of 0.1 V/s. Inset: Cyclic voltammogram of a 0.5 mM solution of **F,Cl-TTH** with 1mM of Ferrocene in 0.1 M N(Bu)_4_PF_6_ in CH_2_Cl_2_ at the scan rate of 0.1 V/s.

**Figure S14:**  TGA spectra of **Cl,F-TTH** (red line) and **F,Cl-TTH** (blue line).

**Spectroscopic Characterization**


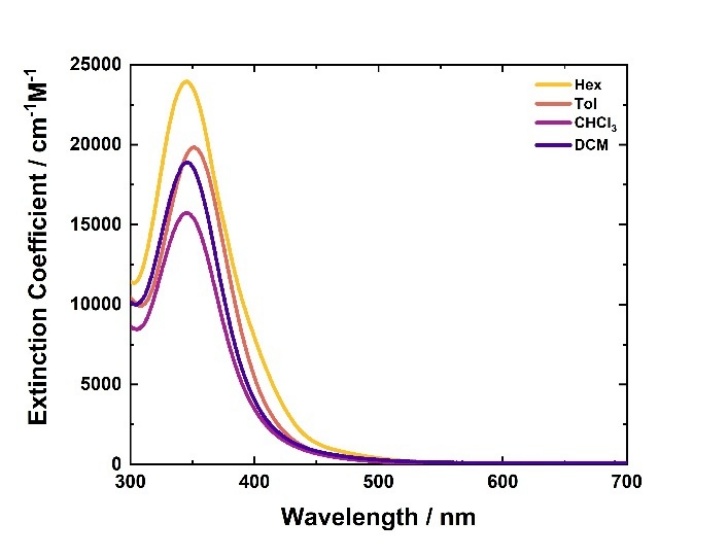

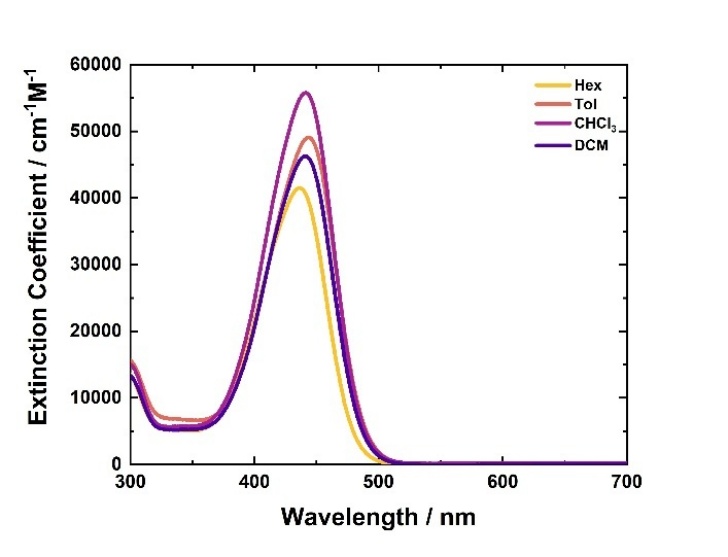


**Figure S15:** Molar extinction coefficient in different solvents – n-hexane (yellow), toluene (orange), chloroform (magenta) ,and dichloromethane (purple) – for **F,Cl-TTH** (left) and **Cl,F-TTH** (right).


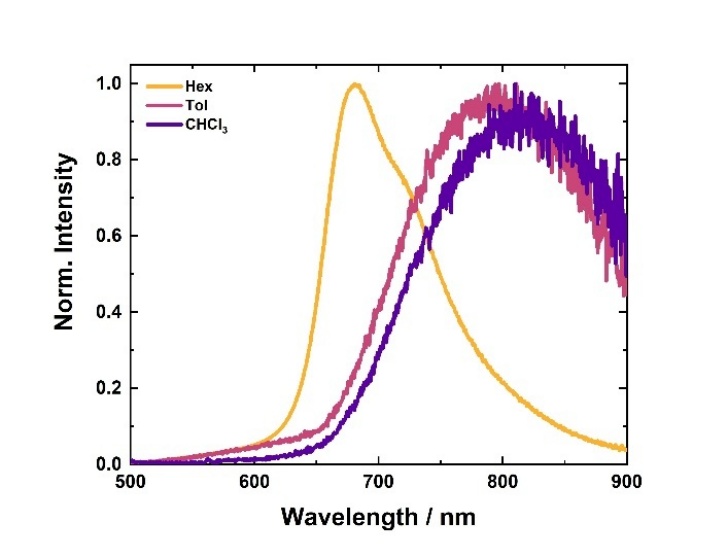

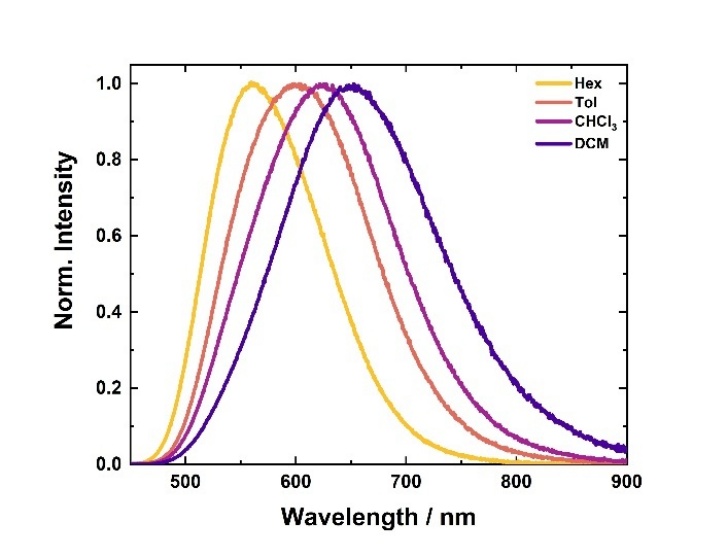


**Figure S16:** Normalized emission spectra in different solvents – n-hexane (yellow), toluene (orange), chloroform (magenta) ,and dichloromethane (purple) – for **F,Cl-TTH** (left) and **Cl,F-TTH** (right). Emission spectra were obtained at concentrations of 1 x 10^-5^ M upon excitation at central wavelength of 340 and 420 nm, respectively.


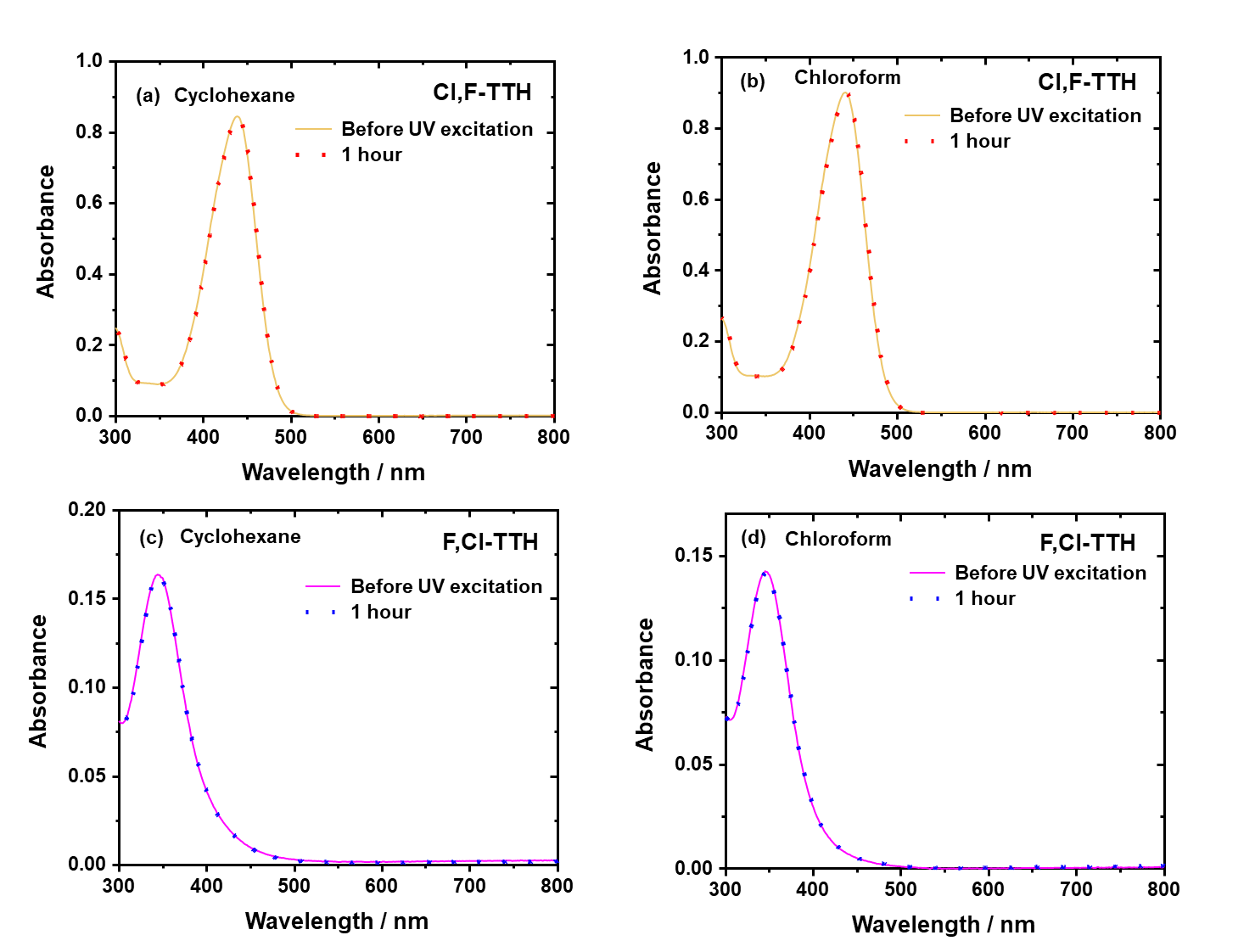


**Figure S17:** Absorption spectra before and after one hour of continuous irradiation with a UV LED lamp peaked at 365 nm (1W/cm^2^) of **Cl,F-TTH** in (a) cyclohexane, (b) chloroform and **F,Cl-TTH** in (c) cyclohexane, (d) chloroform. All solutions had similar optical density at the excitation wavelength.


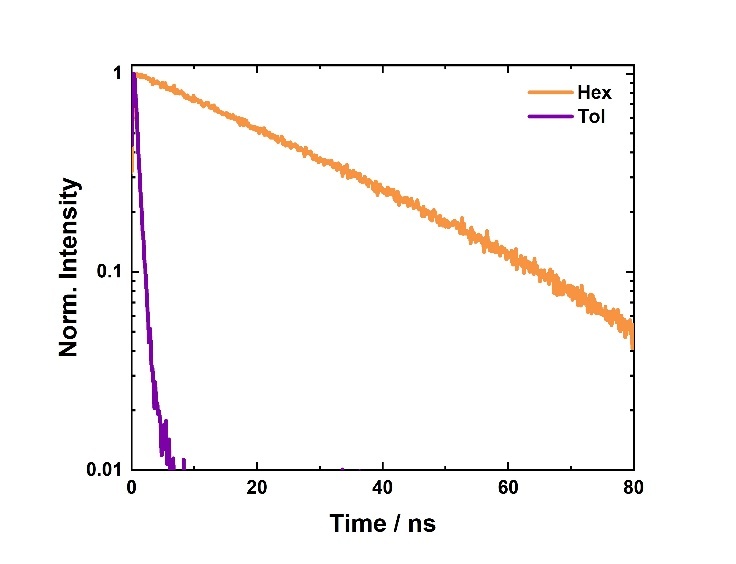

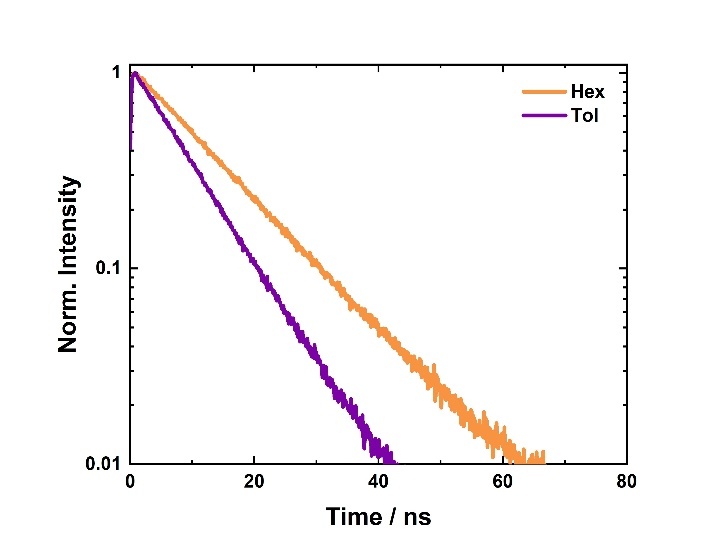


**Figure S18:** Normalized time-correlated single photon counting (TCSPC) decay profiles of **F,Cl-TTH** (left) and **Cl,F‑TTH** (right) in n-hexane and toluene. Kinetic emission profiles were obtained at concentrations of 1 x 10^-5^ M upon excitation at 355 and 420 nm, respectively

**Table S1:** Summary of the main optical properties of **Cl,F‑TTH** in different solvents.

| **Solvent** | **λ_Abs_ / nm** | **Ex. Coeff. / cm^-1^M^-1^** | **λ_Em_ / nm** | **PLQY** | **StS / eV** | **τ / ns** |
| --- | --- | --- | --- | --- | --- | --- |
| **Hex** | 436 | 41504 | 560 | 100% | 0.63 | 12.6 |
| **Tol** | 443 | 49104 | 583 | 74% | 0.67 | 8.4 |
| **CHCl_3_** | 441 | 55773 | 604 | 70% | 0.82 |  |
| **DCM** | 441 | 46278 | 652 | 58% | 0.97 |  |

**Table S2:** Summary of the main optical properties of **F,Cl‑TTH** in different solvents.

| **Solvent** | **λ_Abs_ / nm** | **Ex. Coeff. / cm^-1^M^-1^** | **λ_Em_ / nm** | **PLQY** | **StS / eV** | **τ / ns** |
| --- | --- | --- | --- | --- | --- | --- |
| **Hex** | 344 | 23964 | 681 | 65% | 1.78 | 24.9 |
| **Tol** | 352 | 19849 | 791 | 1.1% | 1.96 | ~0.6 |
| **CHCl_3_** | 346 | 15723 | 808 | <1% | 2.05 |  |
| **DCM** | 346 | 18889 | - | 0% | - |  |


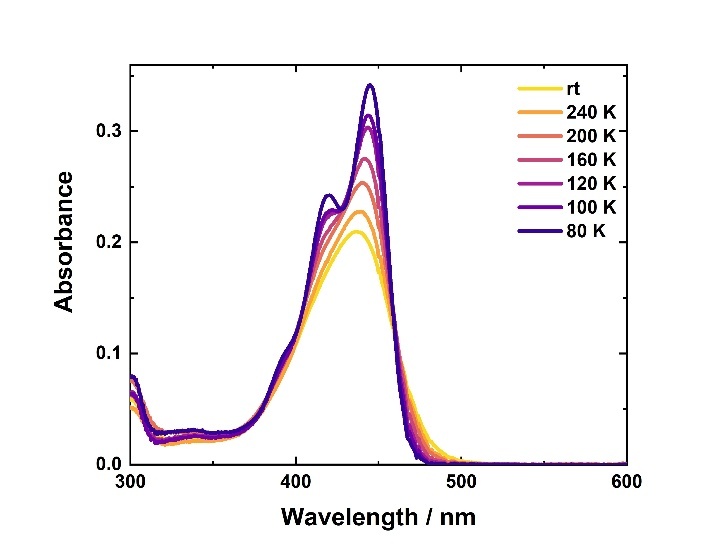

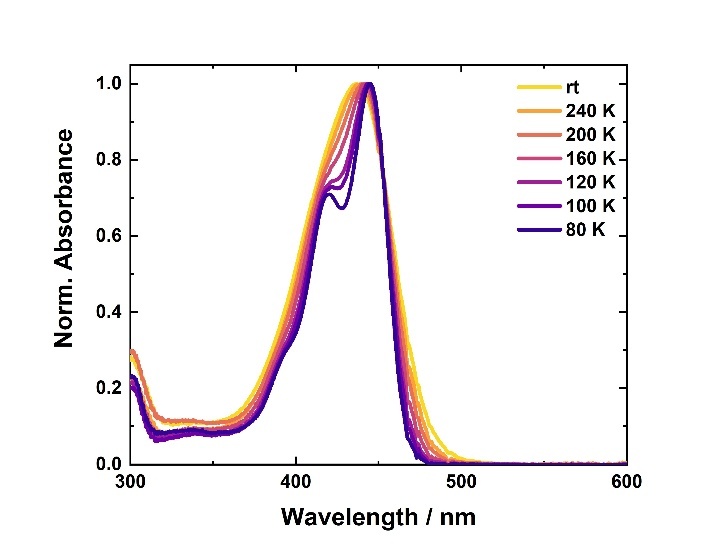


**Figure S19:** Temperature dependent absorption spectra of **Cl,F-TTH** in 3-methylpentane obtained at concentrations of 5x10^-6^ M (left) together with the associated normalized absorption profiles (right).


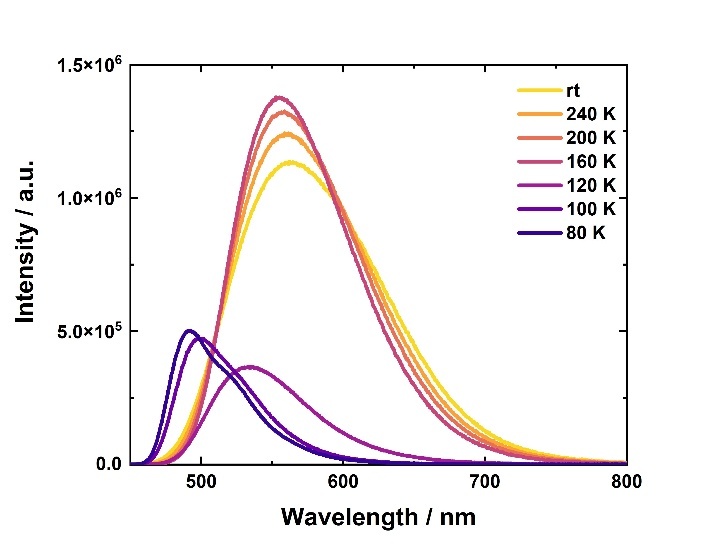

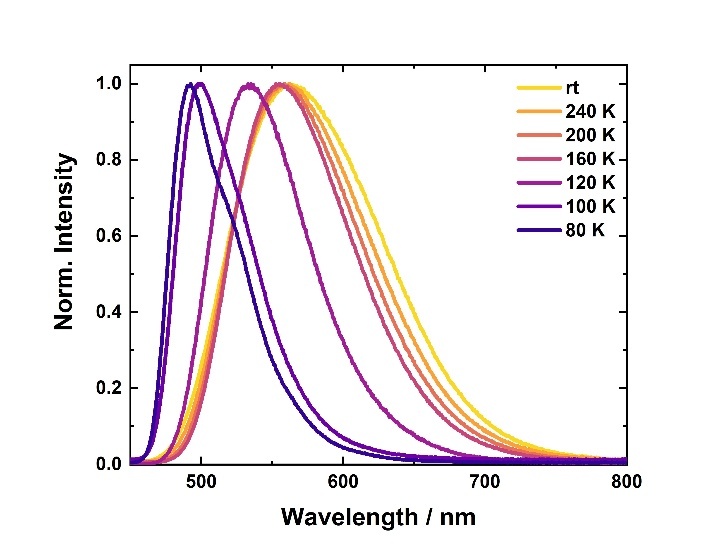


**Figure S20:** Temperature dependent emission spectra of **Cl,F-TTH** in 3-methylpentane obtained at concentrations of 5x10^-6^ M obtained at excitation centred at 420 nm (left) together with the normalized emission profiles (right).


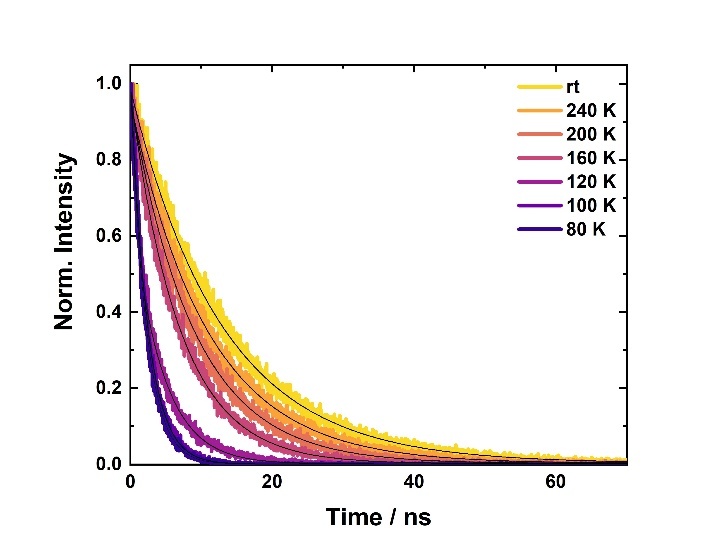

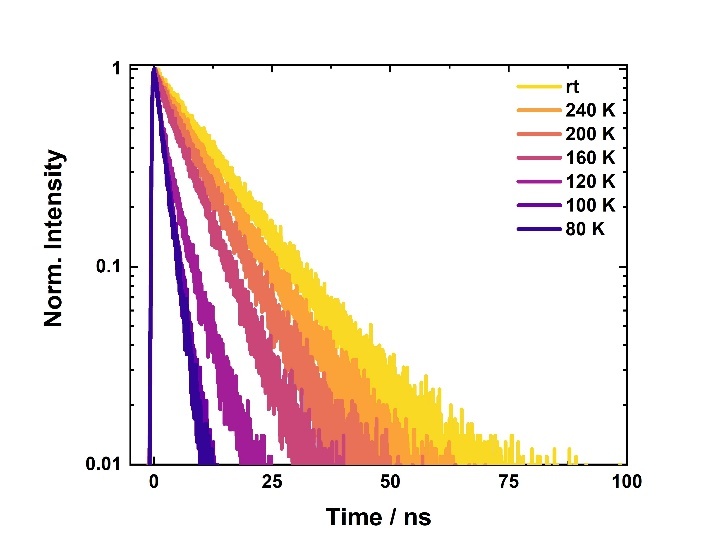


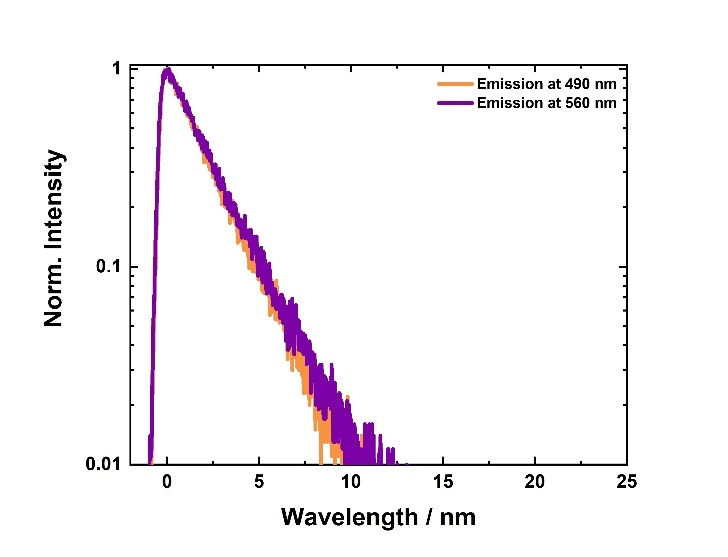


**Figure S21:** Temperature dependent time-correlated single photon counting (TCSPC) decay profiles of **Cl,F‑TTH** in 3-methylpentane shown in linear (top left) and logarithmic (top right) representation. Kinetic emission profiles were obtained at concentrations of 5x10^-6^ M upon excitation at 355 nm. Bottom graph shows decay profiles at different emission wavelengths at a temperature of 80 K.

**Table S3:** Summary of the main optical properties of **Cl,F‑TTH** in 3-methylpentane at various temperatures.

| **Temperature** | **λ_Abs_ / nm** | **λ_Em_ / nm** | **StS / eV** | **τ / ns** |
| --- | --- | --- | --- | --- |
| **rt** | 438 | 563 | 0.63 | 12.6 |
| **240 K** | 440 | 560 | 0.61 | 10.8 |
| **200 K** | 441 | 558 | 0.59 | 8.93 |
| **160 K** | 442 | 555 | 0.57 | 6.91 |
| **120 K** | 444 | 534 | 0.47 | 4.28 |
| **100 K** | 444 | 499 | 0.31 | 2.36 |
| **80 K** | 445 | 492 | 0.27 | 2.20 |


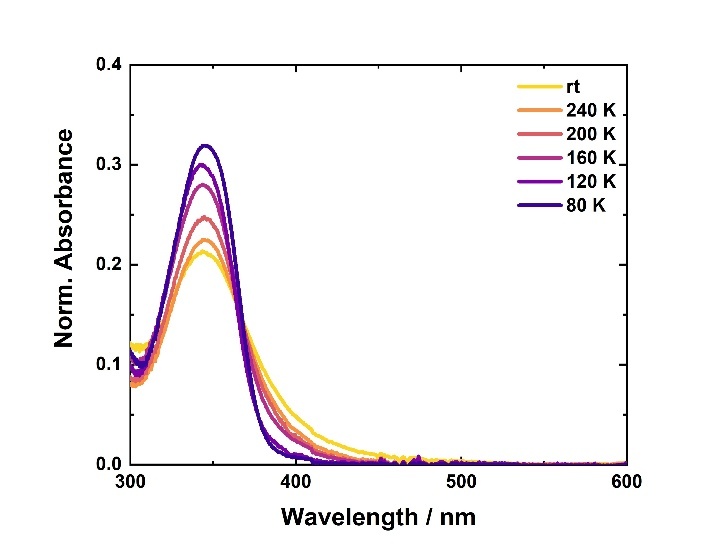

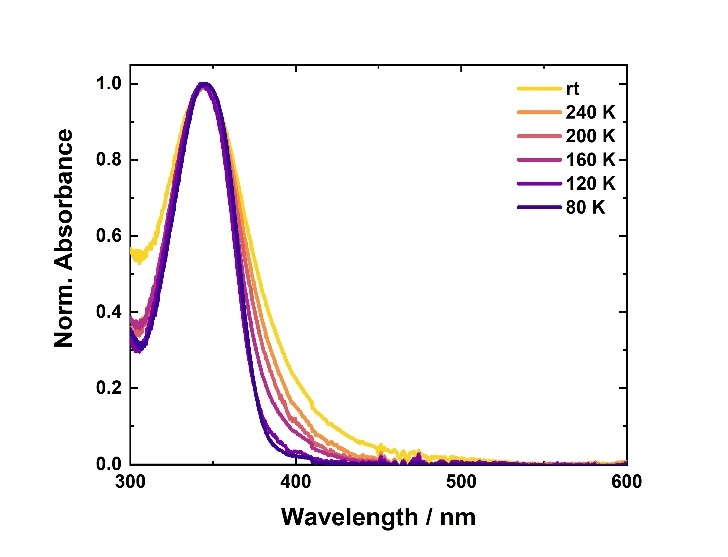


**Figure S22:** Temperature dependent absorption spectra of **F,Cl-TTH** in 3-methylpentane obtained at concentrations of 1x10^-5^ M (left) together with the associated normalized absorption profiles (right).


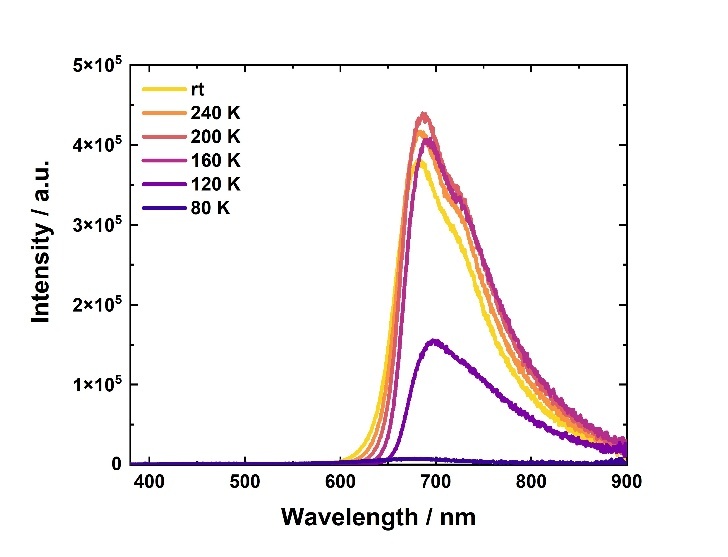

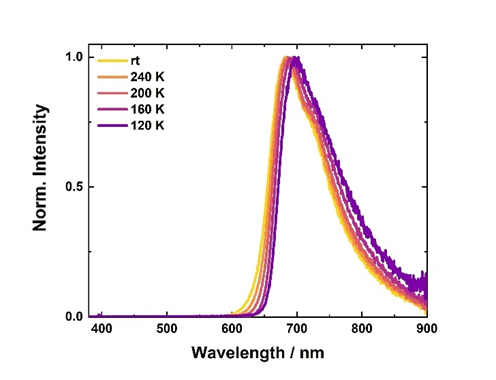


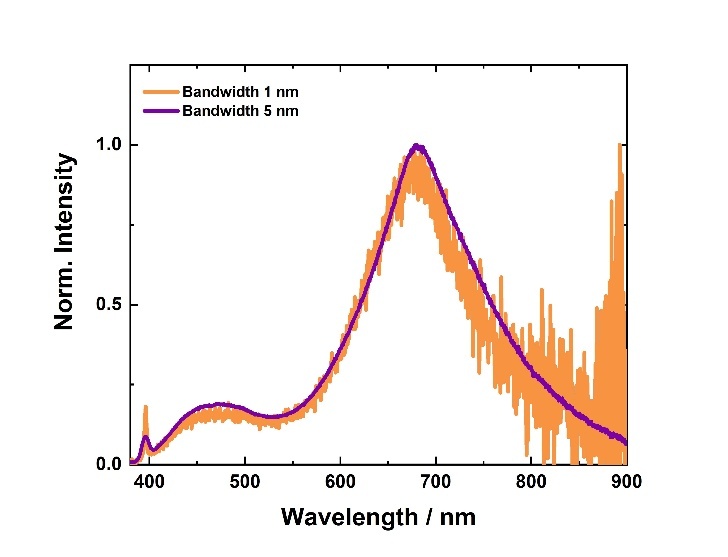


**Figure S23:** Temperature dependent emission spectra of **F,Cl-TTH** in 3-methylpentane obtained at concentrations of 5x10^-6^ M obtained at excitation centred at 355 nm (top left) together with the normalized emission profiles (top right). The bottom graph shows the normalized emission spectra at 80 K obtained at emission monochromator slit widths of 1 and 5 nm, respectively.


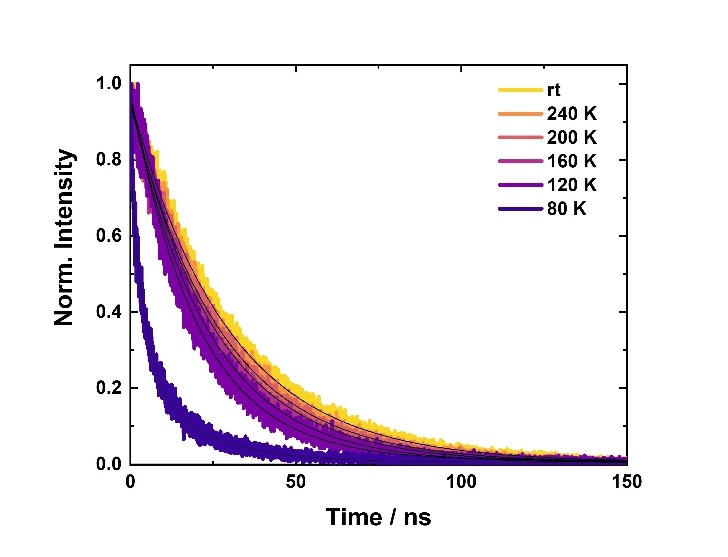

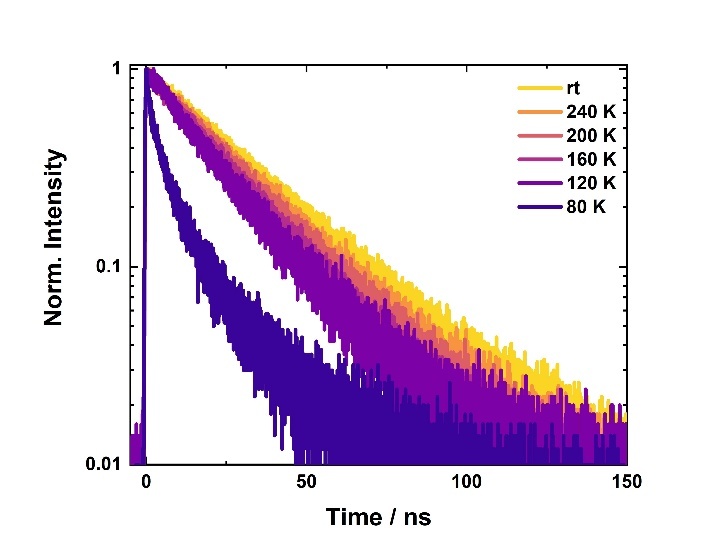


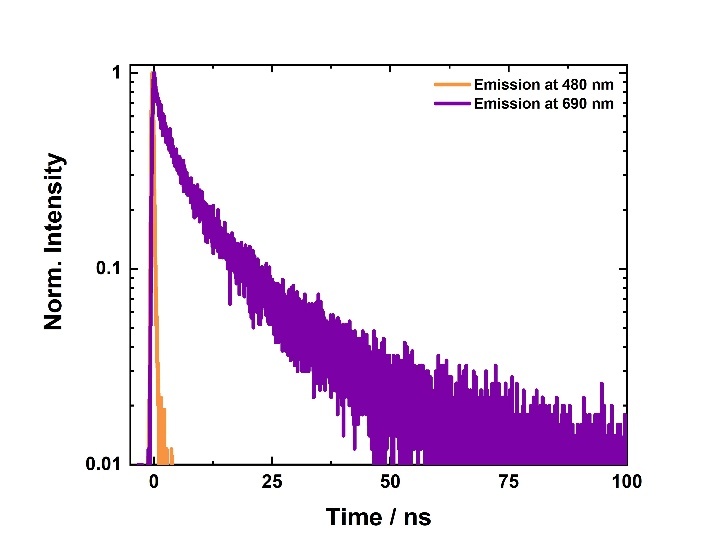


**Figure S24:** Temperature dependent time-correlated single photon counting (TCSPC) decay profiles of **F,Cl‑TTH** in 3-methylpentane shown in linear (top left) and logarithmic (top right) representation. Kinetic emission profiles were obtained at concentrations of 1x10^-5^ M upon excitation at 355 nm. Bottom graph shows decay profiles at different emission wavelengths at a temperature of 80 K.

**Table S4:** Summary of the main optical properties of **F,Cl‑TTH** in 3-methylpentane at various temperatures (fluorescence lifetimes obtained at emission wavelength of 690 nm).

| **Temperature** | **λ_Abs_ / nm** | **λ_Em_ / nm** | **StS / eV** | **τ / ns** |
| --- | --- | --- | --- | --- |
| **rt** | 343 | 683 | 1.80 | 29.5 |
| **240 K** | 344 | 685 | 1.80 | 27.2 |
| **200 K** | 344 | 687 | 1.80 | 25.5 |
| **160 K** | 344 | 692 | 1.81 | 23.3 |
| **120 K** | 343 | 699 | 1.84 | 20.4 |
| **80 K** | 346 | 680 | 1.76 | 3.8/18.1 |


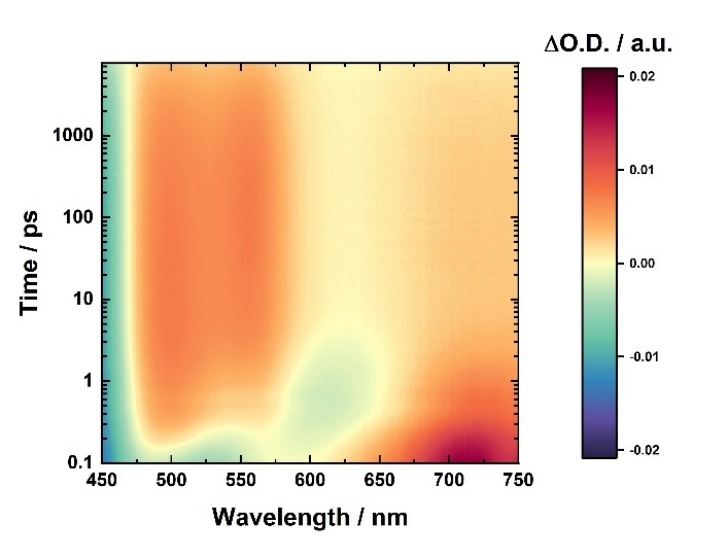

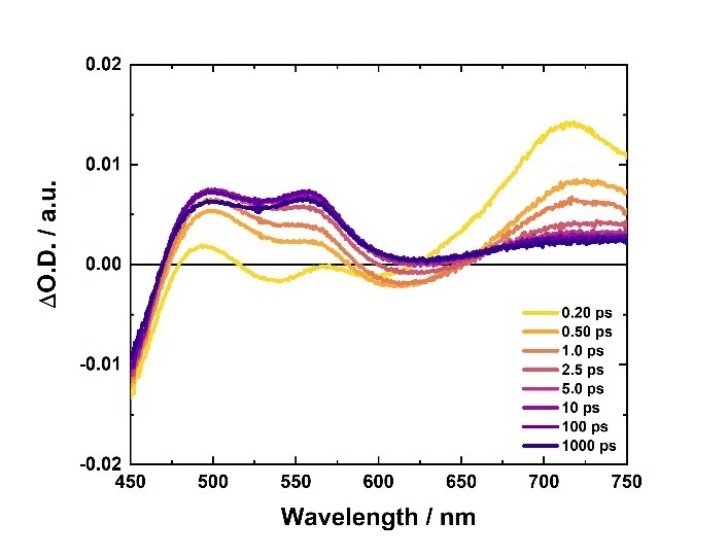


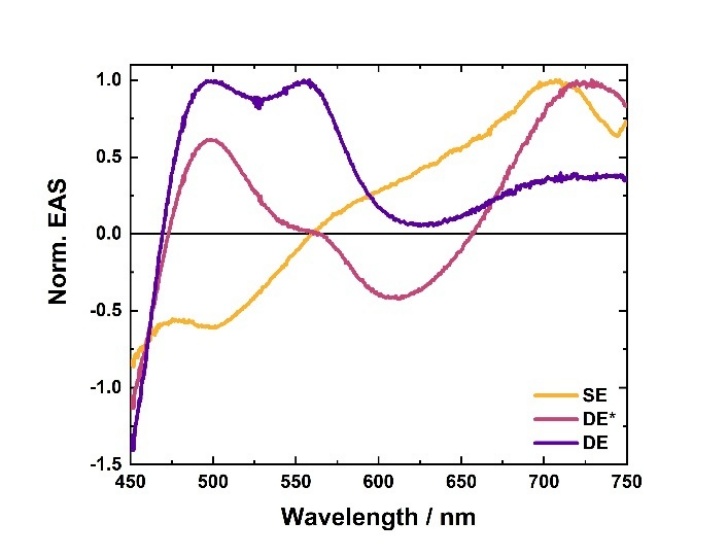


**Figure S25:** Top left: fs-TA contour plot of **Cl,F-TTH** in dichloromethane obtained upon photoexcitation at 440 nm. Top right: Corresponding differential absorption changes after different time delays illustrating the excited state dynamics. Bottom: Normalized EAS of the deconvoluted excited state species SE (yellow), unrelaxed DE* (magenta) ,and relaxed DE (purple) states obtained via global fitting of the fs-TA raw data.


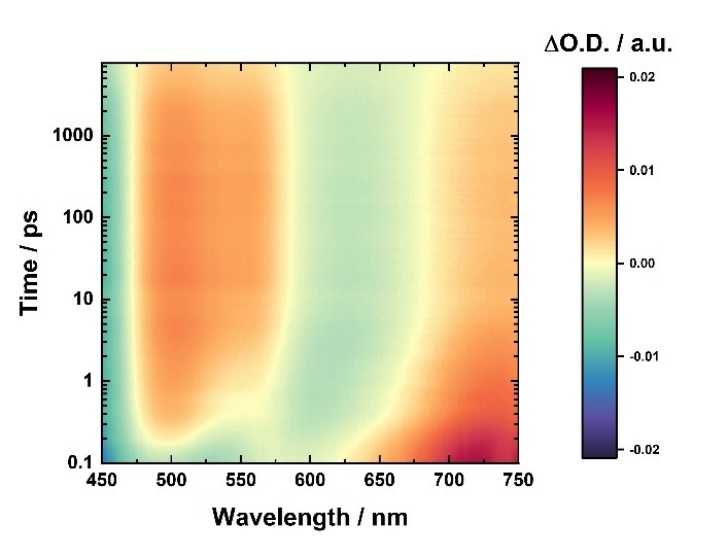

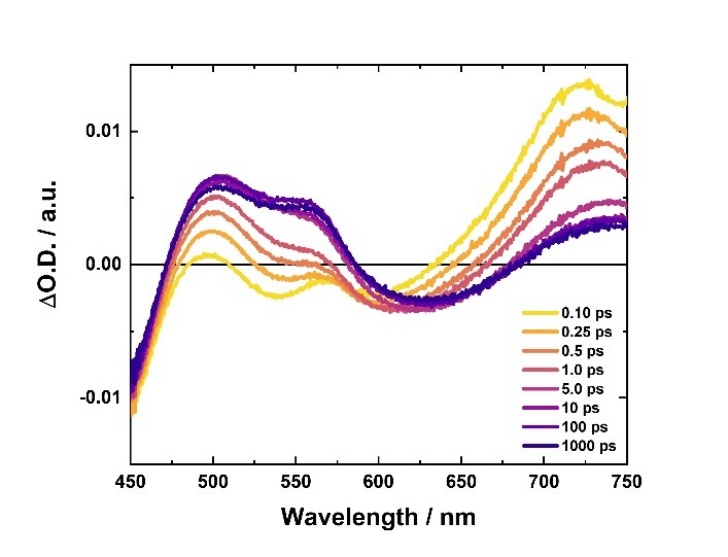


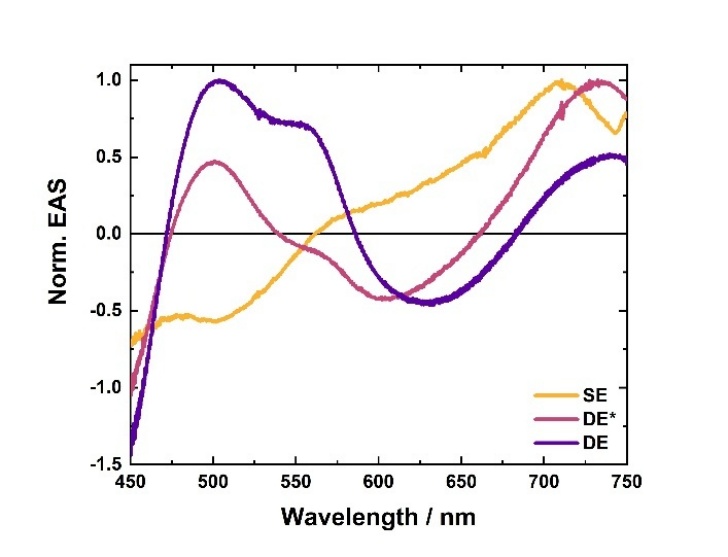


**Figure S26:** Top left: fs-TA contour plot of **Cl,F-TTH** in chloroform obtained upon photoexcitation at 440 nm. Top right: Corresponding differential absorption changes after different time delays illustrating the excited state dynamics. Bottom: Normalized EAS of the deconvoluted excited state species SE (yellow), unrelaxed DE* (magenta), and relaxed DE (purple) states obtained via global fitting of the fs-TA raw data.


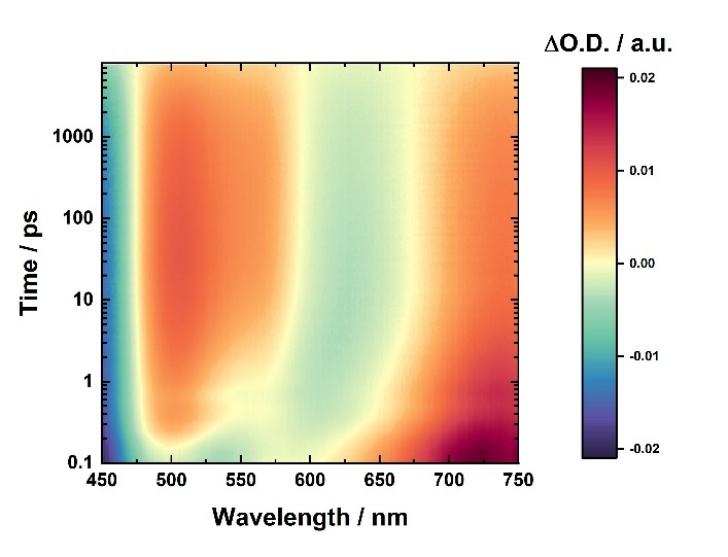

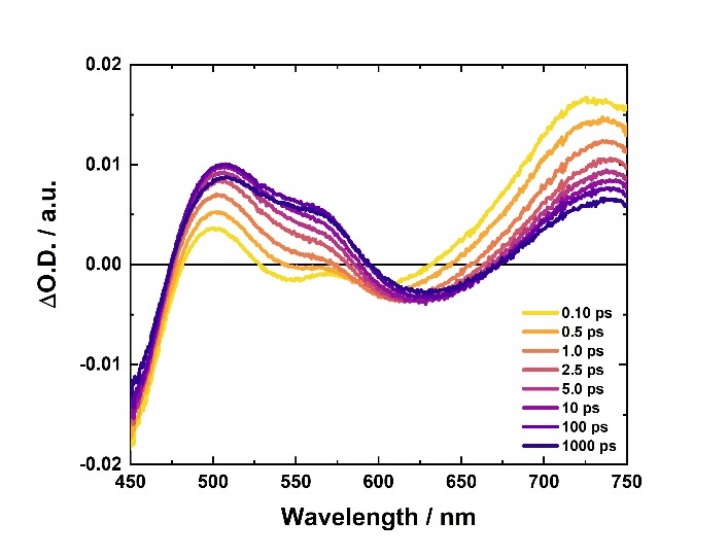


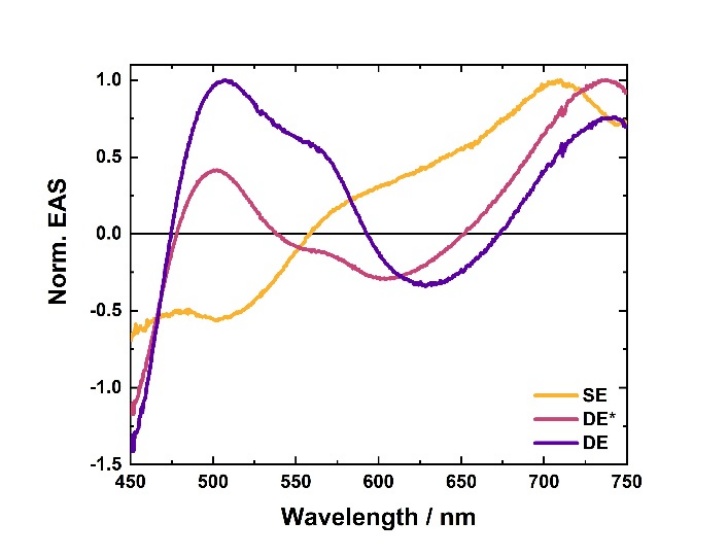


**Figure S27:** Top left: fs-TA contour plot of **Cl,F-TTH** in toluene obtained upon photoexcitation at 440 nm. Top right: Corresponding differential absorption changes after different time delays illustrating the excited state dynamics. Bottom: Normalized EAS of the deconvoluted excited state species SE (yellow), unrelaxed DE* (magenta), and relaxed DE (purple) states obtained via global fitting of the fs-TA raw data.


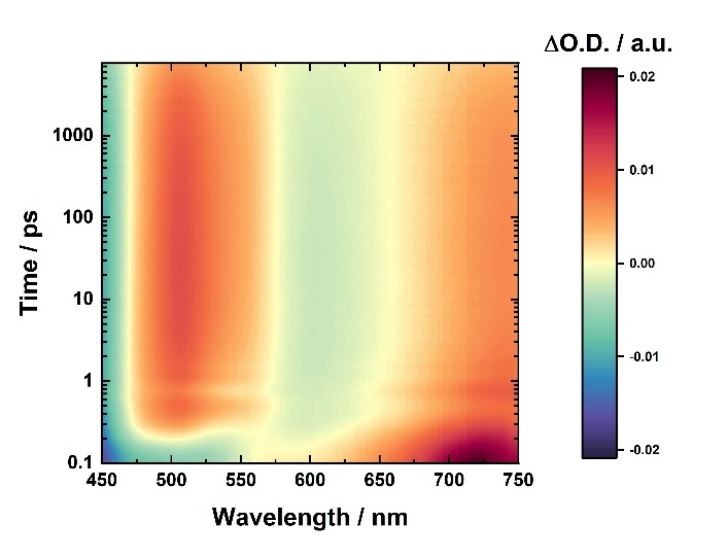

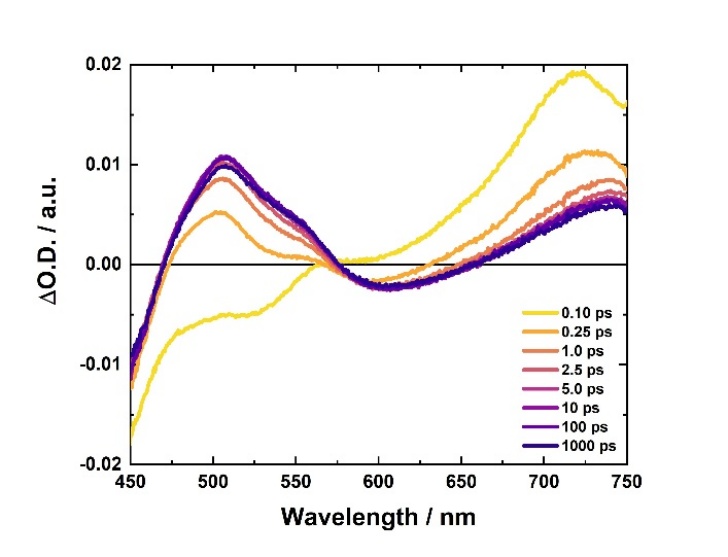


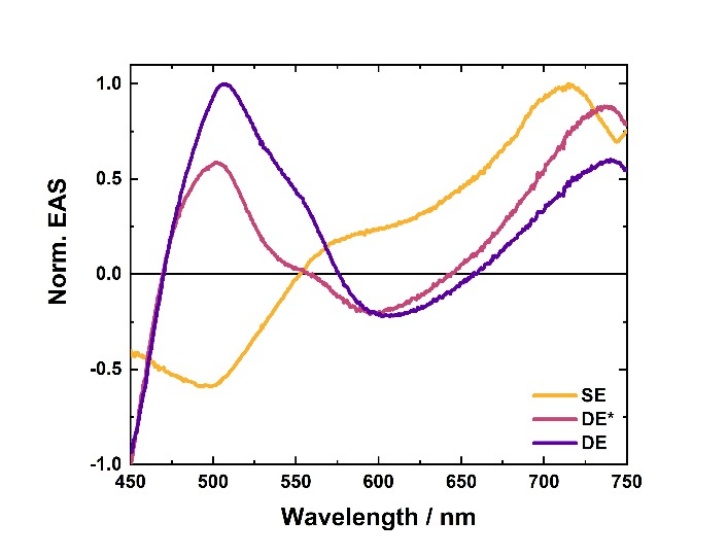


**Figure S28:** Top left: fs-TA contour plot of **Cl,F-TTH** in n-hexane obtained upon photoexcitation at 440 nm. Top right: Corresponding differential absorption changes after different time delays illustrating the excited state dynamics. Bottom: Normalized EAS of the deconvoluted excited state species SE (yellow), unrelaxed DE* (magenta) ,and relaxed DE (purple) states obtained via global fitting of the fs-TA raw data.


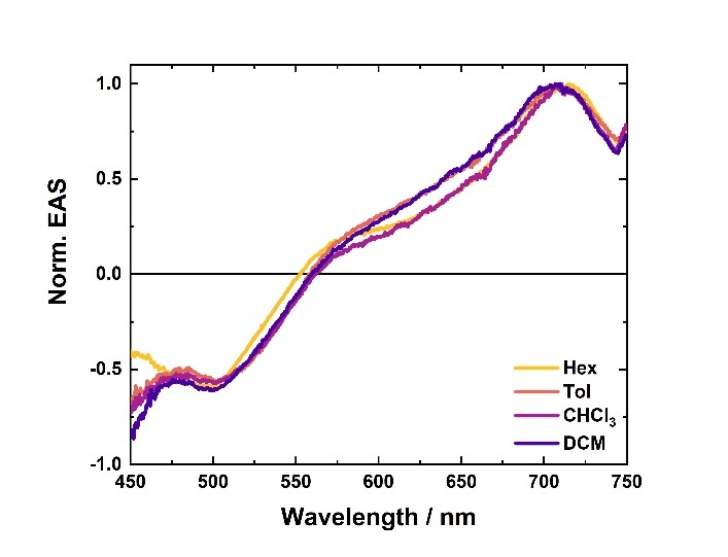

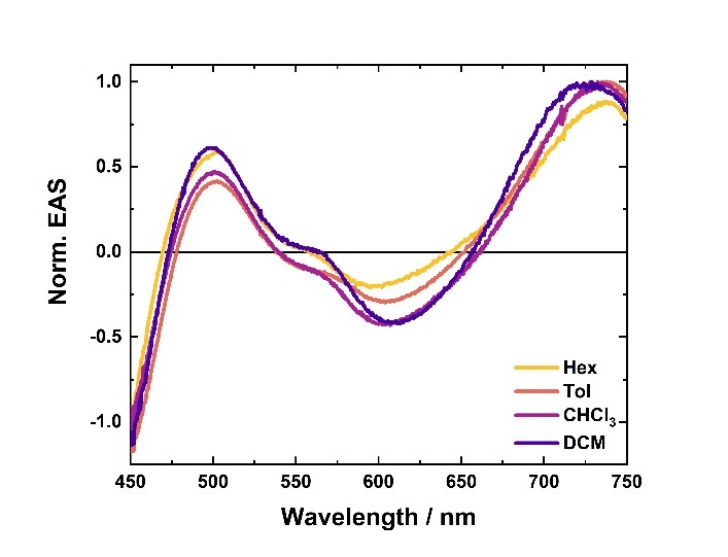


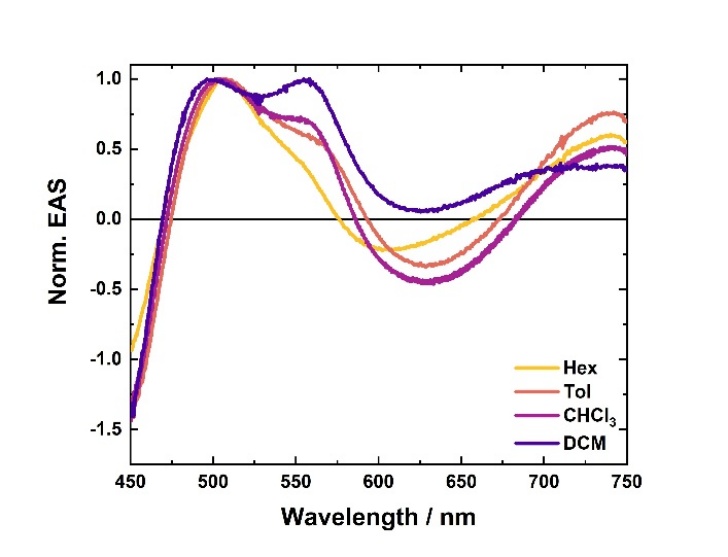


**Figure S29:** Normalized EAS of the deconvoluted species SE (top left), DE* (top right) ,and DE (bottom) for **Cl,F‑TTH** obtained *via* sequential global fitting of the corresponding fs-TA raw data measured in n-hexane (yellow), toluene (orange), chloroform (magenta), and dichloromethane (purple).


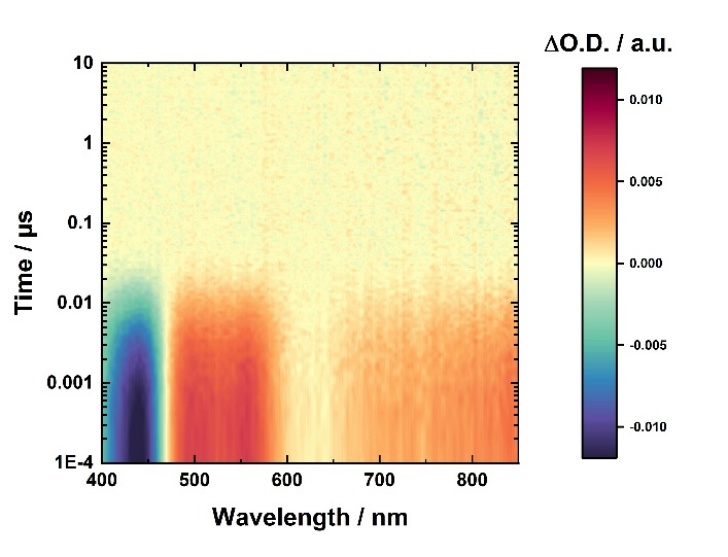

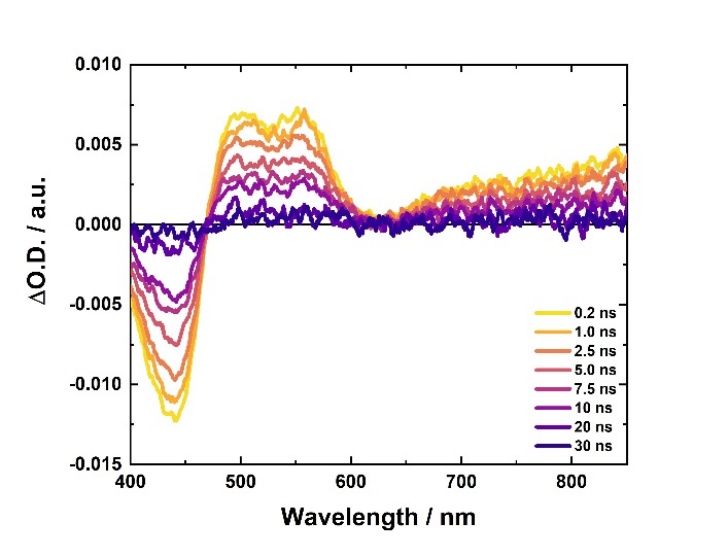


**Figure S30:** Left: ns-TA contour plot of **Cl,F-TTH** in dichloromethane obtained upon photoexcitation at 440 nm. Right: Corresponding differential absorption changes after different time delays illustrating the excited state dynamics.


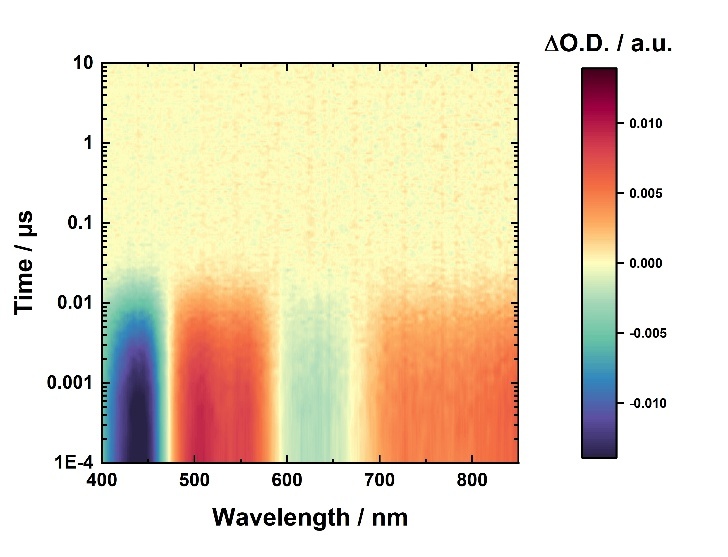

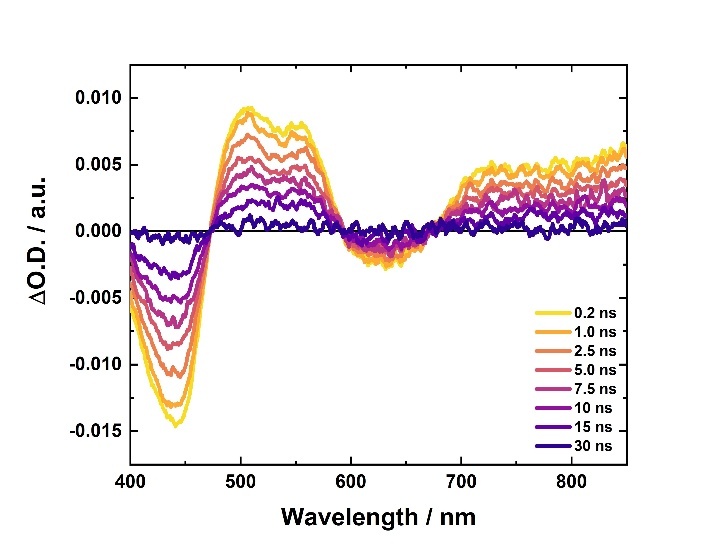


**Figure S31:** ns-TA contour plot of **Cl,F-TTH** in chloroform obtained upon photoexcitation at 440 nm. Right: Corresponding differential absorption changes after different time delays illustrating the excited state dynamics.


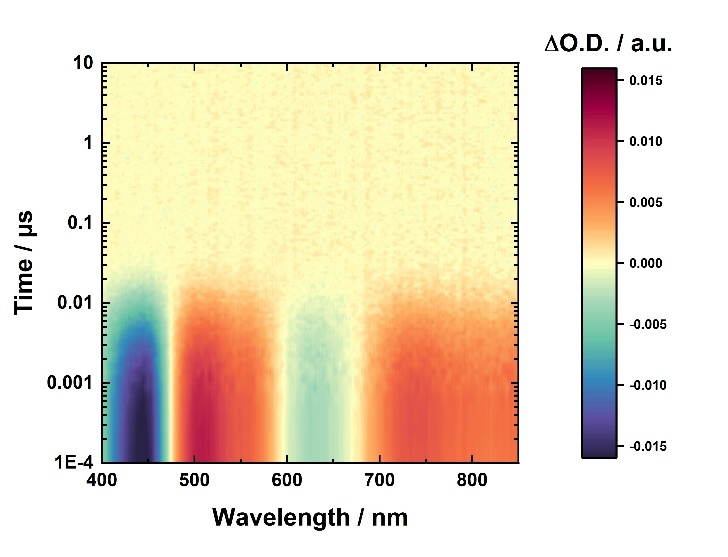

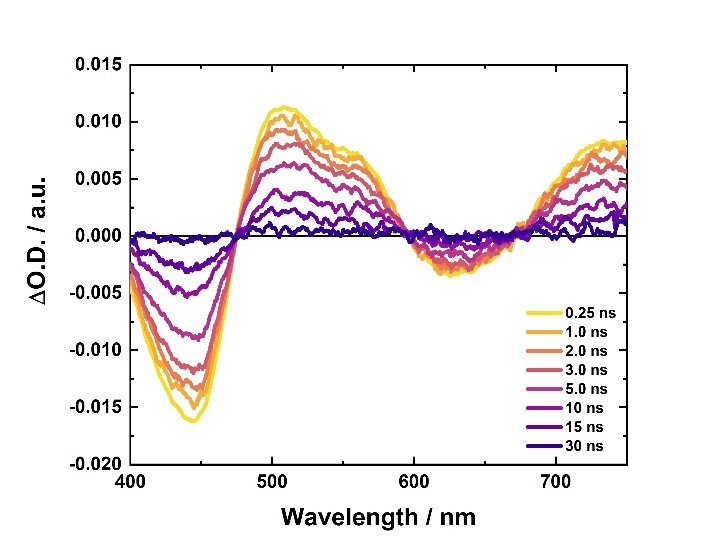


**Figure S32:** ns-TA contour plot of **Cl,F-TTH** in toluene obtained upon photoexcitation at 440 nm. Right: Corresponding differential absorption changes after different time delays illustrating the excited state dynamics.


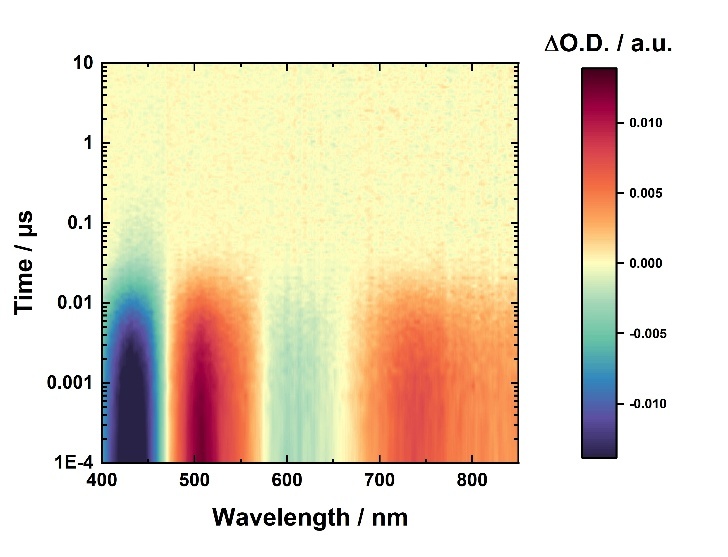

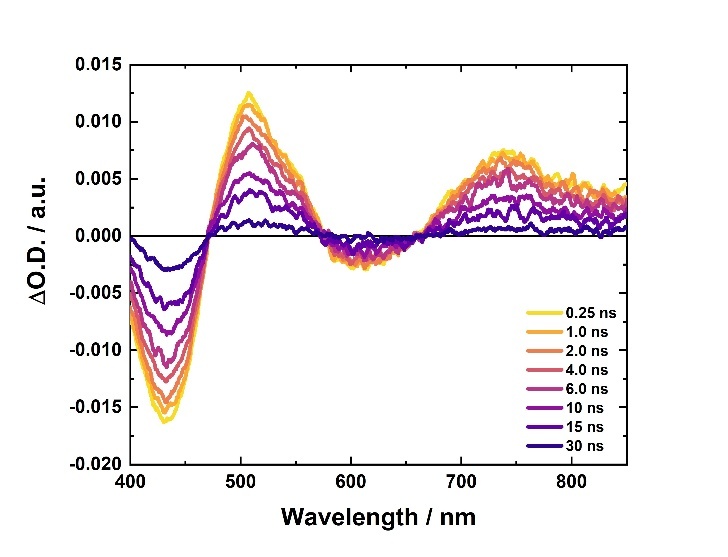


**Figure S33:** ns-TA contour plot of **Cl,F-TTH** in n-hexane obtained upon photoexcitation at 440 nm. Right: Corresponding differential absorption changes after different time delays illustrating the excited state dynamics.


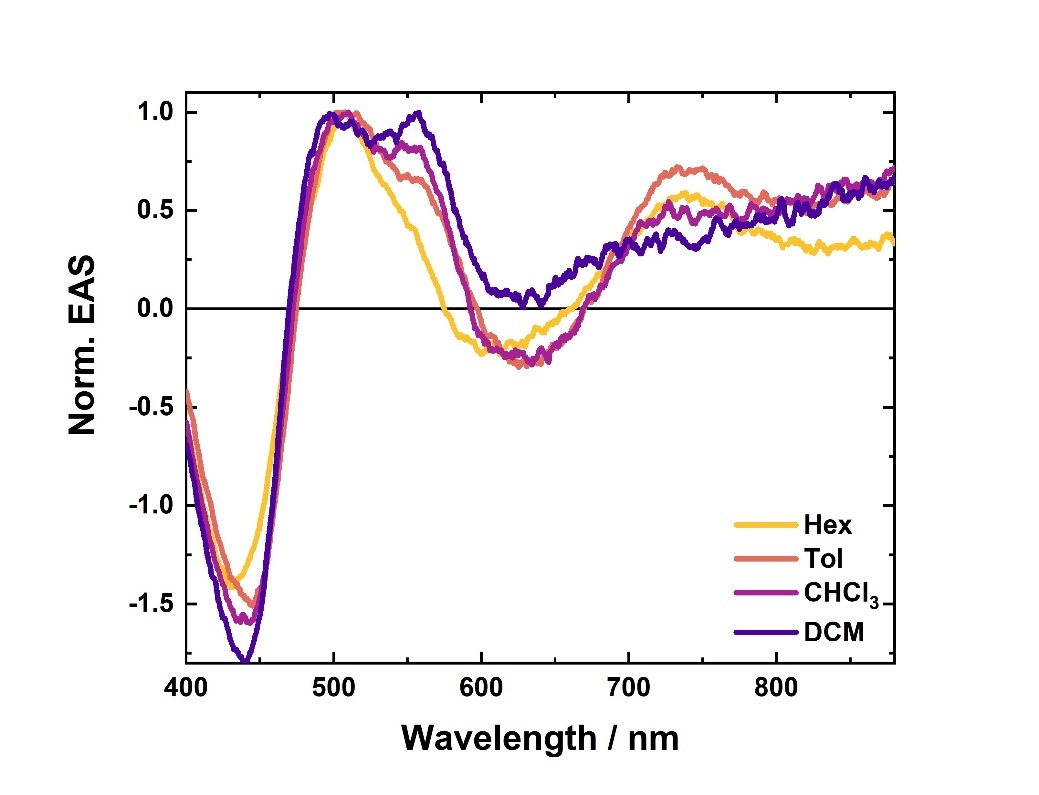


**Figure S34:** Normalized EAS of the deconvoluted species DE for **Cl,F‑TTH** obtained *via* sequential global fitting of the corresponding ns-TA raw data measured in n-hexane (yellow), toluene (orange), chloroform (magenta), and dichloromethane (purple).

**Table S5**: Summary of all excited state lifetimes for **Cl,F-TTH** in different solvents obtained *via* sequential global fitting of fs- and ns-TA raw data.

| **Solvent** | **τ(SE) / ps** | **τ(DE*) / ps** | **τ(DE) / ns** |
| --- | --- | --- | --- |
| **n-Hexane** | ~0.6 | 1.62 | 13.2 |
| **Toluene** | ~0.45 | 3.96 | 8.53 |
| **Trichloromethane** | ~0.45 | 2.72 | 9.81 |
| **Dichloromethane** | ~0.15 | 1.59 | 9.59 |


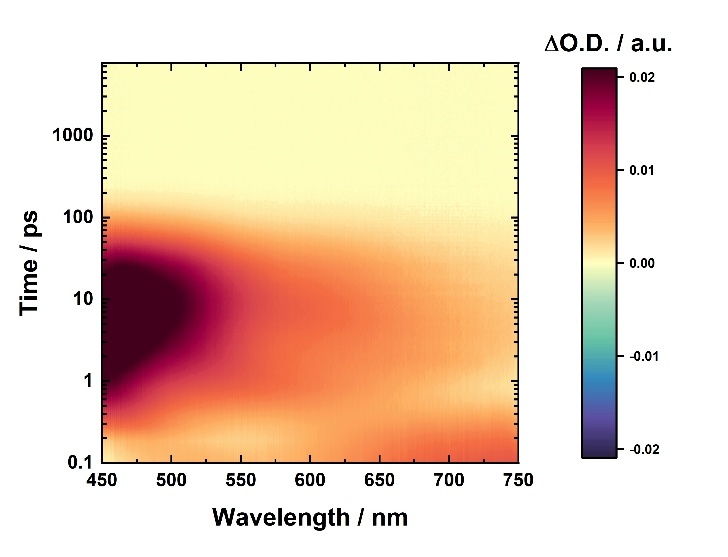

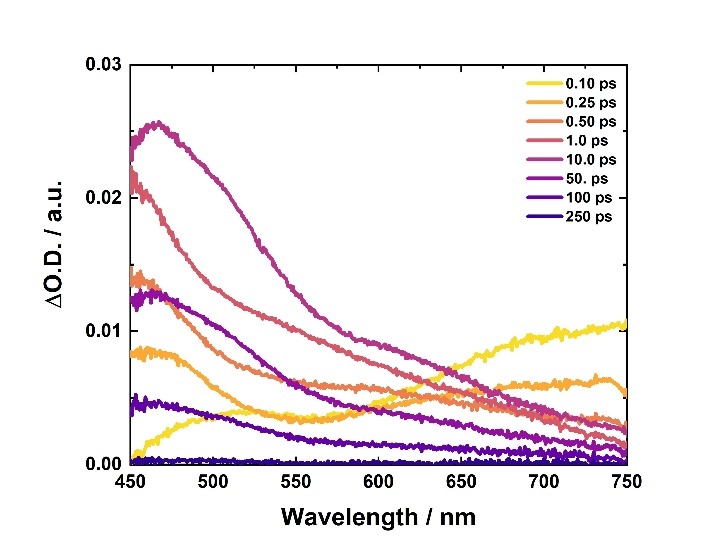


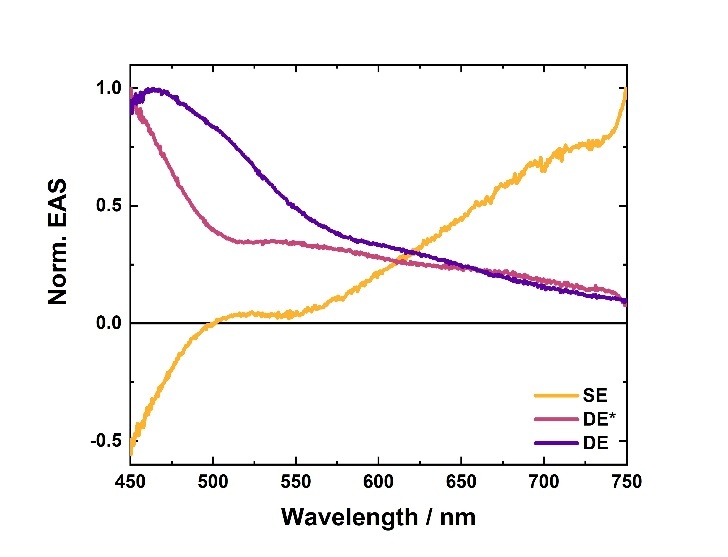


**Figure S35:** Top left: fs-TA contour plot of **F,Cl-TTH** in dichloromethane obtained upon photoexcitation at 360 nm. Top right: Corresponding differential absorption changes after different time delays illustrating the excited state dynamics. Bottom: Normalized EAS of the deconvoluted excited state species SE (yellow), unrelaxed DE* (magenta) ,and relaxed DE (purple) states obtained via global fitting of the fs-TA raw data.


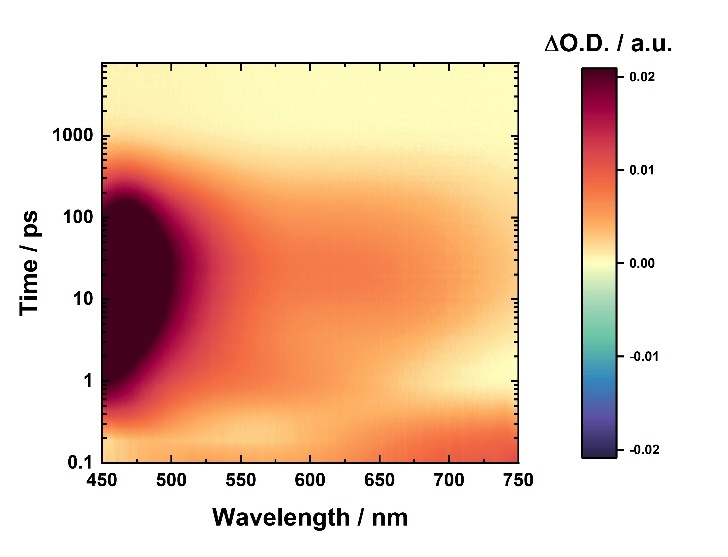

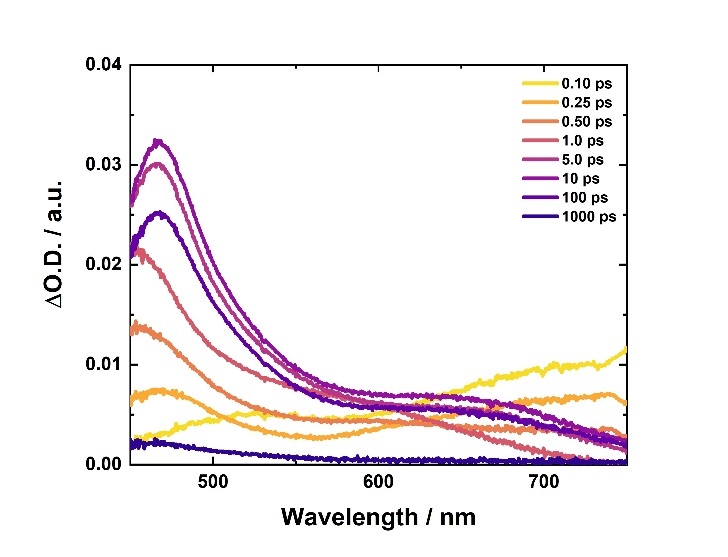


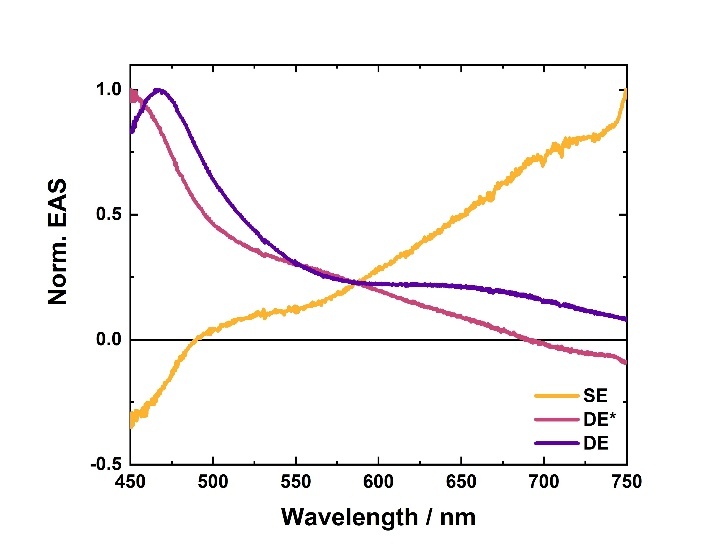


**Figure S36:** Top left: fs-TA contour plot of **F,Cl-TTH** in chloroform obtained upon photoexcitation at 360 nm. Top right: Corresponding differential absorption changes after different time delays illustrating the excited state dynamics. Bottom: Normalized EAS of the deconvoluted excited state species SE (yellow), unrelaxed DE* (magenta), and relaxed DE (purple) states obtained via global fitting of the fs-TA raw data.


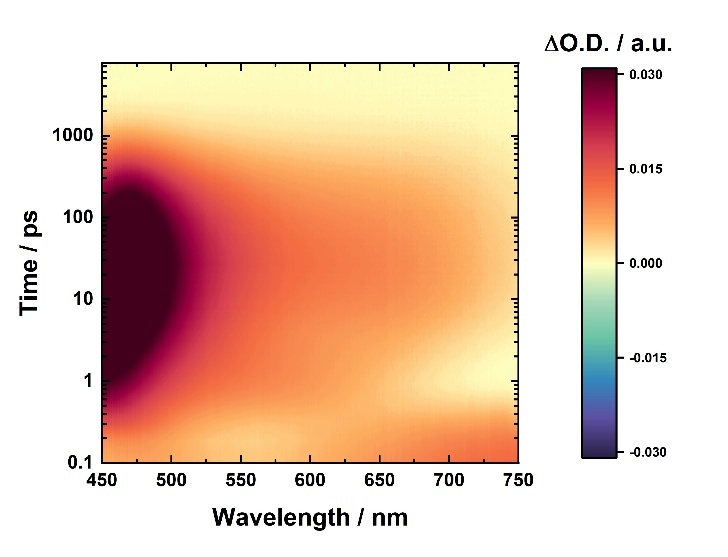

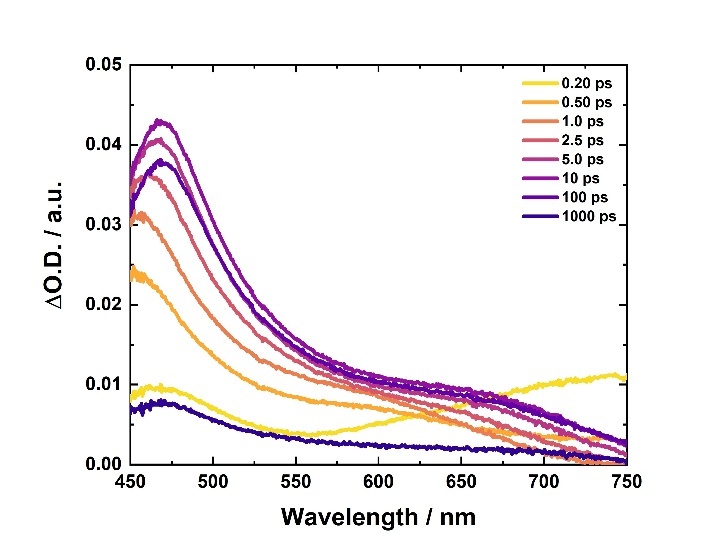


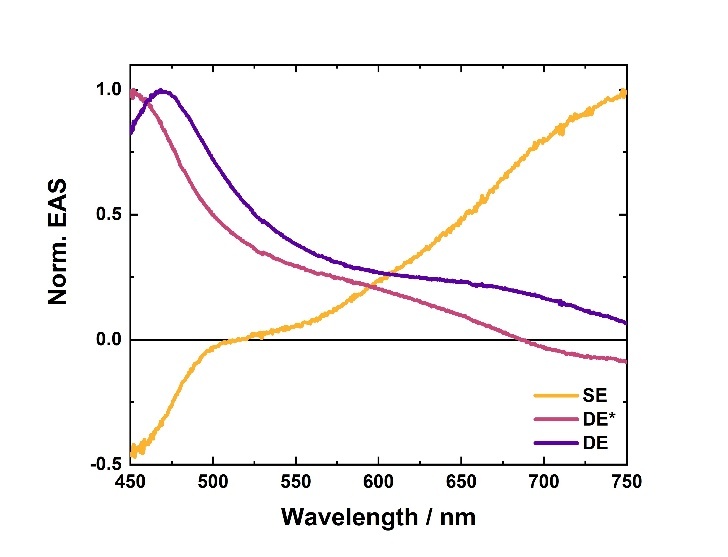


**Figure S37:** Top left: fs-TA contour plot of **F,Cl-TTH** in toluene obtained upon photoexcitation at 360 nm. Top right: Corresponding differential absorption changes after different time delays illustrating the excited state dynamics. Bottom: Normalized EAS of the deconvoluted excited state species SE (yellow), unrelaxed DE* (magenta) ,and relaxed DE (purple) states obtained via global fitting of the fs-TA raw data.


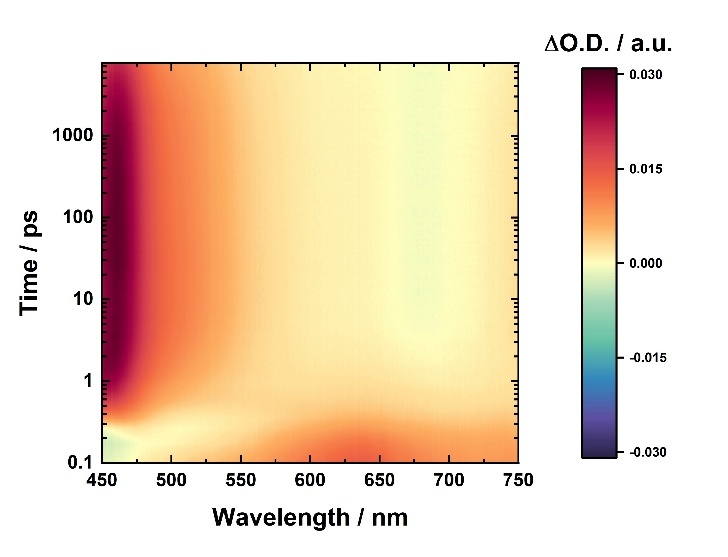

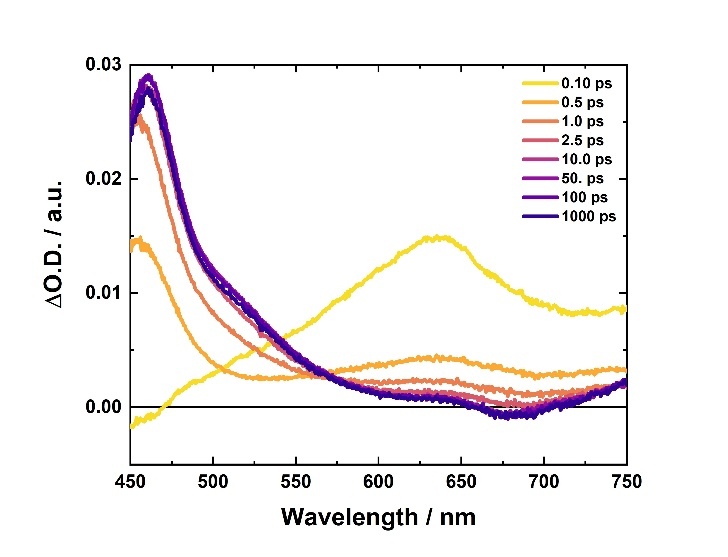


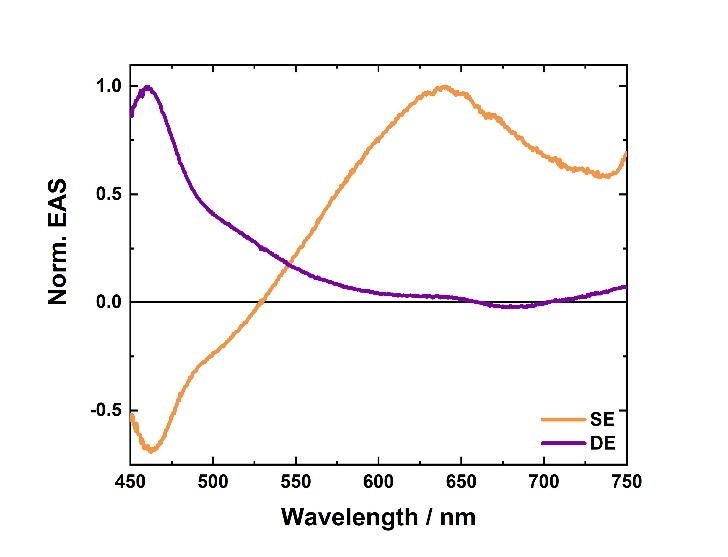


**Figure S38:** Top left: fs-TA contour plot of **F,Cl-TTH** in n-hexane obtained upon photoexcitation at 360 nm. Top right: Corresponding differential absorption changes after different time delays illustrating the excited state dynamics. Bottom: Normalized EAS of the deconvoluted excited state species SE (yellow), unrelaxed DE* (magenta), and relaxed DE (purple) states obtained via global fitting of the fs-TA raw data.


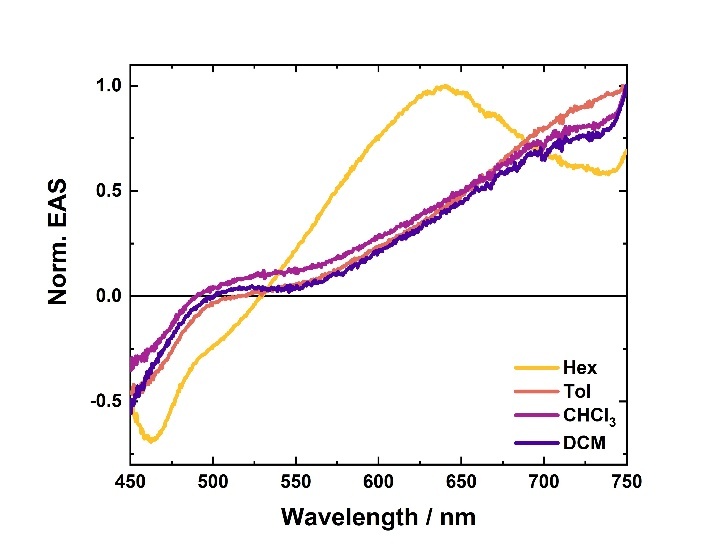

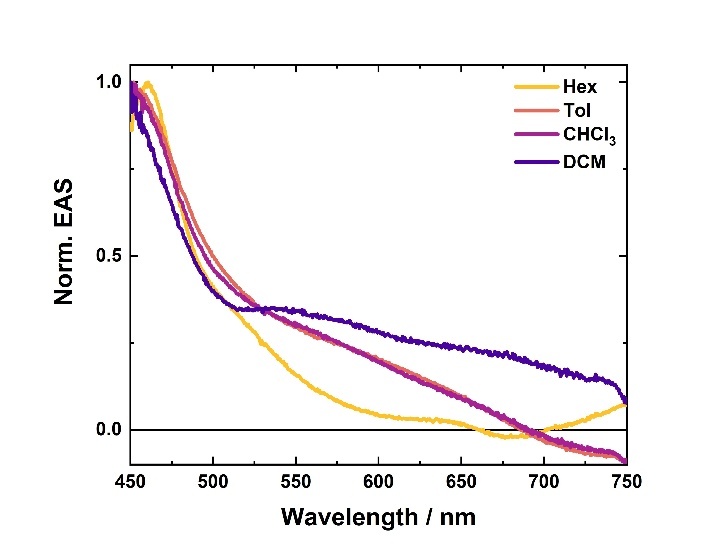


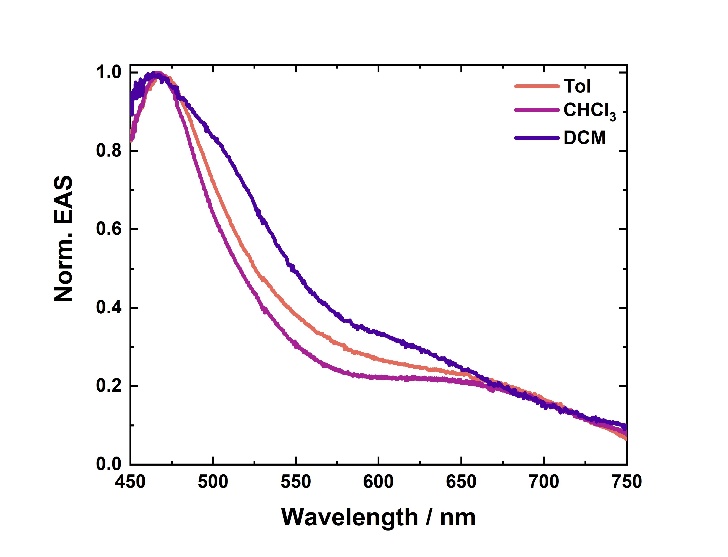


**Figure S39:** Normalized EAS of the deconvoluted species SE (top left), DE* (top right), and DE (bottom) for **F,Cl‑TTH** obtained *via* sequential global fitting of the corresponding fs-TA raw data measured in n-hexane (yellow), toluene (orange), chloroform (magenta), and dichloromethane (purple).


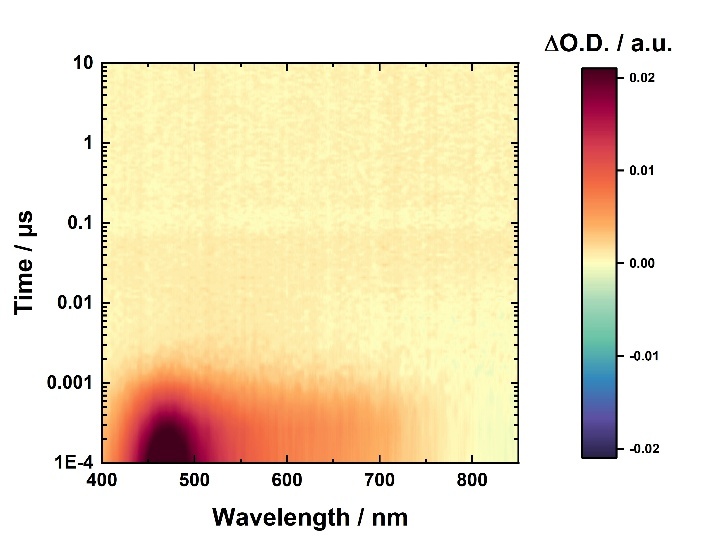

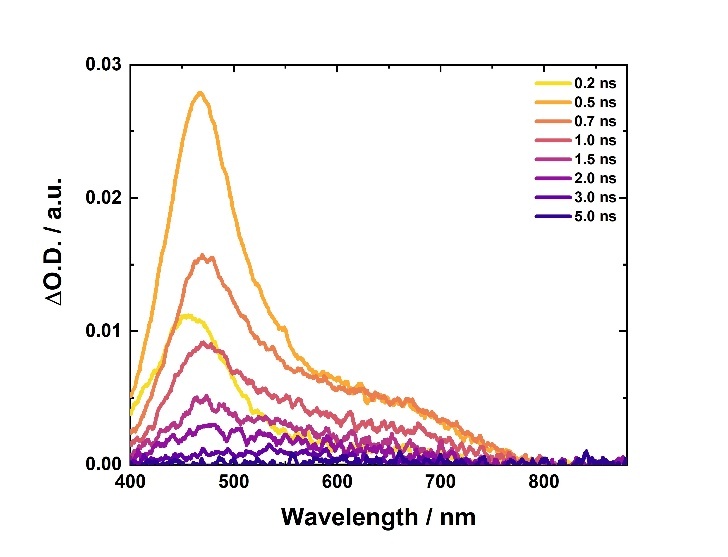


**Figure S40:** Left: ns-TA contour plot of **F,Cl-TTH** in toluene obtained upon photoexcitation at 360 nm. Right: Corresponding differential absorption changes after different time delays illustrating the excited state dynamics.


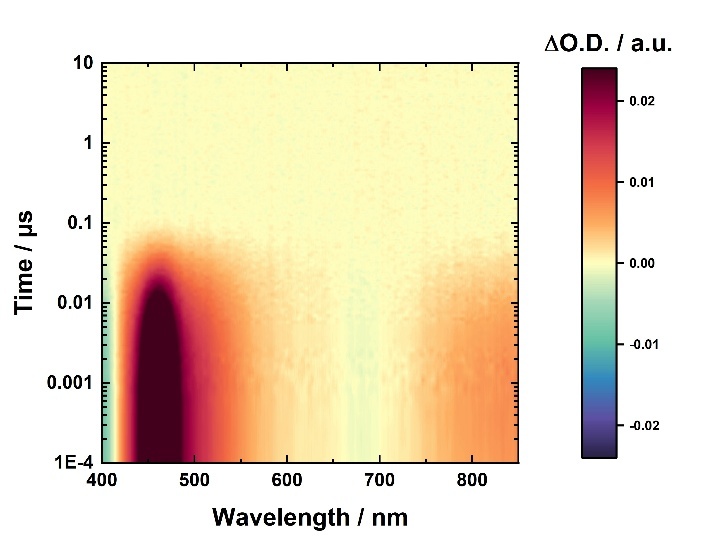

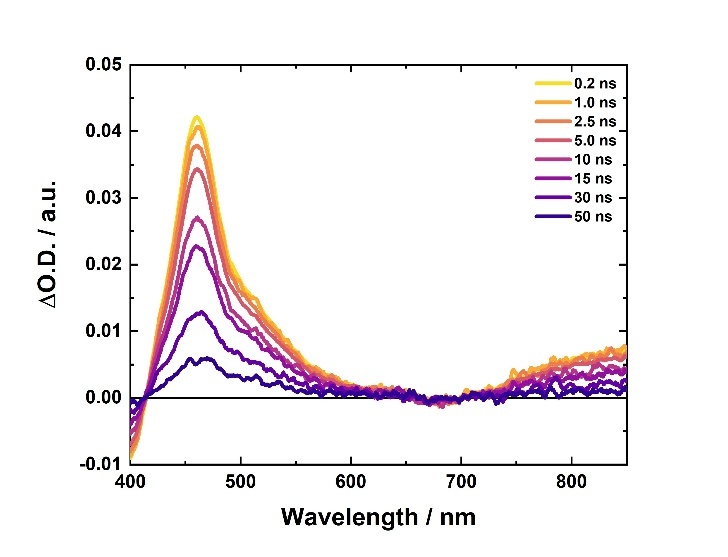


**Figure S41:** Left: ns-TA contour plot of **F,Cl-TTH** in n-hexane obtained upon photoexcitation at 360 nm. Right: Corresponding differential absorption changes after different time delays illustrating the excited state dynamics.


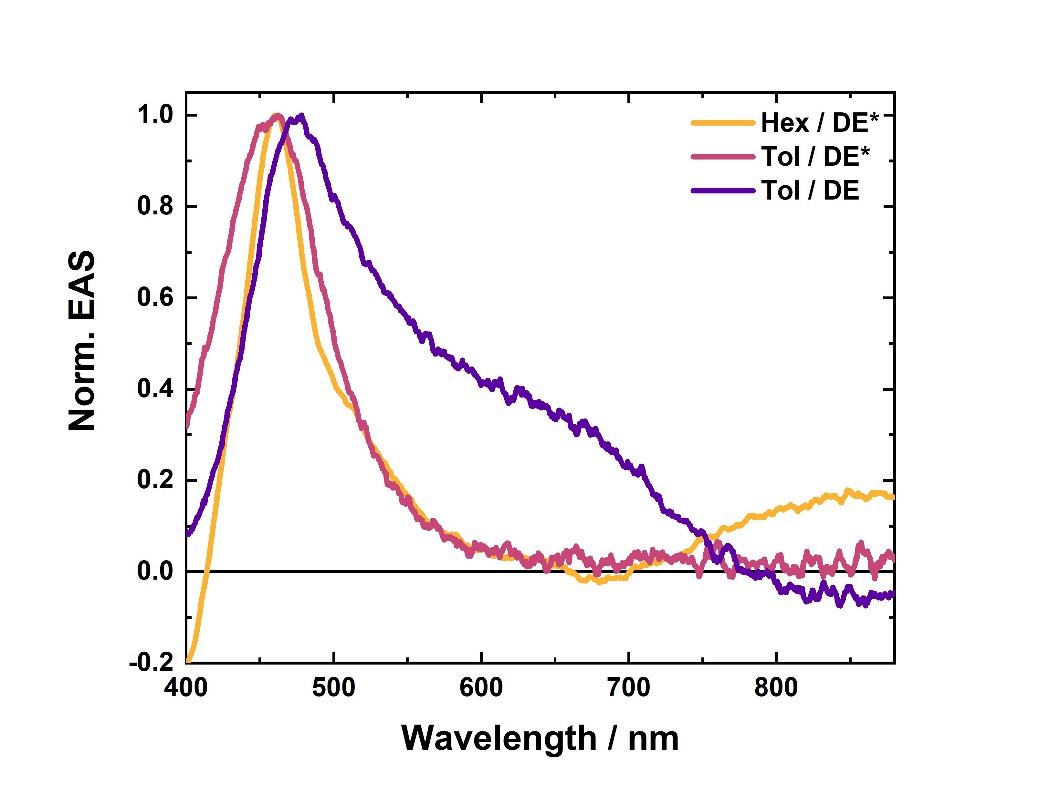


**Figure S42:** Normalized EAS of the deconvoluted species DE*/DE for **F,Cl‑TTH** obtained *via* sequential global fitting of the corresponding ns-TA raw data measured in n-hexane (yellow) and toluene (magenta and purple).

**Table S6**: Summary of all excited state lifetimes for **F,Cl-TTH** in different solvents obtained *via* sequential global fit of fs- and ns-TA raw data.

| **Solvent** | **τ(SE) / ps** | **τ(DE*)** | **τ(DE) / ps** |
| --- | --- | --- | --- |
| **n-Hexane** | ~0.1 | 23.3 ns | - |
| **Toluene** | ~0.2 | 4.23 ps | 542 |
| **Trichloromethane** | ~0.2 | 4.10 ps | 305 |
| **Dichloromethane** | <0.1 | 2.42 ps | 52 |

# Structure determination from synchrotron SCXRD

The crystal structure of **F,Cl-TTH** and **Cl,F-TTH** was solved using *SIR2019*^3^*,* a software able to determine the space group and the crystal structure by exploiting minimal prior information concerning the unit cell parameters, the observed diffraction intensities and the expected chemical formula. For both of them the structure model located by *SIR2019* was refined by full-matrix least-squares techniques using *SHELXL2014/*7^4^ and completed by placing at calculated positions the missing H atoms. Non-hydrogen atoms were refined anisotropically; the atomic coordinates of H atoms were refined according to a riding model. The constraints on the isotropic *U* value of H atoms were *U*_iso_(H)=1.2 *U*_eq_(C), for the aromatic rings and the methylene groups of the hexane solvent, and *U*_iso_(H)=1.5 *U*_eq_(C) for the methyl groups of the hexane solvent.

In the case of **F,Cl-TTH**, a not negligible deviation from planarity was observed for its C1−C2−C5−C3−C12−C18 central ring, assuming an unusual boat conformation (see **Figure 4a** and Figure S43) whose rarity is confirmed by the analysis of its molecular geometry carried out using the CCDC (Cambridge Crystallographic Data Center) software *Mogul*,^8^ searching for known structures stored in the Cambridge Structural Database (CSD)^15^ and characterized by at least one ring with similar boat conformation. Among the few crystalline compounds in the CSD best matching the geometry of the C1−C2−C5−C3−C12−C18 central ring, two structures were selected, *i.e*., **pQDM_1**^16^ and **pQDM_2**^17^ (see **Figure 4b**), characterized by a single-ring central bridge and the lowest RMSD (root mean square deviation) values of torsions from the reference C1−C2−C5−C3−C12−C18 ring (*i.e*., RSMD = 1.564° and 4.312° for **pQDM_1** and **pQDM_2**, respectively).

In the case of **Cl,F-TTH**, the distortion of the central ring C9−C13−C16−C18−C31−C23 was negligible; if the least-squares plane through the four central carbon atoms at the basis of the core ring (*i.e*., C13, C16, C31 and C23) is considered as reference plane, the ring distortion can be quantified *via* the ‘bending angles’ (BAs) between the reference plane and the two slightly inclined planes through the triplets (C16, C18, C31) and (C9, C18, C23), equal to 2.62° and 3.70°, respectively. Similarly, for **TTH**^18^ the degree of distortion of the core ring C1−C2−C3−C17−C18−C19 can be calculated by considering the reference plane through C1, C2, C17 and C18 and the two planes through (C2, C3,C17) and (C1,C18;C19); also for **TTH** the corresponding BAs values are small, equal to 2.51° and 8.08°, respectively.

For **F,Cl-TTH** an important role was carried out by the halogen-halogen interactions C—X⋯ C—X, where X= Cl and F. The intermolecular Cl···Cl distances, equal to 3.587 an 3.720 Å, and the intra- and intermolecular F···F distances, equal to 3.183 and 3.689 Å, respectively (see Figure S44), were in agreement with the literature values.^19,20^ The halogen-halogen interactions contributed to the deviation from planarity of the central ring, assuming the boat conformation (see Figure S43), and to the crystal packing stabilization, in cooperation with additional weak non-covalent intermolecular interactions, *f.e*., C—H⋯π interactions^21^ and interactions between non-parallel aromatic rings^22^ (see Figure S43).

Both **F,Cl-TTH** and **Cl,F-TTH** were characterized by the alternation of the bond lengths in the *p*-xylylene framework, typical feature of quinoidal configuration, as occurred also in **TTH** and other similar Thiele like compounds.^14^

If for **F,Cl-TTH, Cl,F-TTH** and **TTH** the geometry of the central ring and the lengths of the C−C exocyclic bonds are compared (see Figure S45), it can be observed the following:

- **F,Cl-TTH** was characterized by the longest bonds in the central ring, with bond lengths lying in the range 1.476-1.483 Å, wider than the corresponding range observed for **Cl,F-TTH** (*i.e*., 1.443-1.445 Å) and **TTH** (*i.e*., 1.429-1.455 Å). This difference could be ascribed to the not negligible stretching action of the halogen-halogen contacts;
- the lengths of the C−C exocyclic bonds of **F,Cl-TTH** (*i.e*., 1.351 and 1.352 Å) were the shortest ones; in the case of **Cl,F-TTH** the bond length was 1.382 Å, *i.e*., closer to the longest values observed for **TTH** (*i.e*., 1.388 and 1.401 Å);


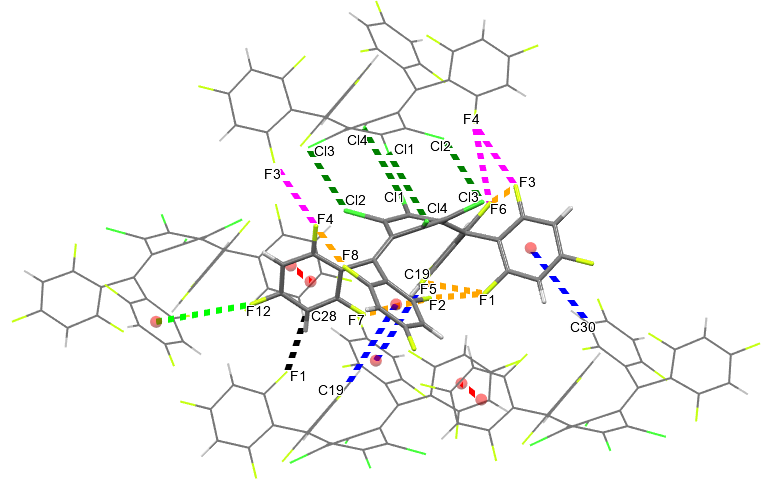


**Figure S43:** A view of the local environment of the **F,Cl-TTH** (capped sticks) and its nearest surrounding molecule (wireframes); centroids (*i.e*., *Cg*s) are drawn with red spheres. Some weak non-covalent interactions are shown, represented by coloured dashed lines: Cl⋯Cl intermolecular interactions^19,20^ in green (Cl1⋯Cl4 and Cl2⋯Cl4 lengths equal to 3.720 and 3.587 Å, respectively); F⋯F intermolecular interactions^20^ in magenta (F3**···**F4 and F4**···**F6 lengths equal to 3.689 and 3.445 Å, respectively); F⋯F intramolecular interactions^20^ in orange (F3**···**F6, F4**···**F8, F1**···**F5, F2**···**F7 and F1**···**F2 lengths equal to 2.803, 2.827, 2.973, 3.087, and 3.183 Å, respectively); F**···**π interactions in light green (F12**··***Cg* length equal to 4.131 Å), C—H⋯π interactions^21^ in blue (C19**···***Cg* and C30**···***Cg* lengths equal to 4.358 and 4.5876 Å, respectively); interactions between non-parallel aromatic rings^22^ (distance between centroids *Cg* **··***Cg* equal to 4.537 Å) in red. One of the hydrogen bonds listed in Table S3 (*i.e*., C28**···**F1, with *D*···*A* length equal to 3.187 Å) is also shown in dashed black line. The atomic labels refer to atoms (both symmetry independent and symmetry equivalent) involved in the selected interactions.


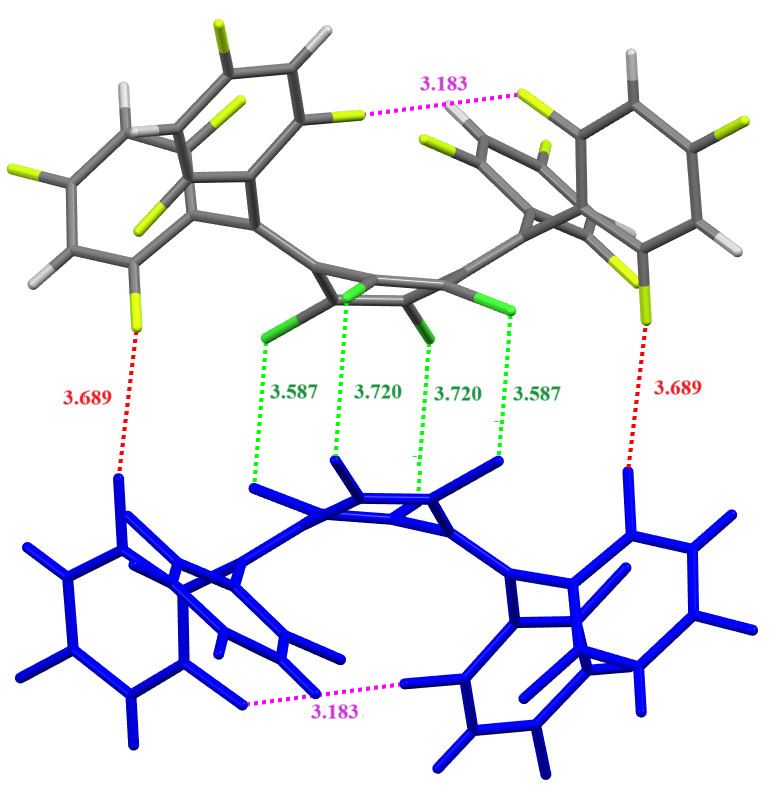


**Figure S44:** A view of the asymmetric unit (blue molecule) of **F,Cl-TTH** and one of the nearest symmetry equivalent molecules (colour setting by atomic species: yellow, green, light grey and white for F, Cl, C and H, respectively) showing intra-halogen interaction (F**···**F length equal to 3.183 Å in magenta dashed line) and inter-halogen interactions (Cl**···**Cl lengths equal to 3.587 and 3.720 Å in dashed green lines, and F**···**F length equal to 3.689 Å. in dashed red lines), cooperatively contributing to the boat conformation of the core ring.


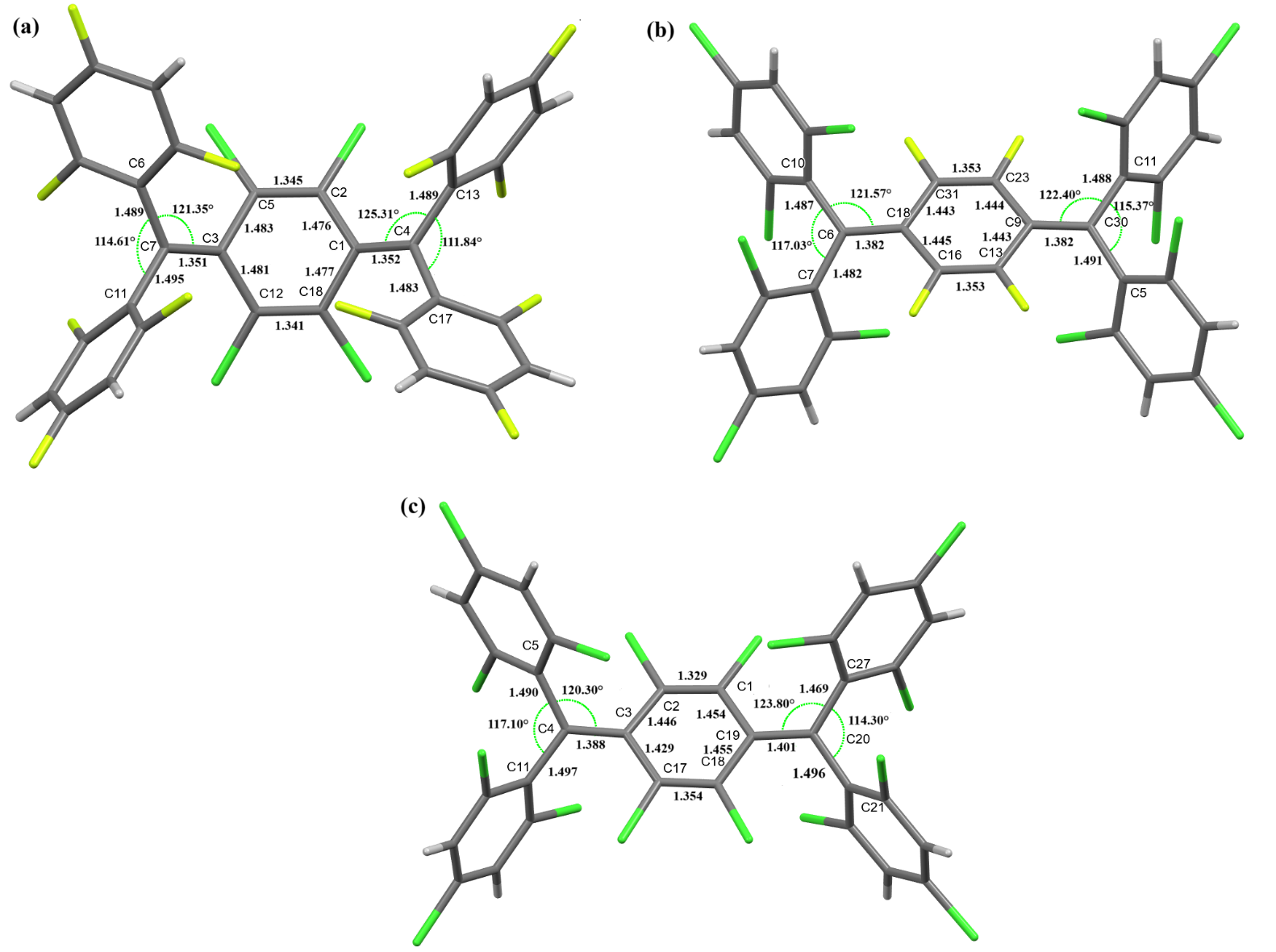


**Figure S45**: A view of the asymmetric unit of **F,Cl-TTH** (a), **Cl,F-TTH** (b) and **TTH** (c) showing some selected bond lengths (Å) and bond angles (colour setting by atomic species: yellow, green, light grey and white for F, Cl, C and H, respectively). In the case of **Cl,F-TTH** the hexane solvent is omitted for clarity.

**Table S7:** Crystal data**,** data collection and structure refinement details for **F,Cl-TTH** and **Cl,F-TTH**.

| *Crystal data* | | | |  |
| --- | --- | --- | --- | --- |
| CCDC Deposition Nymber | 2492420 | | 2492424 | |
| Chemical formula | C_32_H_8_Cl_4_F_12_ | | C_38_H_22_Cl_12_F_4_ | |
| *M*_r_ | 762.18 | | 979.95 | |
| Crystal system, space group | Triclinic, *P*-1 | | Monoclinic, *P*2_1_/*c* | |
| Temperature (K) | 100 | | 100 | |
| *a*, *b*, *c* (Å) | 10.402 (2), 12.267 (1), 13.25 (1) | | 11.731 (2), 22.110 (1), 15.144 (1) | |
| α, β, γ (°) | 106.52 (3), 98.64 (2), 110.42 (3) | | 90.00, 97.10 (3), 90.00 | |
| *V* (Å^3^) | 1459.4 (12) | | 3897.8 (8) | |
| *Z* | 2 | | 4 | |
| Radiation type | Synchrotron, λ = 0.68693 Å | | Synchrotron, λ = 0.68693 Å | |
| μ (mm^-1^) | 0.46 | | 0.81 | |
| Crystal size (mm) | 0.15 × 0.1 × 0.05 | | 0.15 × 0.15 × 0.02 | |
| *Data collection* | | | | |
| Diffractometer | ESRF BM07 MiniDiff + Kappa | | ESRF BM07 MiniDiff + Kappa | |
| Absorption correction | Empirical (using intensity measurements)  Kabsch, W. (2010)^1^ | | Empirical (using intensity measurements)  Kabsch, W. (2010) ^1^ | |
| *T*_min_, *T*_max_ | 0.80, 0.93 | | 0.78, 0.94 | |
| No. of measured, independent and observed [*I* > 2σ(*I*)] reflections | 119166, 6965, 6724 | | 294113, 9176, 8489 | |
| *R*_int_ | 0.054 | | 0.058 | |
| (sin θ/λ)_max_ (Å^-1^) | 0.667 | | 0.667 | |
| θ_min_, θ_max_ (°), | 1.8, 27.3 | | 1.8, 27.3 | |
| *h*_min_ , *h*_max_  *k*_min_ , *k*_max_  *l*_min_ , *l*_max_ | -13, 13  -16, 16  -17, 17 | | -15, 15  -29, 29  -20, 20 | |
| *Refinement* | | |  | |
| *R*[*F*^2^ > 2σ(*F*^2^)], *wR*(*F*^2^), *S* | 0.029, 0.080, 1.05 | | 0.045, 0.120, 1.04 | |
| No. of reflections | 6965 | | 9176 | |
| No. of parameters | 433 | | 489 | |
| H-atom treatment | H-atom parameters constrained | | H-atom parameters constrained | |
| Δρ_max_, Δρ_min_ (e Å^-3^) | 0.43, -0.41 | | 1.68, -1.02 | |
| *Special details* | | | | |
| *Geometry*. All esds (except the esd in the dihedral angle between two l.s. planes) are estimated using the full covariance matrix. The cell esds are taken into account individually in the estimation of esds in distances, angles and torsion angles; correlations between esds in cell parameters are only used when they are defined by crystal symmetry. An approximate (isotropic) treatment of cell esds is used for estimating esds involving l.s. planes. | | | | |
| *Computer Programs* | | *Applications* | | |
| - *SIR2019*^3^ | | Structure solution | | |
| - *SHELXL2014/7*^4^ | | Structure refinement | | |
| - WinGX^5^ | | Preparation of material for publication | | |
| - *publCIF*^6^ | | Preparation of material for publication | | |
| - *Mercury*^7^ | | Molecular graphics | | |
| - *Mogul*^8^ | | Molecular geometry analysis | | |
| - *CheckCIF*^9^ | | CIF validation | | |

**Table S8:** Fractional atomic coordinates, isotropic or equivalent isotropic displacement parameters (Å^2^) and geometric parameters (Å,°) for **F,Cl-TTH**.

|  | *x* | *y* | *z* | *U*_iso_*/*U*_eq_ |
| --- | --- | --- | --- | --- |
| Cl1 | 0.42952 (3) | 0.31469 (2) | 0.85973 (2) | 0.01895 (7) |
| Cl2 | 0.70715 (3) | 0.55277 (3) | 0.91630 (2) | 0.02020 (7) |
| Cl3 | 0.14885 (3) | 0.59519 (3) | 0.92842 (2) | 0.02061 (7) |
| Cl4 | 0.42382 (3) | 0.82830 (3) | 0.96613 (3) | 0.02391 (7) |
| F1 | 0.08026 (9) | 0.42263 (8) | 0.57234 (6) | 0.02799 (17) |
| F2 | 0.36136 (8) | 0.67609 (7) | 0.66443 (7) | 0.02588 (16) |
| F3 | -0.04765 (9) | 0.30527 (8) | 0.86255 (7) | 0.02759 (17) |
| F4 | 0.88956 (8) | 0.84572 (8) | 0.98280 (6) | 0.03038 (18) |
| F5 | 0.33401 (8) | 0.36321 (7) | 0.60049 (6) | 0.02470 (16) |
| F6 | 0.01787 (9) | 0.11234 (8) | 0.74374 (8) | 0.03264 (18) |
| F7 | 0.62641 (9) | 0.63831 (8) | 0.61409 (6) | 0.03094 (18) |
| F8 | 0.79074 (8) | 1.00128 (7) | 0.90343 (8) | 0.03384 (19) |
| F9 | -0.37192 (9) | 0.36455 (9) | 0.62195 (10) | 0.0456 (3) |
| F10 | 0.49657 (10) | 1.07859 (9) | 0.65616 (11) | 0.0543 (3) |
| F11 | 0.21073 (10) | -0.06619 (9) | 0.47942 (8) | 0.0426 (2) |
| F12 | 1.11894 (11) | 0.75057 (12) | 0.71365 (11) | 0.0558 (3) |
| C1 | 0.53147 (11) | 0.66579 (10) | 0.86383 (9) | 0.01534 (19) |
| C2 | 0.54265 (12) | 0.55217 (10) | 0.87322 (9) | 0.0160 (2) |
| C3 | 0.28092 (11) | 0.45384 (10) | 0.82146 (9) | 0.01486 (19) |
| C4 | 0.61804 (11) | 0.74413 (10) | 0.82449 (9) | 0.0162 (2) |
| C5 | 0.42216 (12) | 0.44977 (10) | 0.84986 (9) | 0.01545 (19) |
| C6 | 0.17478 (12) | 0.24367 (10) | 0.67577 (9) | 0.0180 (2) |
| C7 | 0.16455 (11) | 0.35701 (10) | 0.74670 (9) | 0.0155 (2) |
| C8 | -0.01724 (13) | 0.39120 (11) | 0.62781 (10) | 0.0207 (2) |
| C9 | 0.10167 (13) | 0.12474 (11) | 0.67610 (11) | 0.0236 (2) |
| C10 | 0.75201 (14) | 0.69095 (12) | 0.69182 (10) | 0.0230 (2) |
| C11 | 0.02237 (11) | 0.36129 (10) | 0.71741 (9) | 0.0169 (2) |
| C12 | 0.28698 (12) | 0.57916 (10) | 0.87813 (9) | 0.0160 (2) |
| C13 | 0.75165 (12) | 0.74026 (11) | 0.79954 (10) | 0.0181 (2) |
| C14 | -0.08033 (13) | 0.33258 (11) | 0.77318 (11) | 0.0214 (2) |
| C15 | -0.21299 (13) | 0.33340 (12) | 0.74342 (13) | 0.0272 (3) |
| H15 | -0.2804 | 0.3144 | 0.7839 | 0.033* |
| C16 | 0.45499 (12) | 0.79649 (11) | 0.70387 (10) | 0.0205 (2) |
| C17 | 0.57838 (12) | 0.83393 (10) | 0.78689 (10) | 0.0180 (2) |
| C18 | 0.40591 (12) | 0.67978 (10) | 0.89486 (9) | 0.0168 (2) |
| C19 | 0.27363 (14) | 0.14889 (13) | 0.53604 (11) | 0.0269 (3) |
| H19 | 0.3310 | 0.1567 | 0.4868 | 0.032* |
| C20 | 0.25993 (12) | 0.25083 (11) | 0.60383 (10) | 0.0207 (2) |
| C21 | 0.67085 (13) | 0.95951 (12) | 0.82239 (11) | 0.0240 (2) |
| C22 | -0.14826 (14) | 0.39257 (12) | 0.59192 (12) | 0.0272 (3) |
| H22 | -0.1716 | 0.4126 | 0.5294 | 0.033* |
| C23 | 1.00905 (14) | 0.80046 (14) | 0.85110 (13) | 0.0309 (3) |
| H23 | 1.0979 | 0.8384 | 0.9058 | 0.037* |
| C24 | 0.11359 (14) | 0.01959 (12) | 0.61288 (12) | 0.0298 (3) |
| H24 | 0.0649 | -0.0599 | 0.6168 | 0.036* |
| C25 | 0.99858 (16) | 0.74761 (15) | 0.74206 (15) | 0.0348 (3) |
| C26 | 0.64669 (14) | 1.04395 (12) | 0.78208 (14) | 0.0324 (3) |
| H26 | 0.7117 | 1.1294 | 0.8100 | 0.039* |
| C27 | -0.24323 (13) | 0.36299 (12) | 0.65268 (13) | 0.0302 (3) |
| C28 | 0.87288 (17) | 0.69260 (14) | 0.65985 (13) | 0.0323 (3) |
| H28 | 0.8693 | 0.6576 | 0.5851 | 0.039* |
| C29 | 0.88380 (13) | 0.79561 (12) | 0.87721 (11) | 0.0230 (2) |
| C30 | 0.42417 (13) | 0.87574 (12) | 0.65800 (12) | 0.0277 (3) |
| H30 | 0.3392 | 0.8471 | 0.6013 | 0.033* |
| C31 | 0.52283 (15) | 0.99832 (13) | 0.69868 (14) | 0.0334 (3) |
| C32 | 0.19952 (15) | 0.03559 (13) | 0.54389 (11) | 0.0302 (3) |

*Atomic displacement parameters (Å^2^)*

|  | *U*^11^ | *U*^22^ | *U*^33^ | *U*^12^ | *U*^13^ | *U*^23^ |
| --- | --- | --- | --- | --- | --- | --- |
| Cl1 | 0.02137 (13) | 0.01968 (13) | 0.01798 (13) | 0.01165 (10) | 0.00186 (10) | 0.00777 (10) |
| Cl2 | 0.01527 (12) | 0.02799 (14) | 0.02289 (14) | 0.01251 (10) | 0.00463 (10) | 0.01296 (11) |
| Cl3 | 0.01782 (13) | 0.02371 (13) | 0.02335 (14) | 0.01147 (10) | 0.00934 (10) | 0.00738 (10) |
| Cl4 | 0.02357 (14) | 0.01830 (13) | 0.02898 (15) | 0.01020 (11) | 0.00976 (11) | 0.00411 (11) |
| F1 | 0.0325 (4) | 0.0411 (4) | 0.0219 (4) | 0.0211 (4) | 0.0121 (3) | 0.0179 (3) |
| F2 | 0.0185 (3) | 0.0200 (3) | 0.0299 (4) | 0.0017 (3) | -0.0059 (3) | 0.0105 (3) |
| F3 | 0.0286 (4) | 0.0341 (4) | 0.0318 (4) | 0.0154 (3) | 0.0171 (3) | 0.0210 (3) |
| F4 | 0.0214 (4) | 0.0389 (4) | 0.0213 (4) | 0.0110 (3) | -0.0028 (3) | 0.0038 (3) |
| F5 | 0.0248 (4) | 0.0308 (4) | 0.0228 (4) | 0.0132 (3) | 0.0111 (3) | 0.0114 (3) |
| F6 | 0.0318 (4) | 0.0262 (4) | 0.0423 (5) | 0.0089 (3) | 0.0141 (4) | 0.0178 (3) |
| F7 | 0.0333 (4) | 0.0404 (4) | 0.0166 (3) | 0.0165 (4) | 0.0030 (3) | 0.0073 (3) |
| F8 | 0.0212 (4) | 0.0239 (4) | 0.0408 (5) | 0.0026 (3) | -0.0112 (3) | 0.0081 (3) |
| F9 | 0.0173 (4) | 0.0434 (5) | 0.0757 (7) | 0.0166 (4) | 0.0014 (4) | 0.0220 (5) |
| F10 | 0.0335 (5) | 0.0338 (5) | 0.0915 (9) | 0.0076 (4) | -0.0114 (5) | 0.0397 (5) |
| F11 | 0.0448 (5) | 0.0336 (5) | 0.0379 (5) | 0.0276 (4) | -0.0069 (4) | -0.0083 (4) |
| F12 | 0.0358 (5) | 0.0741 (8) | 0.0814 (8) | 0.0334 (5) | 0.0407 (6) | 0.0362 (6) |
| C1 | 0.0137 (4) | 0.0187 (5) | 0.0131 (5) | 0.0077 (4) | 0.0014 (4) | 0.0049 (4) |
| C2 | 0.0162 (5) | 0.0230 (5) | 0.0124 (5) | 0.0116 (4) | 0.0035 (4) | 0.0076 (4) |
| C3 | 0.0154 (5) | 0.0189 (5) | 0.0139 (5) | 0.0090 (4) | 0.0049 (4) | 0.0082 (4) |
| C4 | 0.0135 (5) | 0.0191 (5) | 0.0148 (5) | 0.0071 (4) | 0.0009 (4) | 0.0053 (4) |
| C5 | 0.0179 (5) | 0.0196 (5) | 0.0122 (5) | 0.0112 (4) | 0.0034 (4) | 0.0064 (4) |
| C6 | 0.0158 (5) | 0.0199 (5) | 0.0171 (5) | 0.0090 (4) | 0.0004 (4) | 0.0053 (4) |
| C7 | 0.0154 (5) | 0.0195 (5) | 0.0153 (5) | 0.0089 (4) | 0.0054 (4) | 0.0088 (4) |
| C8 | 0.0201 (5) | 0.0218 (5) | 0.0193 (5) | 0.0096 (4) | 0.0033 (4) | 0.0063 (4) |
| C9 | 0.0194 (5) | 0.0228 (6) | 0.0254 (6) | 0.0081 (4) | 0.0001 (4) | 0.0083 (5) |
| C10 | 0.0256 (6) | 0.0265 (6) | 0.0217 (6) | 0.0133 (5) | 0.0080 (5) | 0.0119 (5) |
| C11 | 0.0137 (5) | 0.0177 (5) | 0.0188 (5) | 0.0067 (4) | 0.0034 (4) | 0.0062 (4) |
| C12 | 0.0160 (5) | 0.0213 (5) | 0.0141 (5) | 0.0110 (4) | 0.0046 (4) | 0.0069 (4) |
| C13 | 0.0152 (5) | 0.0212 (5) | 0.0205 (5) | 0.0087 (4) | 0.0055 (4) | 0.0098 (4) |
| C14 | 0.0192 (5) | 0.0204 (5) | 0.0277 (6) | 0.0086 (4) | 0.0091 (5) | 0.0110 (4) |
| C15 | 0.0170 (5) | 0.0212 (5) | 0.0453 (8) | 0.0081 (4) | 0.0123 (5) | 0.0124 (5) |
| C16 | 0.0146 (5) | 0.0191 (5) | 0.0257 (6) | 0.0050 (4) | 0.0019 (4) | 0.0095 (4) |
| C17 | 0.0143 (5) | 0.0196 (5) | 0.0207 (5) | 0.0073 (4) | 0.0035 (4) | 0.0085 (4) |
| C18 | 0.0178 (5) | 0.0190 (5) | 0.0156 (5) | 0.0104 (4) | 0.0042 (4) | 0.0060 (4) |
| C19 | 0.0244 (6) | 0.0356 (7) | 0.0196 (6) | 0.0198 (5) | 0.0003 (5) | 0.0021 (5) |
| C20 | 0.0186 (5) | 0.0262 (6) | 0.0172 (5) | 0.0126 (4) | 0.0008 (4) | 0.0057 (4) |
| C21 | 0.0147 (5) | 0.0221 (6) | 0.0299 (6) | 0.0054 (4) | -0.0010 (5) | 0.0087 (5) |
| C22 | 0.0248 (6) | 0.0261 (6) | 0.0279 (6) | 0.0129 (5) | -0.0031 (5) | 0.0080 (5) |
| C23 | 0.0160 (5) | 0.0349 (7) | 0.0448 (8) | 0.0115 (5) | 0.0078 (5) | 0.0176 (6) |
| C24 | 0.0258 (6) | 0.0200 (6) | 0.0340 (7) | 0.0092 (5) | -0.0064 (5) | 0.0043 (5) |
| C25 | 0.0271 (6) | 0.0402 (8) | 0.0547 (9) | 0.0210 (6) | 0.0261 (7) | 0.0256 (7) |
| C26 | 0.0209 (6) | 0.0206 (6) | 0.0501 (9) | 0.0040 (5) | -0.0006 (6) | 0.0160 (6) |
| C27 | 0.0152 (5) | 0.0231 (6) | 0.0478 (8) | 0.0094 (5) | 0.0002 (5) | 0.0088 (5) |
| C28 | 0.0400 (8) | 0.0387 (7) | 0.0337 (7) | 0.0238 (6) | 0.0235 (6) | 0.0187 (6) |
| C29 | 0.0172 (5) | 0.0266 (6) | 0.0249 (6) | 0.0095 (4) | 0.0037 (5) | 0.0096 (5) |
| C30 | 0.0178 (5) | 0.0271 (6) | 0.0371 (7) | 0.0079 (5) | -0.0022 (5) | 0.0166 (5) |
| C31 | 0.0242 (6) | 0.0268 (6) | 0.0526 (9) | 0.0108 (5) | 0.0013 (6) | 0.0237 (6) |
| C32 | 0.0277 (6) | 0.0276 (6) | 0.0266 (6) | 0.0187 (5) | -0.0080 (5) | -0.0036 (5) |

*Geometric parameters (Å, º)*

| Cl1—C5 | 1.7255 (11) | C6—C20 | 1.3957 (18) |
| --- | --- | --- | --- |
| Cl2—C2 | 1.7173 (12) | C6—C7 | 1.4892 (16) |
| Cl3—C12 | 1.7197 (12) | C7—C11 | 1.4947 (15) |
| Cl4—C18 | 1.7256 (13) | C8—C22 | 1.3820 (17) |
| F1—C8 | 1.3527 (15) | C8—C11 | 1.3856 (19) |
| F2—C16 | 1.3444 (15) | C9—C24 | 1.3818 (19) |
| F3—C14 | 1.3469 (17) | C10—C28 | 1.3812 (18) |
| F4—C29 | 1.3412 (18) | C10—C13 | 1.3839 (19) |
| F5—C20 | 1.3416 (15) | C11—C14 | 1.3906 (16) |
| F6—C9 | 1.3437 (17) | C12—C18 | 1.3415 (17) |
| F7—C10 | 1.3467 (17) | C13—C29 | 1.3913 (17) |
| F8—C21 | 1.3452 (16) | C14—C15 | 1.3815 (17) |
| F9—C27 | 1.3478 (15) | C15—C27 | 1.375 (2) |
| F10—C31 | 1.3486 (16) | C16—C30 | 1.3828 (17) |
| F11—C32 | 1.3517 (15) | C16—C17 | 1.3948 (17) |
| F12—C25 | 1.3521 (15) | C17—C21 | 1.3919 (17) |
| C1—C4 | 1.3522 (16) | C19—C32 | 1.377 (2) |
| C1—C2 | 1.4762 (15) | C19—C20 | 1.3838 (17) |
| C1—C18 | 1.4772 (15) | C21—C26 | 1.3702 (18) |
| C2—C5 | 1.3447 (17) | C22—C27 | 1.382 (2) |
| C3—C7 | 1.3508 (17) | C23—C25 | 1.375 (3) |
| C3—C12 | 1.4808 (15) | C23—C29 | 1.3834 (17) |
| C3—C5 | 1.4827 (15) | C24—C32 | 1.378 (2) |
| C4—C17 | 1.4830 (16) | C25—C28 | 1.375 (2) |
| C4—C13 | 1.4893 (15) | C26—C31 | 1.383 (2) |
| C6—C9 | 1.3918 (17) | C30—C31 | 1.379 (2) |
|  |  |  |  |
| C4—C1—C2 | 125.15 (10) | F3—C14—C11 | 118.31 (11) |
| C4—C1—C18 | 122.95 (10) | C15—C14—C11 | 123.54 (12) |
| C2—C1—C18 | 111.75 (10) | C27—C15—C14 | 117.06 (12) |
| C5—C2—C1 | 118.88 (10) | F2—C16—C30 | 117.75 (11) |
| C5—C2—Cl2 | 120.90 (9) | F2—C16—C17 | 118.47 (11) |
| C1—C2—Cl2 | 120.21 (9) | C30—C16—C17 | 123.77 (11) |
| C7—C3—C12 | 124.69 (10) | C21—C17—C16 | 115.07 (11) |
| C7—C3—C5 | 123.44 (10) | C21—C17—C4 | 121.87 (10) |
| C12—C3—C5 | 111.70 (10) | C16—C17—C4 | 122.59 (11) |
| C1—C4—C17 | 122.37 (10) | C12—C18—C1 | 120.72 (10) |
| C1—C4—C13 | 125.31 (10) | C12—C18—Cl4 | 121.05 (9) |
| C17—C4—C13 | 111.84 (10) | C1—C18—Cl4 | 117.87 (9) |
| C2—C5—C3 | 120.23 (10) | C32—C19—C20 | 116.27 (13) |
| C2—C5—Cl1 | 120.65 (9) | F5—C20—C19 | 117.79 (12) |
| C3—C5—Cl1 | 119.02 (9) | F5—C20—C6 | 118.28 (10) |
| C9—C6—C20 | 115.52 (11) | C19—C20—C6 | 123.93 (12) |
| C9—C6—C7 | 123.14 (11) | F8—C21—C26 | 117.80 (12) |
| C20—C6—C7 | 121.34 (11) | F8—C21—C17 | 117.67 (12) |
| C3—C7—C6 | 121.35 (10) | C26—C21—C17 | 124.51 (12) |
| C3—C7—C11 | 123.50 (10) | C8—C22—C27 | 115.81 (13) |
| C6—C7—C11 | 114.61 (10) | C25—C23—C29 | 116.68 (13) |
| F1—C8—C22 | 117.77 (12) | C32—C24—C9 | 116.80 (13) |
| F1—C8—C11 | 117.45 (11) | F12—C25—C28 | 117.92 (15) |
| C22—C8—C11 | 124.77 (12) | F12—C25—C23 | 118.24 (15) |
| F6—C9—C24 | 118.37 (12) | C28—C25—C23 | 123.84 (13) |
| F6—C9—C6 | 118.05 (11) | C21—C26—C31 | 116.53 (12) |
| C24—C9—C6 | 123.55 (13) | F9—C27—C15 | 118.24 (13) |
| F7—C10—C28 | 118.50 (12) | F9—C27—C22 | 118.18 (14) |
| F7—C10—C13 | 117.55 (11) | C15—C27—C22 | 123.58 (12) |
| C28—C10—C13 | 123.95 (13) | C25—C28—C10 | 116.37 (14) |
| C8—C11—C14 | 115.22 (11) | F4—C29—C23 | 118.45 (12) |
| C8—C11—C7 | 120.67 (10) | F4—C29—C13 | 118.22 (11) |
| C14—C11—C7 | 124.07 (11) | C23—C29—C13 | 123.33 (13) |
| C18—C12—C3 | 118.54 (10) | C31—C30—C16 | 116.67 (12) |
| C18—C12—Cl3 | 120.63 (9) | F10—C31—C30 | 118.47 (13) |
| C3—C12—Cl3 | 120.72 (9) | F10—C31—C26 | 118.09 (13) |
| C10—C13—C29 | 115.81 (11) | C30—C31—C26 | 123.43 (13) |
| C10—C13—C4 | 119.70 (11) | F11—C32—C19 | 118.08 (14) |
| C29—C13—C4 | 124.13 (11) | F11—C32—C24 | 118.03 (14) |
| F3—C14—C15 | 118.13 (11) | C19—C32—C24 | 123.89 (12) |
|  |  |  |  |
| C4—C1—C2—C5 | -141.01 (12) | F2—C16—C17—C4 | 7.01 (17) |
| C18—C1—C2—C5 | 34.55 (14) | C30—C16—C17—C4 | -171.77 (12) |
| C4—C1—C2—Cl2 | 40.43 (15) | C1—C4—C17—C21 | 130.37 (13) |
| C18—C1—C2—Cl2 | -144.01 (9) | C13—C4—C17—C21 | -57.21 (15) |
| C2—C1—C4—C17 | 161.77 (10) | C1—C4—C17—C16 | -57.96 (17) |
| C18—C1—C4—C17 | -13.32 (17) | C13—C4—C17—C16 | 114.46 (13) |
| C2—C1—C4—C13 | -9.60 (18) | C3—C12—C18—C1 | -3.85 (16) |
| C18—C1—C4—C13 | 175.31 (10) | Cl3—C12—C18—C1 | 172.44 (8) |
| C1—C2—C5—C3 | -3.50 (15) | C3—C12—C18—Cl4 | -176.84 (8) |
| Cl2—C2—C5—C3 | 175.04 (8) | Cl3—C12—C18—Cl4 | -0.55 (14) |
| C1—C2—C5—Cl1 | -179.93 (8) | C4—C1—C18—C12 | 144.66 (12) |
| Cl2—C2—C5—Cl1 | -1.39 (14) | C2—C1—C18—C12 | -31.01 (15) |
| C7—C3—C5—C2 | 144.09 (12) | C4—C1—C18—Cl4 | -42.13 (15) |
| C12—C3—C5—C2 | -31.33 (14) | C2—C1—C18—Cl4 | 142.19 (9) |
| C7—C3—C5—Cl1 | -39.42 (14) | C32—C19—C20—F5 | 178.13 (11) |
| C12—C3—C5—Cl1 | 145.16 (9) | C32—C19—C20—C6 | -1.35 (18) |
| C12—C3—C7—C6 | 166.43 (10) | C9—C6—C20—F5 | -179.45 (10) |
| C5—C3—C7—C6 | -8.39 (16) | C7—C6—C20—F5 | 0.53 (17) |
| C12—C3—C7—C11 | -4.72 (17) | C9—C6—C20—C19 | 0.02 (18) |
| C5—C3—C7—C11 | -179.55 (10) | C7—C6—C20—C19 | 180.00 (11) |
| C9—C6—C7—C3 | 119.46 (13) | C16—C17—C21—F8 | 179.12 (11) |
| C20—C6—C7—C3 | -60.51 (16) | C4—C17—C21—F8 | -8.62 (18) |
| C9—C6—C7—C11 | -68.64 (15) | C16—C17—C21—C26 | 0.5 (2) |
| C20—C6—C7—C11 | 111.39 (13) | C4—C17—C21—C26 | 172.81 (13) |
| C20—C6—C9—F6 | -179.90 (10) | F1—C8—C22—C27 | -178.38 (11) |
| C7—C6—C9—F6 | 0.12 (18) | C11—C8—C22—C27 | 0.85 (19) |
| C20—C6—C9—C24 | 1.86 (18) | F6—C9—C24—C32 | 179.52 (11) |
| C7—C6—C9—C24 | -178.11 (11) | C6—C9—C24—C32 | -2.25 (19) |
| F1—C8—C11—C14 | 178.52 (10) | C29—C23—C25—F12 | -179.92 (13) |
| C22—C8—C11—C14 | -0.71 (18) | C29—C23—C25—C28 | 0.6 (2) |
| F1—C8—C11—C7 | -3.50 (16) | F8—C21—C26—C31 | 179.85 (14) |
| C22—C8—C11—C7 | 177.27 (11) | C17—C21—C26—C31 | -1.6 (2) |
| C3—C7—C11—C8 | 95.18 (15) | C14—C15—C27—F9 | -179.95 (12) |
| C6—C7—C11—C8 | -76.52 (14) | C14—C15—C27—C22 | -0.7 (2) |
| C3—C7—C11—C14 | -87.03 (15) | C8—C22—C27—F9 | 179.16 (12) |
| C6—C7—C11—C14 | 101.27 (14) | C8—C22—C27—C15 | -0.1 (2) |
| C7—C3—C12—C18 | -140.47 (12) | F12—C25—C28—C10 | 179.85 (13) |
| C5—C3—C12—C18 | 34.89 (14) | C23—C25—C28—C10 | -0.7 (2) |
| C7—C3—C12—Cl3 | 43.24 (15) | F7—C10—C28—C25 | 179.83 (12) |
| C5—C3—C12—Cl3 | -141.40 (9) | C13—C10—C28—C25 | -0.2 (2) |
| F7—C10—C13—C29 | -178.98 (11) | C25—C23—C29—F4 | 179.73 (12) |
| C28—C10—C13—C29 | 1.06 (18) | C25—C23—C29—C13 | 0.4 (2) |
| F7—C10—C13—C4 | -5.60 (17) | C10—C13—C29—F4 | 179.50 (11) |
| C28—C10—C13—C4 | 174.44 (12) | C4—C13—C29—F4 | 6.44 (18) |
| C1—C4—C13—C10 | 103.64 (15) | C10—C13—C29—C23 | -1.13 (18) |
| C17—C4—C13—C10 | -68.51 (14) | C4—C13—C29—C23 | -174.19 (12) |
| C1—C4—C13—C29 | -83.56 (16) | F2—C16—C30—C31 | -179.05 (13) |
| C17—C4—C13—C29 | 104.28 (14) | C17—C16—C30—C31 | -0.3 (2) |
| C8—C11—C14—F3 | -178.58 (10) | C16—C30—C31—F10 | -179.93 (14) |
| C7—C11—C14—F3 | 3.52 (17) | C16—C30—C31—C26 | -0.9 (2) |
| C8—C11—C14—C15 | -0.17 (18) | C21—C26—C31—F10 | -179.20 (15) |
| C7—C11—C14—C15 | -178.07 (11) | C21—C26—C31—C30 | 1.8 (3) |
| F3—C14—C15—C27 | 179.25 (11) | C20—C19—C32—F11 | -179.66 (11) |
| C11—C14—C15—C27 | 0.83 (19) | C20—C19—C32—C24 | 0.9 (2) |
| F2—C16—C17—C21 | 179.21 (11) | C9—C24—C32—F11 | -178.64 (11) |
| C30—C16—C17—C21 | 0.42 (19) | C9—C24—C32—C19 | 0.8 (2) |

**Table S9:** Hydrogen-bond geometry (Å, º) for **F,Cl-TTH**.

| *D*—H···*A* | *D*—H | H···*A* | *D*···*A* | *D*—H···*A* |
| --- | --- | --- | --- | --- |
| C23—H23···F8^i^ | 0.95 | 2.51 | 3.281 (3) | 138 |
| C26—H26···F3^ii^ | 0.95 | 2.50 | 3.383 (2) | 154 |
| C28—H28···F1^iii^ | 0.95 | 2.26 | 3.187 (3) | 164 |
| C30—H30···F11^iv^ | 0.95 | 2.62 | 3.394 (2) | 139 |

Symmetry codes: (i) -*x*+2, -*y*+2, -*z*+2; (ii) *x*+1, *y*+1, *z*; (iii) -*x*+1, -*y*+1, -*z*+1; (iv) *x*, *y*+1, *z*.

**Table S10:** Fractional atomic coordinates, isotropic or equivalent isotropic displacement parameters (Å^2^) and geometric parameters (Å,°) for **Cl,F-TTH**.

|  | *x* | | *y* | | *z* | | *U*_iso_*/*U*_eq_ |
| --- | --- | --- | --- | --- | --- | --- | --- |
| Cl1 | 0.80279 (5) | 0.13267 (3) | | 0.20904 (4) | | 0.02867 (12) | |
| Cl2 | 0.17165 (5) | -0.04225 (3) | | -0.01140 (4) | | 0.02867 (13) | |
| Cl3 | 0.99456 (5) | 0.24699 (3) | | 0.22592 (4) | | 0.02853 (12) | |
| Cl4 | 0.78193 (5) | 0.29350 (3) | | 0.47959 (4) | | 0.02867 (12) | |
| Cl5 | 0.52945 (4) | 0.10693 (3) | | 0.02107 (5) | | 0.03099 (13) | |
| Cl6 | 0.09591 (4) | 0.19431 (3) | | 0.02667 (4) | | 0.03037 (13) | |
| Cl7 | 0.36769 (6) | 0.18104 (3) | | -0.16708 (4) | | 0.03265 (14) | |
| Cl8 | 0.25948 (6) | 0.32788 (3) | | 0.09908 (4) | | 0.03184 (13) | |
| Cl9 | 0.64478 (5) | 0.38513 (3) | | 0.31497 (5) | | 0.03344 (14) | |
| Cl10 | 0.14853 (6) | 0.38939 (3) | | -0.24308 (4) | | 0.03487 (14) | |
| Cl11 | 1.05163 (6) | 0.48369 (3) | | 0.28107 (5) | | 0.03747 (15) | |
| Cl12 | 0.98092 (6) | 0.07777 (3) | | 0.54095 (4) | | 0.03840 (15) | |
| F2 | 0.41128 (11) | 0.14756 (6) | | 0.19549 (9) | | 0.0248 (3) | |
| F4 | 0.51041 (12) | 0.31072 (7) | | 0.01254 (10) | | 0.0310 (3) | |
| F1 | 0.58559 (12) | 0.17350 (7) | | 0.31225 (9) | | 0.0290 (3) | |
| F3 | 0.68992 (11) | 0.33227 (6) | | 0.12417 (10) | | 0.0273 (3) | |
| C3 | 0.38238 (17) | 0.09850 (10) | | 0.01983 (16) | | 0.0236 (4) | |
| C4 | 0.88234 (19) | 0.18568 (11) | | 0.49765 (15) | | 0.0252 (4) | |
| H4 | 0.8985 | 0.1957 | | 0.5575 | | 0.030* | |
| C5 | 0.80232 (17) | 0.21412 (10) | | 0.34516 (14) | | 0.0205 (4) | |
| C6 | 0.35962 (17) | 0.21133 (9) | | 0.03103 (14) | | 0.0194 (4) | |
| C7 | 0.31157 (17) | 0.14926 (10) | | 0.02462 (14) | | 0.0194 (4) | |
| C8 | 0.78500 (19) | 0.37475 (11) | | 0.29492 (16) | | 0.0250 (4) | |
| C9 | 0.65366 (17) | 0.25277 (10) | | 0.22442 (14) | | 0.0205 (4) | |
| C10 | 0.31004 (17) | 0.25585 (10) | | -0.03669 (14) | | 0.0206 (4) | |
| C11 | 0.82556 (17) | 0.31687 (10) | | 0.27794 (14) | | 0.0208 (4) | |
| C12 | 0.83243 (19) | 0.15581 (10) | | 0.31896 (14) | | 0.0231 (4) | |
| C13 | 0.57288 (18) | 0.20512 (10) | | 0.23624 (15) | | 0.0218 (4) | |
| C14 | 0.14842 (18) | 0.07866 (11) | | 0.00684 (15) | | 0.0233 (4) | |
| H14 | 0.0695 | 0.0722 | | 0.0018 | | 0.028* | |
| C15 | 0.82720 (17) | 0.22644 (10) | | 0.43698 (14) | | 0.0219 (4) | |
| C16 | 0.48127 (17) | 0.19243 (10) | | 0.17573 (15) | | 0.0207 (4) | |
| C17 | 0.34093 (19) | 0.04009 (10) | | 0.01089 (17) | | 0.0261 (4) | |
| H17 | 0.3910 | 0.0076 | | 0.0099 | | 0.031* | |
| C18 | 0.44980 (17) | 0.22634 (10) | | 0.09486 (14) | | 0.0204 (4) | |
| C19 | 0.93982 (18) | 0.31442 (11) | | 0.26041 (15) | | 0.0226 (4) | |
| C20 | 0.2126 (2) | 0.35207 (11) | | -0.07454 (16) | | 0.0267 (4) | |
| H20 | 0.1807 | 0.3878 | | -0.0565 | | 0.032* | |
| C21 | 0.21198 (19) | 0.33901 (11) | | -0.16404 (16) | | 0.0261 (4) | |
| C22 | 0.8875 (2) | 0.11400 (11) | | 0.37804 (16) | | 0.0262 (4) | |
| H22 | 0.9070 | 0.0760 | | 0.3583 | | 0.031* | |
| C23 | 0.61938 (17) | 0.28844 (10) | | 0.14576 (15) | | 0.0213 (4) | |
| C24 | 0.25836 (18) | 0.28620 (11) | | -0.19234 (15) | | 0.0246 (4) | |
| H24 | 0.2574 | 0.2780 | | -0.2526 | | 0.029* | |
| C25 | 0.9126 (2) | 0.12984 (11) | | 0.46662 (16) | | 0.0269 (4) | |
| C26 | 0.26191 (19) | 0.31049 (10) | | -0.01241 (15) | | 0.0238 (4) | |
| C27 | 0.22321 (18) | 0.03081 (10) | | 0.00349 (15) | | 0.0233 (4) | |
| C28 | 1.01065 (18) | 0.36449 (11) | | 0.26234 (16) | | 0.0259 (4) | |
| H28 | 1.0868 | 0.3607 | | 0.2518 | | 0.031* | |
| C29 | 0.19323 (17) | 0.13661 (10) | | 0.01792 (14) | | 0.0214 (4) | |
| C30 | 0.75456 (17) | 0.26125 (10) | | 0.28049 (14) | | 0.0201 (4) | |
| C31 | 0.52546 (18) | 0.27699 (10) | | 0.08692 (15) | | 0.0220 (4) | |
| C32 | 0.8527 (2) | 0.42618 (11) | | 0.29598 (17) | | 0.0281 (5) | |
| H32 | 0.8227 | 0.4640 | | 0.3070 | | 0.034* | |
| C33 | 0.30651 (18) | 0.24565 (10) | | -0.12836 (15) | | 0.0224 (4) | |
| C34 | 0.9657 (2) | 0.42002 (11) | | 0.28029 (16) | | 0.0273 (5) | |
| C36 | 0.3464 (4) | 0.53744 (19) | | -0.01930 (18) | | 0.0761 (13) | |
| H36A | 0.4281 | 0.5453 | | -0.0170 | | 0.091* | |
| H36B | 0.3085 | 0.5754 | | -0.0096 | | 0.091* | |
| C37 | 0.3259 (3) | 0.49245 (15) | | 0.0546 (2) | | 0.0691 (12) | |
| H37A | 0.2441 | 0.4866 | | 0.0554 | | 0.083* | |
| H37B | 0.3599 | 0.4536 | | 0.0434 | | 0.083* | |
| C38 | 0.3804 (5) | 0.51769 (19) | | 0.14457 (17) | | 0.0775 (13) | |
| H38A | 0.3386 | 0.5532 | | 0.1600 | | 0.093* | |
| H38B | 0.4594 | 0.5293 | | 0.1409 | | 0.093* | |
| C40 | 0.3761 (4) | 0.46852 (15) | | 0.2161 (3) | | 0.0890 (18) | |
| H40A | 0.2974 | 0.4555 | | 0.2178 | | 0.107* | |
| H40B | 0.4210 | 0.4337 | | 0.2022 | | 0.107* | |
| C41 | 0.4253 (5) | 0.4948 (2) | | 0.30687 (18) | | 0.101 (2) | |
| H41A | 0.4246 | 0.4643 | | 0.3519 | | 0.152* | |
| H41B | 0.3792 | 0.5285 | | 0.3210 | | 0.152* | |
| H41C | 0.5027 | 0.5081 | | 0.3044 | | 0.152* | |
| C42 | 0.3003 (5) | 0.5124 (3) | | -0.1118 (3) | | 0.0849 (15) | |
| H42A | 0.3181 | 0.5402 | | -0.1568 | | 0.127* | |
| H42B | 0.2185 | 0.5075 | | -0.1157 | | 0.127* | |
| H42C | 0.3354 | 0.4740 | | -0.1204 | | 0.127* | |

*Atomic displacement parameters (Å^2^)*

|  | *U*^11^ | *U*^22^ | *U*^33^ | *U*^12^ | *U*^13^ | *U*^23^ |
| --- | --- | --- | --- | --- | --- | --- |
| Cl1 | 0.0336 (3) | 0.0294 (3) | 0.0210 (2) | 0.0039 (2) | -0.0044 (2) | -0.00593 (19) |
| Cl2 | 0.0243 (3) | 0.0263 (3) | 0.0346 (3) | -0.00719 (19) | 0.0003 (2) | 0.0001 (2) |
| Cl3 | 0.0199 (2) | 0.0344 (3) | 0.0323 (3) | 0.00799 (19) | 0.0070 (2) | 0.0030 (2) |
| Cl4 | 0.0260 (3) | 0.0352 (3) | 0.0247 (3) | 0.0029 (2) | 0.0028 (2) | -0.0088 (2) |
| Cl5 | 0.0122 (2) | 0.0257 (3) | 0.0552 (4) | 0.00074 (17) | 0.0044 (2) | -0.0061 (2) |
| Cl6 | 0.0146 (2) | 0.0328 (3) | 0.0441 (3) | 0.00476 (19) | 0.0050 (2) | -0.0047 (2) |
| Cl7 | 0.0361 (3) | 0.0376 (3) | 0.0247 (3) | 0.0111 (2) | 0.0056 (2) | -0.0035 (2) |
| Cl8 | 0.0405 (3) | 0.0313 (3) | 0.0227 (3) | 0.0109 (2) | -0.0003 (2) | -0.0042 (2) |
| Cl9 | 0.0202 (2) | 0.0322 (3) | 0.0496 (4) | 0.0028 (2) | 0.0109 (2) | -0.0070 (2) |
| Cl10 | 0.0338 (3) | 0.0374 (3) | 0.0311 (3) | 0.0046 (2) | -0.0052 (2) | 0.0099 (2) |
| Cl11 | 0.0333 (3) | 0.0369 (3) | 0.0400 (3) | -0.0155 (2) | -0.0043 (2) | 0.0062 (2) |
| Cl12 | 0.0452 (4) | 0.0402 (3) | 0.0277 (3) | 0.0065 (3) | -0.0041 (3) | 0.0088 (2) |
| F2 | 0.0193 (6) | 0.0280 (6) | 0.0263 (7) | -0.0068 (5) | -0.0009 (5) | 0.0026 (5) |
| F4 | 0.0242 (7) | 0.0340 (7) | 0.0315 (7) | -0.0065 (5) | -0.0099 (5) | 0.0093 (6) |
| F1 | 0.0229 (6) | 0.0358 (7) | 0.0260 (7) | -0.0062 (5) | -0.0061 (5) | 0.0077 (5) |
| F3 | 0.0202 (6) | 0.0307 (7) | 0.0295 (7) | -0.0085 (5) | -0.0030 (5) | 0.0048 (5) |
| C3 | 0.0123 (8) | 0.0263 (10) | 0.0318 (11) | 0.0003 (7) | 0.0012 (8) | -0.0026 (8) |
| C4 | 0.0212 (10) | 0.0348 (11) | 0.0191 (10) | -0.0019 (8) | 0.0004 (8) | -0.0012 (8) |
| C5 | 0.0128 (8) | 0.0274 (10) | 0.0203 (10) | -0.0008 (7) | -0.0016 (7) | -0.0021 (7) |
| C6 | 0.0126 (8) | 0.0237 (9) | 0.0212 (9) | 0.0023 (7) | -0.0007 (7) | -0.0023 (7) |
| C7 | 0.0122 (8) | 0.0247 (10) | 0.0206 (9) | 0.0001 (7) | -0.0003 (7) | -0.0025 (7) |
| C8 | 0.0177 (9) | 0.0287 (11) | 0.0287 (11) | -0.0012 (8) | 0.0032 (8) | -0.0039 (8) |
| C9 | 0.0150 (9) | 0.0245 (10) | 0.0213 (10) | 0.0001 (7) | -0.0003 (7) | -0.0017 (7) |
| C10 | 0.0151 (8) | 0.0248 (10) | 0.0207 (10) | 0.0009 (7) | -0.0028 (7) | -0.0011 (7) |
| C11 | 0.0143 (9) | 0.0275 (10) | 0.0199 (9) | -0.0011 (7) | -0.0011 (7) | -0.0020 (7) |
| C12 | 0.0213 (9) | 0.0282 (10) | 0.0189 (9) | -0.0009 (8) | -0.0008 (7) | -0.0024 (8) |
| C13 | 0.0179 (9) | 0.0260 (10) | 0.0206 (10) | -0.0013 (7) | -0.0006 (7) | 0.0018 (8) |
| C14 | 0.0130 (8) | 0.0325 (11) | 0.0238 (10) | -0.0029 (7) | -0.0001 (7) | -0.0021 (8) |
| C15 | 0.0150 (9) | 0.0300 (10) | 0.0203 (10) | -0.0015 (7) | 0.0003 (7) | -0.0044 (8) |
| C16 | 0.0146 (9) | 0.0238 (9) | 0.0230 (10) | -0.0020 (7) | -0.0004 (7) | 0.0009 (7) |
| C17 | 0.0176 (10) | 0.0238 (10) | 0.0367 (12) | 0.0006 (8) | 0.0020 (8) | -0.0030 (8) |
| C18 | 0.0148 (8) | 0.0224 (9) | 0.0230 (10) | 0.0006 (7) | -0.0011 (7) | -0.0015 (7) |
| C19 | 0.0159 (9) | 0.0314 (11) | 0.0200 (10) | 0.0018 (8) | -0.0001 (7) | 0.0019 (8) |
| C20 | 0.0233 (10) | 0.0272 (10) | 0.0284 (11) | 0.0041 (8) | -0.0011 (8) | 0.0011 (8) |
| C21 | 0.0192 (9) | 0.0296 (11) | 0.0275 (11) | -0.0009 (8) | -0.0047 (8) | 0.0046 (8) |
| C22 | 0.0259 (10) | 0.0284 (11) | 0.0239 (11) | 0.0025 (8) | 0.0012 (8) | 0.0000 (8) |
| C23 | 0.0152 (9) | 0.0234 (9) | 0.0250 (10) | -0.0025 (7) | 0.0011 (7) | 0.0005 (8) |
| C24 | 0.0175 (9) | 0.0348 (11) | 0.0202 (10) | -0.0016 (8) | -0.0022 (7) | 0.0008 (8) |
| C25 | 0.0243 (10) | 0.0323 (11) | 0.0231 (11) | 0.0010 (8) | -0.0006 (8) | 0.0035 (8) |
| C26 | 0.0210 (10) | 0.0268 (10) | 0.0227 (10) | 0.0021 (8) | -0.0010 (8) | -0.0018 (8) |
| C27 | 0.0179 (9) | 0.0269 (10) | 0.0245 (10) | -0.0042 (8) | 0.0007 (8) | -0.0017 (8) |
| C28 | 0.0147 (9) | 0.0374 (12) | 0.0250 (11) | -0.0029 (8) | -0.0005 (8) | 0.0043 (9) |
| C29 | 0.0135 (9) | 0.0284 (10) | 0.0218 (10) | 0.0010 (7) | 0.0001 (7) | -0.0026 (8) |
| C30 | 0.0147 (8) | 0.0244 (9) | 0.0207 (9) | 0.0006 (7) | 0.0006 (7) | -0.0020 (7) |
| C31 | 0.0179 (9) | 0.0241 (10) | 0.0228 (10) | -0.0012 (7) | -0.0015 (8) | 0.0029 (8) |
| C32 | 0.0274 (11) | 0.0279 (11) | 0.0290 (11) | -0.0029 (9) | 0.0030 (9) | -0.0053 (9) |
| C33 | 0.0145 (9) | 0.0292 (10) | 0.0229 (10) | 0.0002 (7) | 0.0002 (7) | -0.0019 (8) |
| C34 | 0.0245 (10) | 0.0339 (12) | 0.0219 (10) | -0.0095 (9) | -0.0035 (8) | 0.0017 (8) |
| C36 | 0.068 (3) | 0.074 (3) | 0.086 (3) | 0.007 (2) | 0.009 (2) | 0.015 (2) |
| C37 | 0.050 (2) | 0.059 (2) | 0.093 (3) | 0.0043 (17) | -0.011 (2) | 0.022 (2) |
| C38 | 0.072 (3) | 0.066 (3) | 0.091 (3) | 0.010 (2) | 0.000 (3) | -0.008 (2) |
| C40 | 0.052 (2) | 0.053 (2) | 0.154 (6) | 0.0164 (18) | -0.022 (3) | -0.010 (3) |
| C41 | 0.133 (5) | 0.117 (4) | 0.061 (3) | 0.084 (4) | 0.036 (3) | 0.023 (3) |
| C42 | 0.081 (3) | 0.089 (4) | 0.083 (3) | 0.000 (3) | 0.000 (3) | 0.020 (3) |

*Geometric parameters (Å, º)*

| Cl1—C12 | 1.735 (2) | C9—C30 | 1.382 (3) |  |
| --- | --- | --- | --- | --- |
| Cl2—C27 | 1.730 (2) | C9—C13 | 1.443 (3) |  |
| Cl3—C19 | 1.729 (2) | C9—C23 | 1.444 (3) |  |
| Cl4—C15 | 1.727 (2) | C10—C26 | 1.402 (3) |  |
| Cl5—C3 | 1.733 (2) | C10—C33 | 1.402 (3) |  |
| Cl6—C29 | 1.728 (2) | C11—C19 | 1.399 (3) |  |
| Cl7—C33 | 1.733 (2) | C11—C30 | 1.488 (3) |  |
| Cl8—C26 | 1.735 (2) | C12—C22 | 1.389 (3) |  |
| Cl9—C8 | 1.725 (2) | C13—C16 | 1.353 (3) |  |
| Cl10—C21 | 1.734 (2) | C14—C27 | 1.379 (3) |  |
| Cl11—C34 | 1.730 (2) | C14—C29 | 1.387 (3) |  |
| Cl12—C25 | 1.735 (2) | C16—C18 | 1.445 (3) |  |
| F2—C16 | 1.345 (2) | C17—C27 | 1.387 (3) |  |
| F4—C31 | 1.344 (3) | C18—C31 | 1.443 (3) |  |
| F1—C13 | 1.339 (3) | C19—C28 | 1.382 (3) |  |
| F3—C23 | 1.341 (2) | C20—C21 | 1.385 (3) |  |
| C3—C17 | 1.381 (3) | C20—C26 | 1.389 (3) |  |
| C3—C7 | 1.403 (3) | C21—C24 | 1.379 (3) |  |
| C4—C25 | 1.383 (3) | C22—C25 | 1.383 (3) |  |
| C4—C15 | 1.388 (3) | C23—C31 | 1.353 (3) |  |
| C5—C12 | 1.407 (3) | C24—C33 | 1.388 (3) |  |
| C5—C15 | 1.412 (3) | C28—C34 | 1.376 (4) |  |
| C5—C30 | 1.491 (3) | C32—C34 | 1.383 (3) |  |
| C6—C18 | 1.382 (3) | C36—C37 | 1.5381 (10) |  |
| C6—C7 | 1.482 (3) | C36—C42 | 1.5404 (11) |  |
| C6—C10 | 1.487 (3) | C37—C38 | 1.5370 (11) |  |
| C7—C29 | 1.408 (3) | C38—C40 | 1.5400 (10) |  |
| C8—C32 | 1.386 (3) | C40—C41 | 1.5376 (11) |  |
| C8—C11 | 1.400 (3) |  |  |  |
|  |  |  |  |  |
| C17—C3—C7 | 123.34 (19) | C28—C19—C11 | 123.5 (2) |  |
| C17—C3—Cl5 | 116.08 (17) | C28—C19—Cl3 | 116.88 (17) |  |
| C7—C3—Cl5 | 120.54 (17) | C11—C19—Cl3 | 119.39 (17) |  |
| C25—C4—C15 | 118.2 (2) | C21—C20—C26 | 118.4 (2) |  |
| C12—C5—C15 | 115.2 (2) | C24—C21—C20 | 121.8 (2) |  |
| C12—C5—C30 | 122.84 (19) | C24—C21—Cl10 | 118.79 (18) |  |
| C15—C5—C30 | 121.82 (19) | C20—C21—Cl10 | 119.43 (19) |  |
| C18—C6—C7 | 121.34 (19) | C25—C22—C12 | 118.9 (2) |  |
| C18—C6—C10 | 121.57 (19) | F3—C23—C31 | 116.85 (19) |  |
| C7—C6—C10 | 117.03 (17) | F3—C23—C9 | 118.60 (18) |  |
| C3—C7—C29 | 114.97 (19) | C31—C23—C9 | 124.2 (2) |  |
| C3—C7—C6 | 121.41 (18) | C21—C24—C33 | 118.2 (2) |  |
| C29—C7—C6 | 123.53 (19) | C22—C25—C4 | 121.5 (2) |  |
| C32—C8—C11 | 123.0 (2) | C22—C25—Cl12 | 119.17 (19) |  |
| C32—C8—Cl9 | 116.51 (18) | C4—C25—Cl12 | 119.29 (18) |  |
| C11—C8—Cl9 | 120.47 (17) | C20—C26—C10 | 122.7 (2) |  |
| C30—C9—C13 | 123.3 (2) | C20—C26—Cl8 | 117.17 (18) |  |
| C30—C9—C23 | 124.3 (2) | C10—C26—Cl8 | 120.11 (17) |  |
| C13—C9—C23 | 112.40 (18) | C14—C27—C17 | 121.0 (2) |  |
| C26—C10—C33 | 115.8 (2) | C14—C27—Cl2 | 120.49 (17) |  |
| C26—C10—C6 | 121.70 (19) | C17—C27—Cl2 | 118.51 (18) |  |
| C33—C10—C6 | 122.52 (19) | C34—C28—C19 | 118.3 (2) |  |
| C19—C11—C8 | 115.2 (2) | C14—C29—C7 | 123.2 (2) |  |
| C19—C11—C30 | 121.7 (2) | C14—C29—Cl6 | 116.56 (16) |  |
| C8—C11—C30 | 123.12 (19) | C7—C29—Cl6 | 120.19 (17) |  |
| C22—C12—C5 | 122.8 (2) | C9—C30—C11 | 122.40 (19) |  |
| C22—C12—Cl1 | 116.34 (18) | C9—C30—C5 | 122.19 (19) |  |
| C5—C12—Cl1 | 120.89 (17) | C11—C30—C5 | 115.37 (17) |  |
| F1—C13—C16 | 117.95 (19) | F4—C31—C23 | 117.40 (19) |  |
| F1—C13—C9 | 118.86 (18) | F4—C31—C18 | 118.81 (18) |  |
| C16—C13—C9 | 123.1 (2) | C23—C31—C18 | 123.3 (2) |  |
| C27—C14—C29 | 118.67 (19) | C34—C32—C8 | 118.5 (2) |  |
| C4—C15—C5 | 123.3 (2) | C24—C33—C10 | 123.2 (2) |  |
| C4—C15—Cl4 | 116.72 (17) | C24—C33—Cl7 | 116.49 (17) |  |
| C5—C15—Cl4 | 119.88 (17) | C10—C33—Cl7 | 120.32 (17) |  |
| F2—C16—C13 | 117.26 (19) | C28—C34—C32 | 121.4 (2) |  |
| F2—C16—C18 | 118.19 (18) | C28—C34—Cl11 | 119.36 (19) |  |
| C13—C16—C18 | 124.4 (2) | C32—C34—Cl11 | 119.3 (2) |  |
| C3—C17—C27 | 118.8 (2) | C37—C36—C42 | 111.1 (3) |  |
| C6—C18—C31 | 123.6 (2) | C38—C37—C36 | 108.95 (10) |  |
| C6—C18—C16 | 124.2 (2) | C37—C38—C40 | 108.7 (3) |  |
| C31—C18—C16 | 112.21 (18) | C41—C40—C38 | 108.70 (10) |  |
|  |  |  |  |  |
| C17—C3—C7—C29 | -1.1 (3) | C12—C22—C25—C4 | 1.2 (4) |  |
| Cl5—C3—C7—C29 | 176.55 (17) | C12—C22—C25—Cl12 | 179.78 (18) |  |
| C17—C3—C7—C6 | -177.8 (2) | C15—C4—C25—C22 | -1.1 (4) |  |
| Cl5—C3—C7—C6 | -0.1 (3) | C15—C4—C25—Cl12 | -179.65 (17) |  |
| C18—C6—C7—C3 | -51.9 (3) | C21—C20—C26—C10 | -0.6 (4) |  |
| C10—C6—C7—C3 | 125.4 (2) | C21—C20—C26—Cl8 | -179.20 (18) |  |
| C18—C6—C7—C29 | 131.7 (2) | C33—C10—C26—C20 | 0.6 (3) |  |
| C10—C6—C7—C29 | -51.0 (3) | C6—C10—C26—C20 | -178.4 (2) |  |
| C18—C6—C10—C26 | -59.6 (3) | C33—C10—C26—Cl8 | 179.19 (16) |  |
| C7—C6—C10—C26 | 123.1 (2) | C6—C10—C26—Cl8 | 0.2 (3) |  |
| C18—C6—C10—C33 | 121.6 (2) | C29—C14—C27—C17 | -0.1 (4) |  |
| C7—C6—C10—C33 | -55.8 (3) | C29—C14—C27—Cl2 | -179.32 (17) |  |
| C32—C8—C11—C19 | -1.0 (3) | C3—C17—C27—C14 | -1.4 (4) |  |
| Cl9—C8—C11—C19 | 178.43 (17) | C3—C17—C27—Cl2 | 177.88 (19) |  |
| C32—C8—C11—C30 | 177.4 (2) | C11—C19—C28—C34 | -1.7 (3) |  |
| Cl9—C8—C11—C30 | -3.2 (3) | Cl3—C19—C28—C34 | 173.22 (18) |  |
| C15—C5—C12—C22 | -2.8 (3) | C27—C14—C29—C7 | 1.0 (3) |  |
| C30—C5—C12—C22 | 173.4 (2) | C27—C14—C29—Cl6 | -177.17 (18) |  |
| C15—C5—C12—Cl1 | 177.67 (16) | C3—C7—C29—C14 | -0.4 (3) |  |
| C30—C5—C12—Cl1 | -6.2 (3) | C6—C7—C29—C14 | 176.2 (2) |  |
| C30—C9—C13—F1 | 11.2 (3) | C3—C7—C29—Cl6 | 177.70 (17) |  |
| C23—C9—C13—F1 | -171.35 (19) | C6—C7—C29—Cl6 | -5.7 (3) |  |
| C30—C9—C13—C16 | -171.9 (2) | C13—C9—C30—C11 | -168.1 (2) |  |
| C23—C9—C13—C16 | 5.6 (3) | C23—C9—C30—C11 | 14.8 (3) |  |
| C25—C4—C15—C5 | -1.1 (3) | C13—C9—C30—C5 | 14.5 (3) |  |
| C25—C4—C15—Cl4 | 175.60 (18) | C23—C9—C30—C5 | -162.6 (2) |  |
| C12—C5—C15—C4 | 3.0 (3) | C19—C11—C30—C9 | -120.5 (2) |  |
| C30—C5—C15—C4 | -173.3 (2) | C8—C11—C30—C9 | 61.2 (3) |  |
| C12—C5—C15—Cl4 | -173.65 (16) | C19—C11—C30—C5 | 57.0 (3) |  |
| C30—C5—C15—Cl4 | 10.1 (3) | C8—C11—C30—C5 | -121.2 (2) |  |
| F1—C13—C16—F2 | -1.9 (3) | C12—C5—C30—C9 | 59.5 (3) |  |
| C9—C13—C16—F2 | -178.80 (19) | C15—C5—C30—C9 | -124.6 (2) |  |
| F1—C13—C16—C18 | 173.7 (2) | C12—C5—C30—C11 | -118.0 (2) |  |
| C9—C13—C16—C18 | -3.2 (4) | C15—C5—C30—C11 | 57.9 (3) |  |
| C7—C3—C17—C27 | 2.0 (4) | F3—C23—C31—F4 | -0.6 (3) |  |
| Cl5—C3—C17—C27 | -175.76 (19) | C9—C23—C31—F4 | -174.0 (2) |  |
| C7—C6—C18—C31 | 157.7 (2) | F3—C23—C31—C18 | 171.6 (2) |  |
| C10—C6—C18—C31 | -19.5 (3) | C9—C23—C31—C18 | -1.8 (4) |  |
| C7—C6—C18—C16 | -20.5 (3) | C6—C18—C31—F4 | -2.0 (3) |  |
| C10—C6—C18—C16 | 162.3 (2) | C16—C18—C31—F4 | 176.35 (19) |  |
| F2—C16—C18—C6 | -7.9 (3) | C6—C18—C31—C23 | -174.1 (2) |  |
| C13—C16—C18—C6 | 176.5 (2) | C16—C18—C31—C23 | 4.3 (3) |  |
| F2—C16—C18—C31 | 173.71 (18) | C11—C8—C32—C34 | -0.6 (4) |  |
| C13—C16—C18—C31 | -1.9 (3) | Cl9—C8—C32—C34 | 179.96 (18) |  |
| C8—C11—C19—C28 | 2.2 (3) | C21—C24—C33—C10 | 0.1 (3) |  |
| C30—C11—C19—C28 | -176.2 (2) | C21—C24—C33—Cl7 | -178.06 (17) |  |
| C8—C11—C19—Cl3 | -172.58 (17) | C26—C10—C33—C24 | -0.3 (3) |  |
| C30—C11—C19—Cl3 | 9.0 (3) | C6—C10—C33—C24 | 178.6 (2) |  |
| C26—C20—C21—C24 | 0.2 (4) | C26—C10—C33—Cl7 | 177.71 (16) |  |
| C26—C20—C21—Cl10 | 178.77 (18) | C6—C10—C33—Cl7 | -3.3 (3) |  |
| C5—C12—C22—C25 | 0.8 (4) | C19—C28—C34—C32 | -0.1 (4) |  |
| Cl1—C12—C22—C25 | -179.61 (19) | C19—C28—C34—Cl11 | -178.79 (17) |  |
| C30—C9—C23—F3 | 1.0 (3) | C8—C32—C34—C28 | 1.2 (4) |  |
| C13—C9—C23—F3 | -176.46 (19) | C8—C32—C34—Cl11 | 179.86 (19) |  |
| C30—C9—C23—C31 | 174.3 (2) | C42—C36—C37—C38 | 176.3 (4) |  |
| C13—C9—C23—C31 | -3.2 (3) | C36—C37—C38—C40 | -171.4 (4) |  |
| C20—C21—C24—C33 | 0.0 (3) | C37—C38—C40—C41 | -177.3 (4) |  |
| Cl10—C21—C24—C33 | -178.54 (17) |  |  |  |

**Table S11:** Hydrogen-bond geometry (Å, º) for **Cl,F-TTH**.

| *D*—H···*A* | *D*—H | H···*A* | *D*···*A* | *D*—H···*A* |
| --- | --- | --- | --- | --- |
| C4—H4···Cl3^i^ | 0.93 | 2.94 | 3.844 (2) | 163 |
| C14—H14···Cl2^ii^ | 0.93 | 2.93 | 3.849 (2) | 171 |
| C17—H17···Cl5^iii^ | 0.93 | 2.76 | 3.646 (2) | 160 |

Symmetry codes: (i) *x*, -*y*+1/2, *z*+1/2; (ii) -*x*, -*y*, -*z*; (iii) -*x*+1, -*y*, -*z*.

#

# Computational details

Quantum chemical calculations were carried out using density functional theory (DFT) to investigate the equilibrium ground state structure of **F,Cl-TTH** and **Cl,F-TTH** and time-dependent DFT (TD-DFT) for the bright excited state SE. Singlet spin ground state geometries were determined at restricted (RDFT) level, employing the M062X/def2SVP with the Grimme dispersion contribution D3.^23,24^ A stable closed-shell (CS) structure, i.e. no open-shell unrestricted DFT solution was found, thereby supporting the experimentally reported quinoidal structures. The electronic absorption spectra of **TTH** derivatives were obtained by TD-M062X-D3/def2-SVP calculations (Table S12) and are shown in Figure S54. The main absorption band is assigned to the state dominated by the H→L excitation (SE state). The overestimate of its excitation energy is typical of the functional employed. The equilibrium structure of the SE state was then optimized at TD-M062X-D3/def2SVP level for both derivatives. Two conformers were considered for **F,Cl-TTH**, belonging to the C_s_ and C_2_ symmetry, respectively. The lowest energy ground state structure resulted to be the C_2_ symmetry, in agreement with the experimentally determined crystal structure. An additional structure, characterized by an almost planar central core, was determined for **F,Cl-TTH** (Figure S55). Vibrational frequency calculations show that this structure is a saddle point that lies ca. 7.4 kcal/mol above the C_2_ minimum. In addition to **TTH** derivatives, also the two folded **pQDM_1** and **pQDM_2** were optimized in their ground electronic state. A graphical representation of optimized ground and excited state geometries is collected in Figures S46-S53. The computed charge distribution, in the ground and excited states, was analysed to determine the quadrupolar character of **F,Cl-TTH** and **Cl,F-TTH**. The fragment definition adopted for the quadrupolar analysis is presented in Figure S56 and the fragment charges are collected in Table S15.

Because conjugated diradicals are characterized by low-lying excited states dominated by doubly excited configurations, and the latter are not included in TD-DFT calculations, we used multireference methods to assess the role of static electron correlation and multiple excitations on the sequence of low-lying excited electronic states. Thus, beside DFT and TD-DFT calculations, the excitation energies were determined with the complete active space self-consistent field (CASSCF)^25^ calculations, followed by NEVPT2^26,27^ or CASPT2 corrections to include dynamical correlation. The calculations were carried out with an active space including 10 electrons in 10 orbitals CASSCF(10,10) and 14 electrons in 14 orbitals CASSCF(14,14). The optimized geometry of the bright excited state was employed in these calculations (Table S13). All CASSCF calculations were performed with ORCA 6.0.1^28,29^ using the def2-SVP basis set.^30^ The resolution of identity approximation and the related basis sets for both Coulomb and HF exchange integrals were used (RI-JK).^31^ Four roots were calculated for the state averaged calculations of the singlet excited states.

**Diradical character** $\boldsymbol{y}_{\boldsymbol{0}}$**.**

The most common descriptor of diradical character is the $y_{0}$ index which assumes values between 0=no diradical character and 1=full diradical. $y_{0}$ can be determined at UHF level or using UDFT but the corresponding values can be rather different and functional dependent.^32^ For this reason in this work the $y_{0}$ index was computed at PUHF/def2SVP level (at the computed equilibrium structures), following Yamaguchi’s approach in the spin-projection scheme as^33^:

| $y_{0}^{PUHF}=1-\frac{2T_{0}}{1+T_{0}^{2}}$ | (1) |
| --- | --- |

with T_0_ calculated as:

| $T_{0}=\frac{n_{HONO}- n_{LUNO}}{2}$ | (2) |
| --- | --- |

where $n_{HONO}$ and $n_{LUNO}$ are the occupation number of the highest occupied (HONO) and lowest unoccupied (LUNO) natural orbitals, respectively. All the above calculations were carried out with the Gaussian 16 package.^34^ The computed $y_{0}$ values, for the investigated diradicaloids in their singlet ground electronic state are collected in Table S12.

**Table S12.** TD-M062X-D3/def2SVP vertical excitation energies for the SE state, computed at the M062X-D3/def2SVP optimized geometry of the ground state. The last column contains the computed diradical character $y_{0}$ (UHF/def2SVP calculations at the M062X-D3/def2SVP optimized ground state geometries.

| Excited State | TD-M062X-D3  ΔE(SE-GR) / eV (nm) | Exp / eV (nm) | $y_{0}\left( PUHF \right)$ |
| --- | --- | --- | --- |
| **TTH** | 2.80 (443) | 2.49 (498) | 0.315 |
| **F,Cl-TTH (C_s_)** | 3.57 (347) |  | 0.133 |
| **F,Cl-TTH (C_2_)** | 3.81 (325) | 3.58 (346) | 0.093 |
| **Cl,F-TTH** | 3.02 (410) | 2.82 (440) | 0.275 |

**Table S13.** Excitation energies of the SE and DE states computed at CAS+NEVPT2 level (at the TDDFT optimized geometry of the SE state) along with the energy difference between the two excited states.

| Excited State | CAS(10,10)+NEVPT2  ΔE(SE-GR)/eV | CAS(10,10)+NEVPT2  ΔE(DE-GR)/eV | CAS(10,10)+NEVPT2  ΔE(DE-SE)/eV |
| --- | --- | --- | --- |
| **TTH** | 1.78 | 1.97 | 0.19 |
| **F,Cl-TTH (C_s_)** | 1.86 | 1.95 | 0.09 |
| **F,Cl-TTH (C_2_)** | 1.84 | 1.97 | 0.13 |
| **Cl,F-TTH** | 2.32 | 2.80 | 0.48 |

**Table S14.** Comparison between computed energy differences between the two excited states (E(DE) -E(SE)) at CAS+NEVPT2 and CASPT2 level, using different active spaces (at the TDDFT optimized geometry of the SE state).

| Excited State | CAS(10,10) + **NEVPT2**  ΔE(DE-SE)/eV | CAS(14,14) + **NEVPT2**  ΔE(DE-SE)/eV | CAS(10,10) + **CASPT2**  ΔE(DE-SE)/eV  IPEA SHIFT=0.25 | CAS(14,14) + **CASPT2**  ΔE(DE-SE)/eV  IPEA SHIFT=0.25 |
| --- | --- | --- | --- | --- |
| **TTH** | 0.19 | 0.18 | -0.01 | 0.10 |
| **F,Cl-TTH (C_s_)** | 0.09 | -0.02 | -0.13 | -0.16 |
| **F,Cl-TTH (C_2_)** | 0.13 | 0.07 | -0.13 | -0.15 |
| **Cl,F-TTH** | 0.48 | 0.42 | 0.24 | 0.17 |


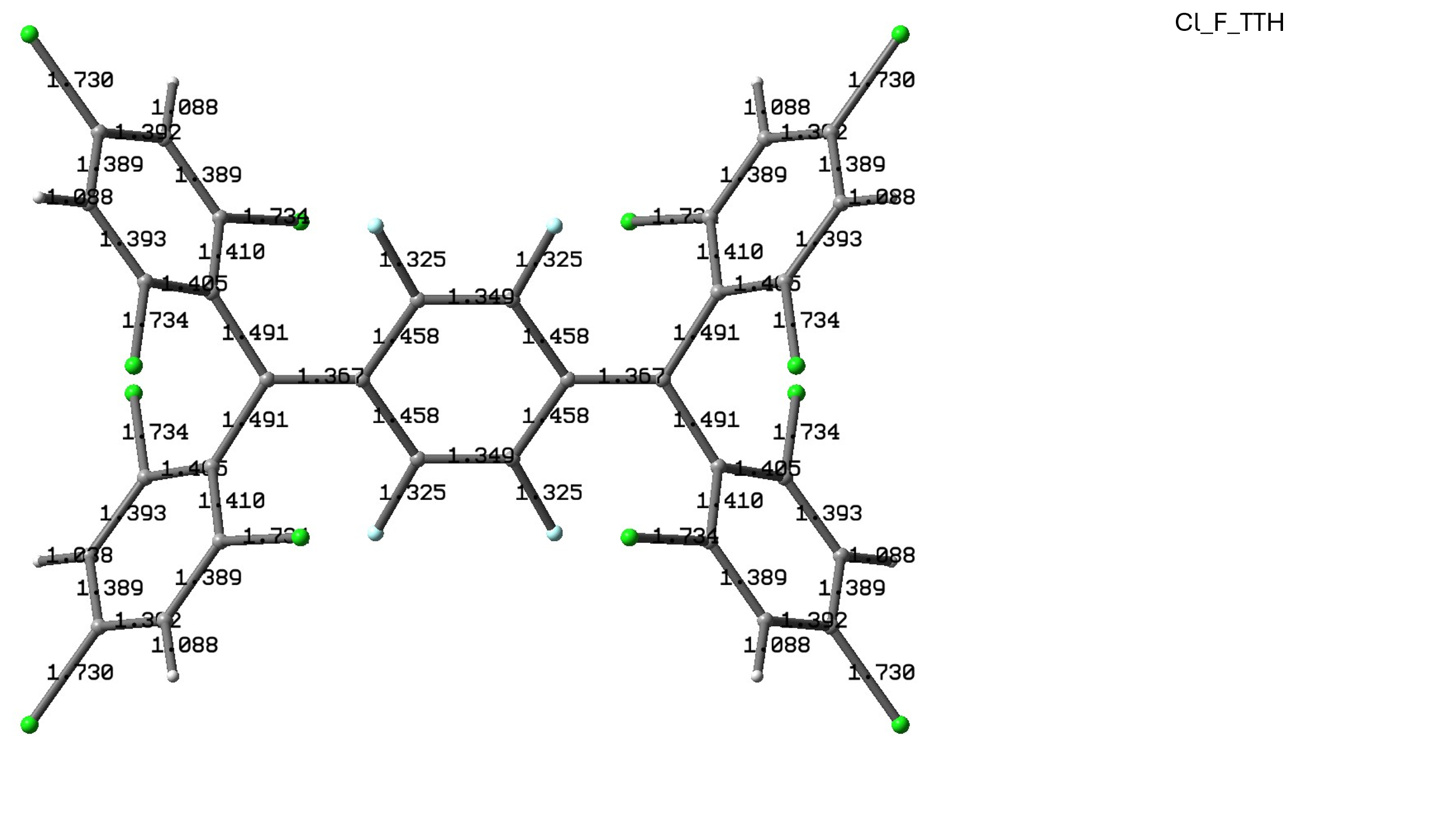

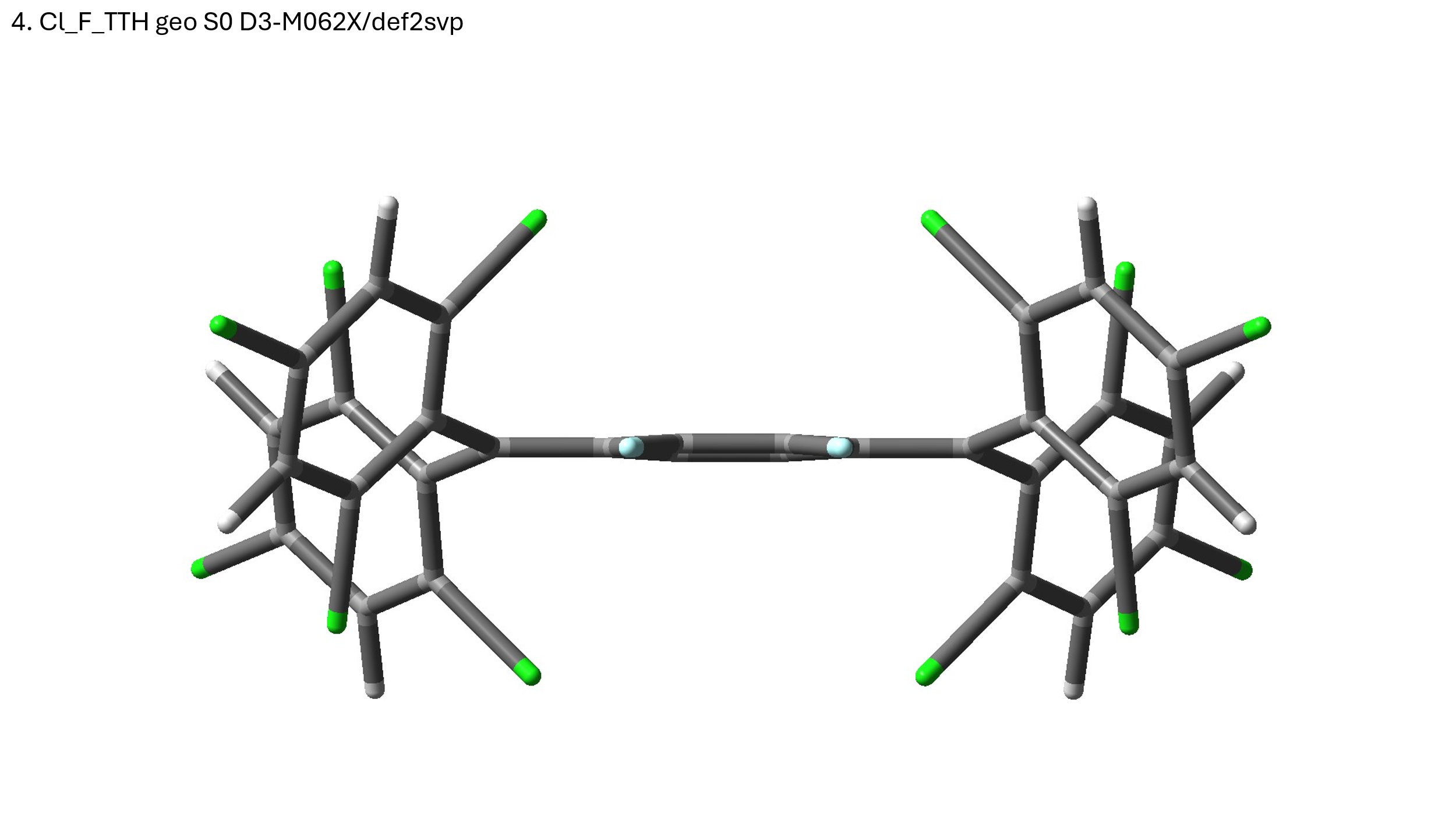


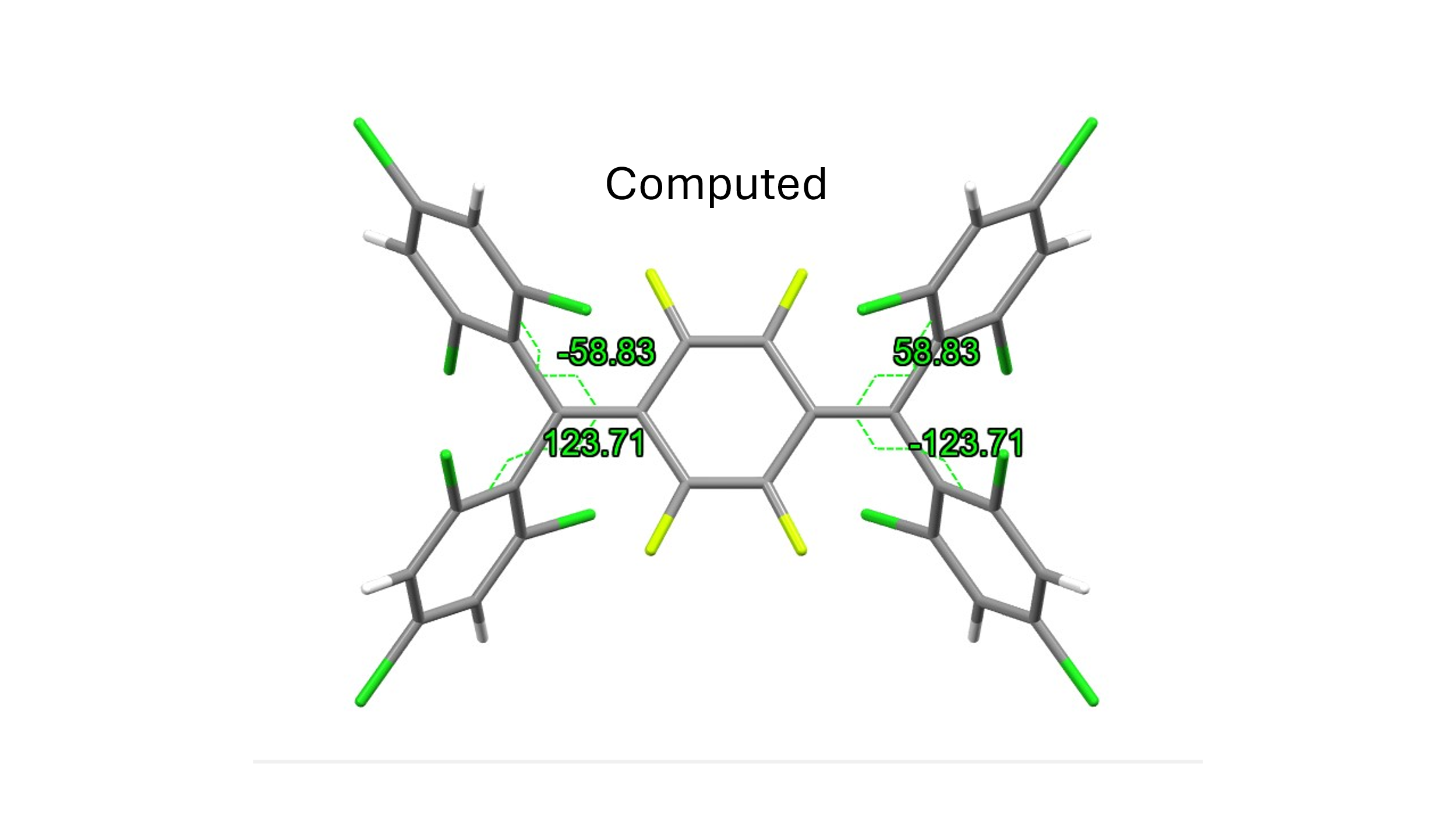

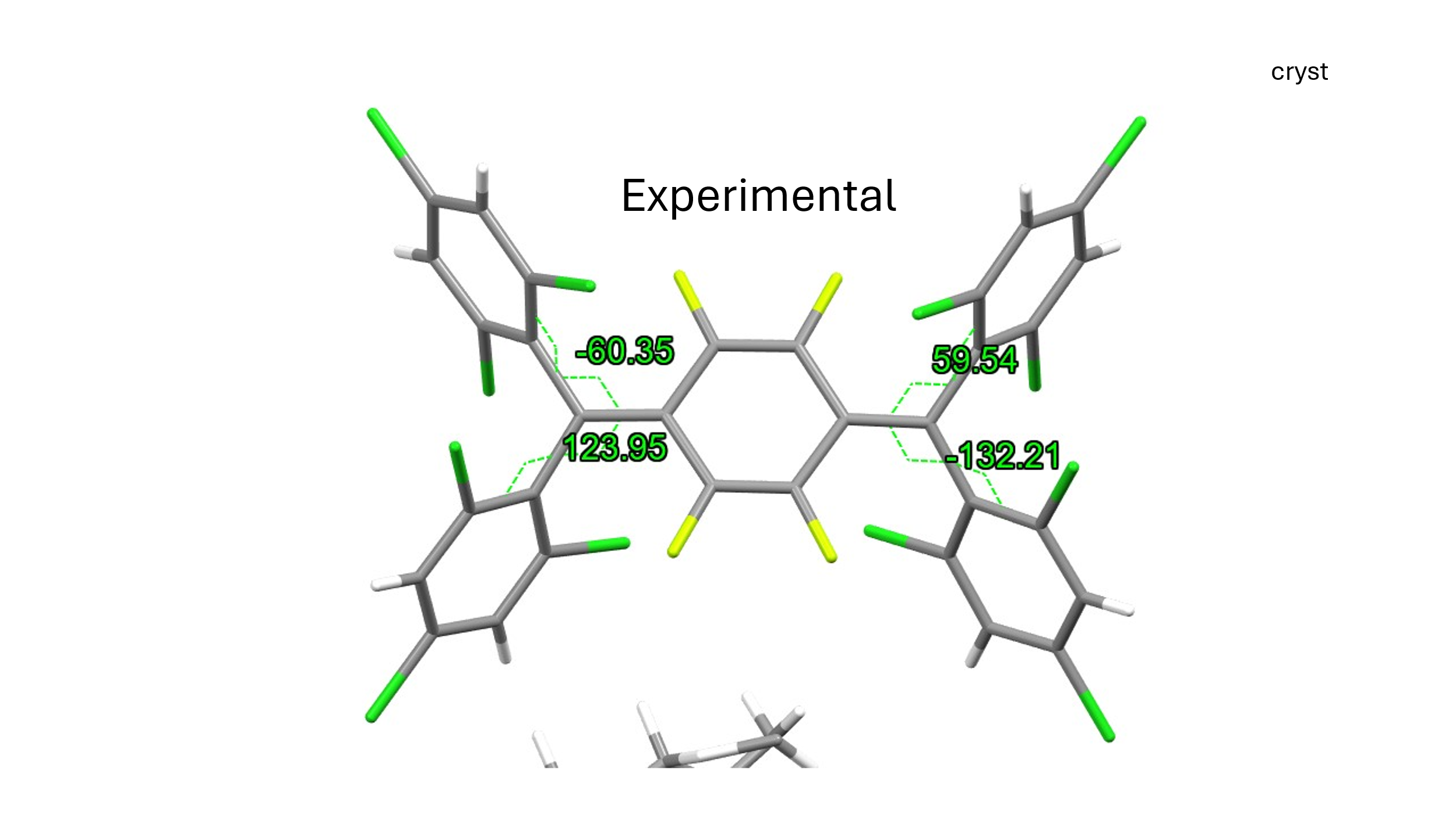


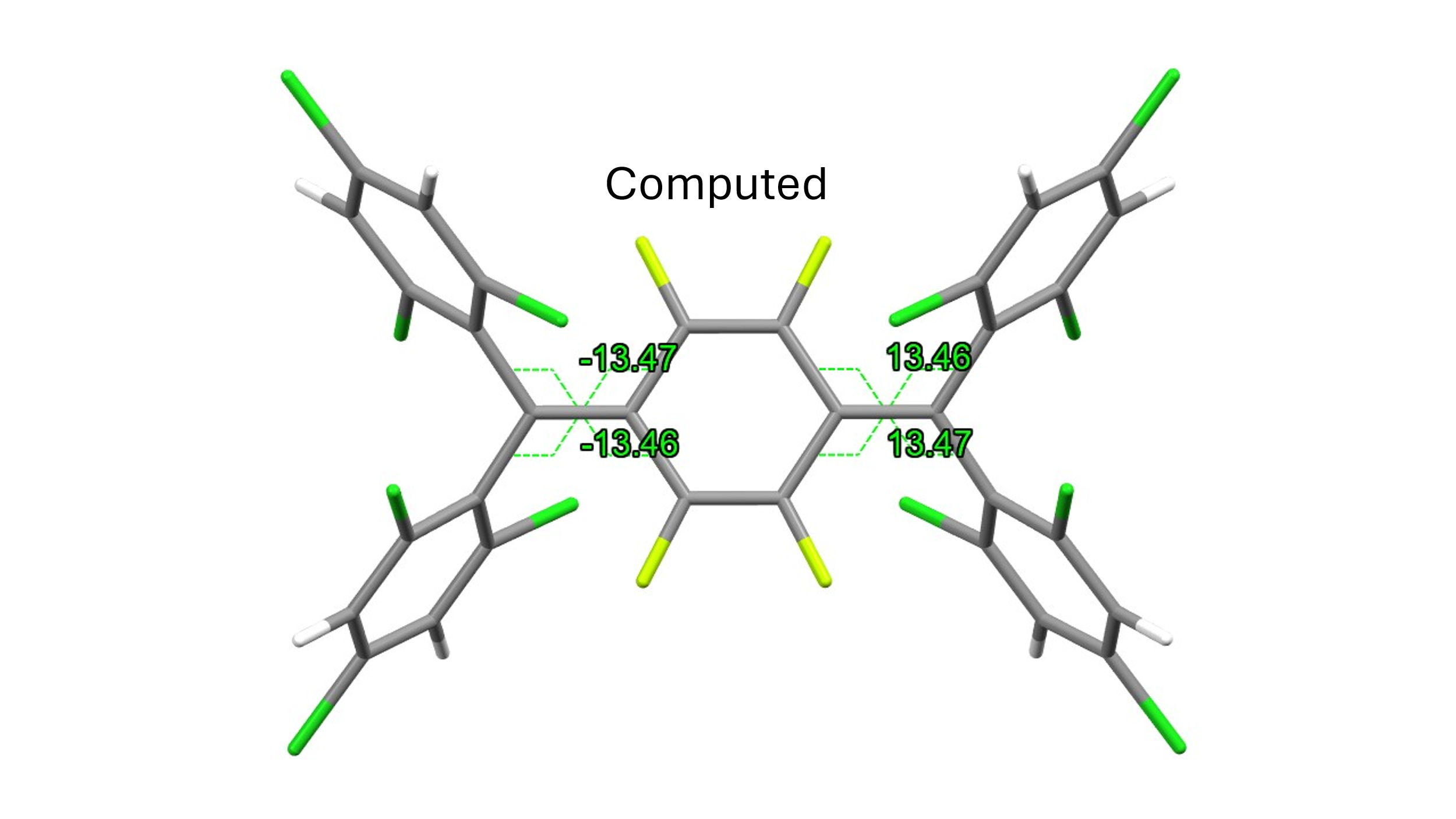

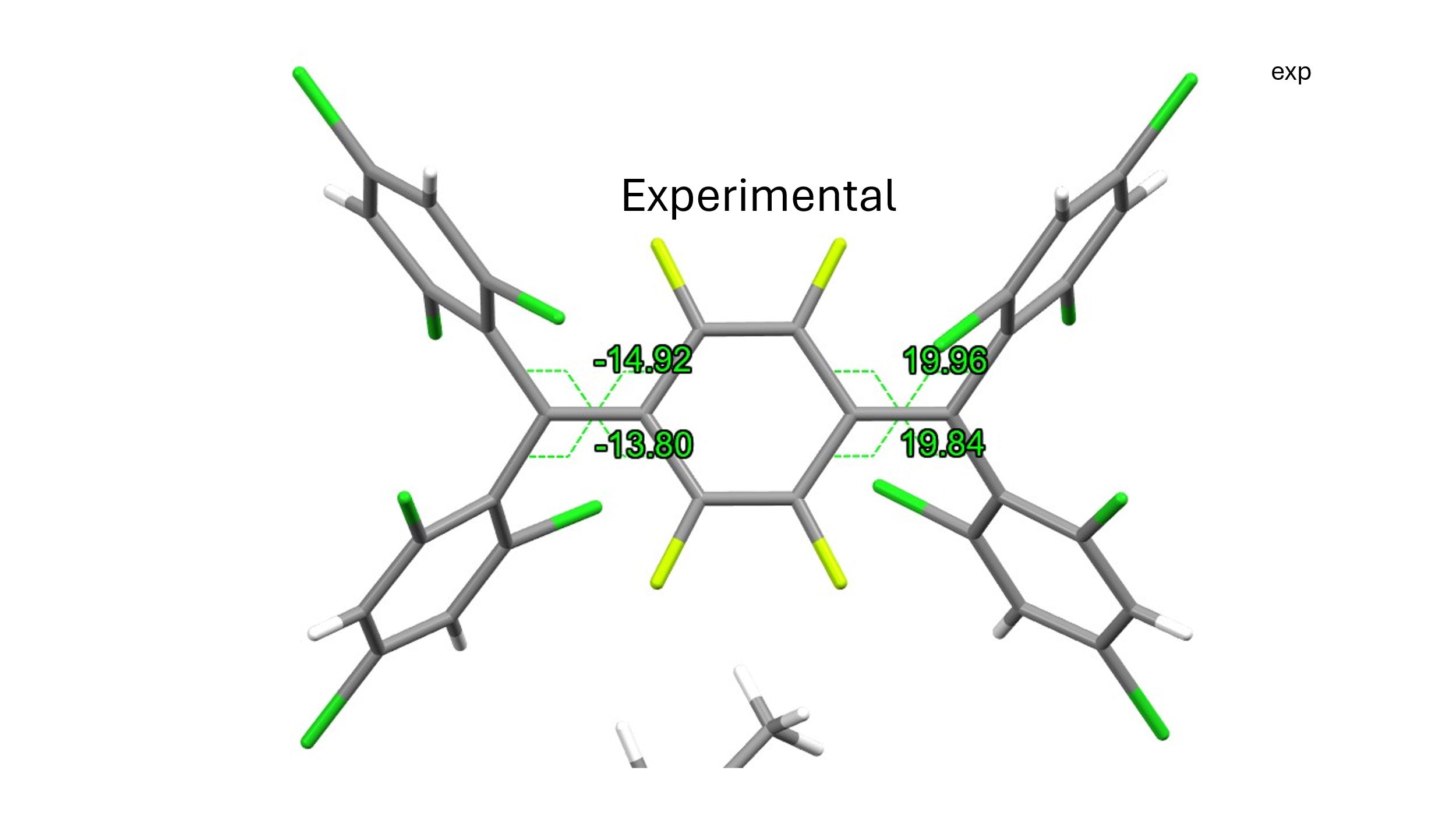


**Figure S46.** Computed bond lengths (Å) of the ground state of **Cl,F-TTH** at M062X-D3/def2SVP level, side view and comparison with the crystal structure.


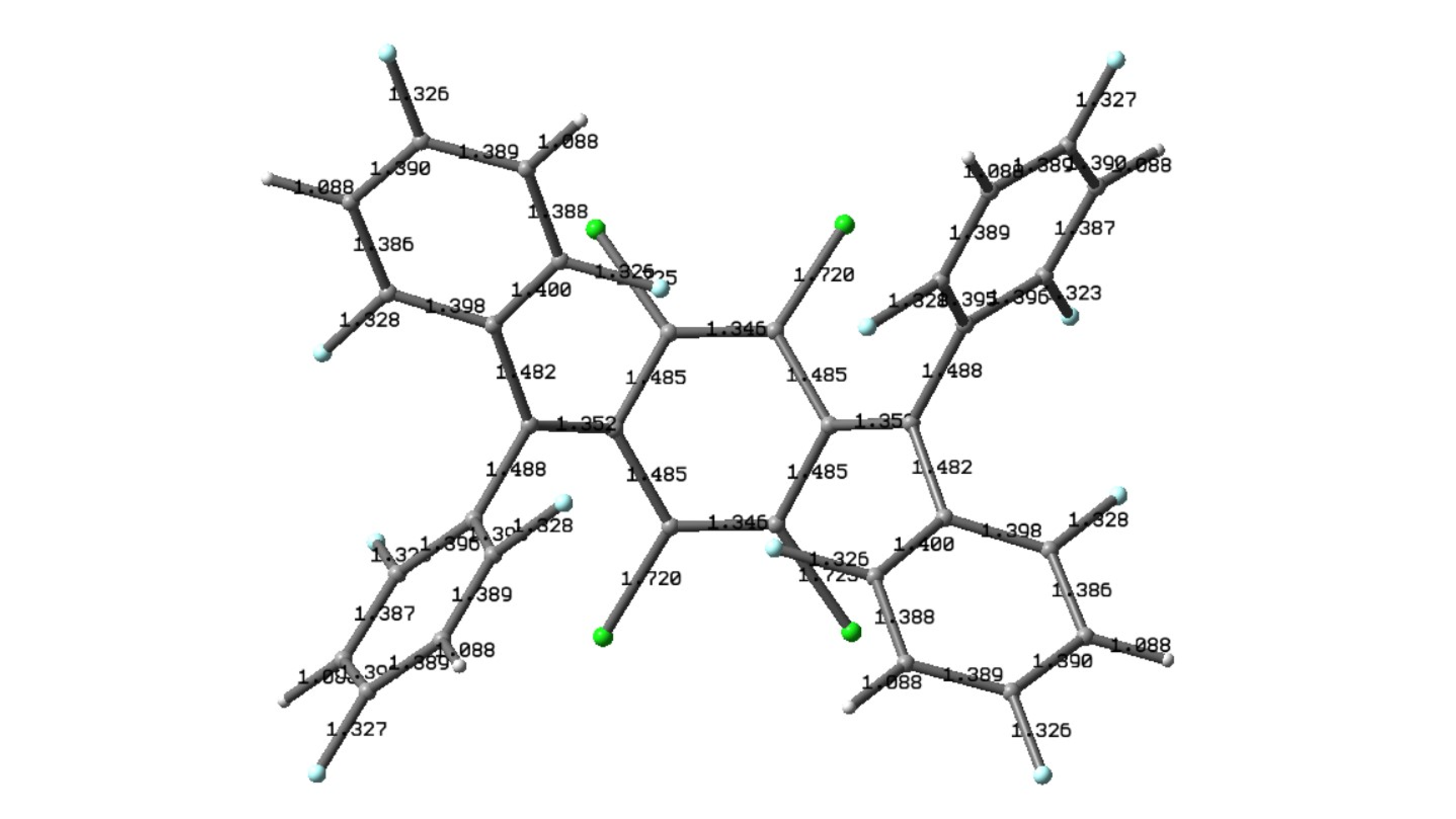

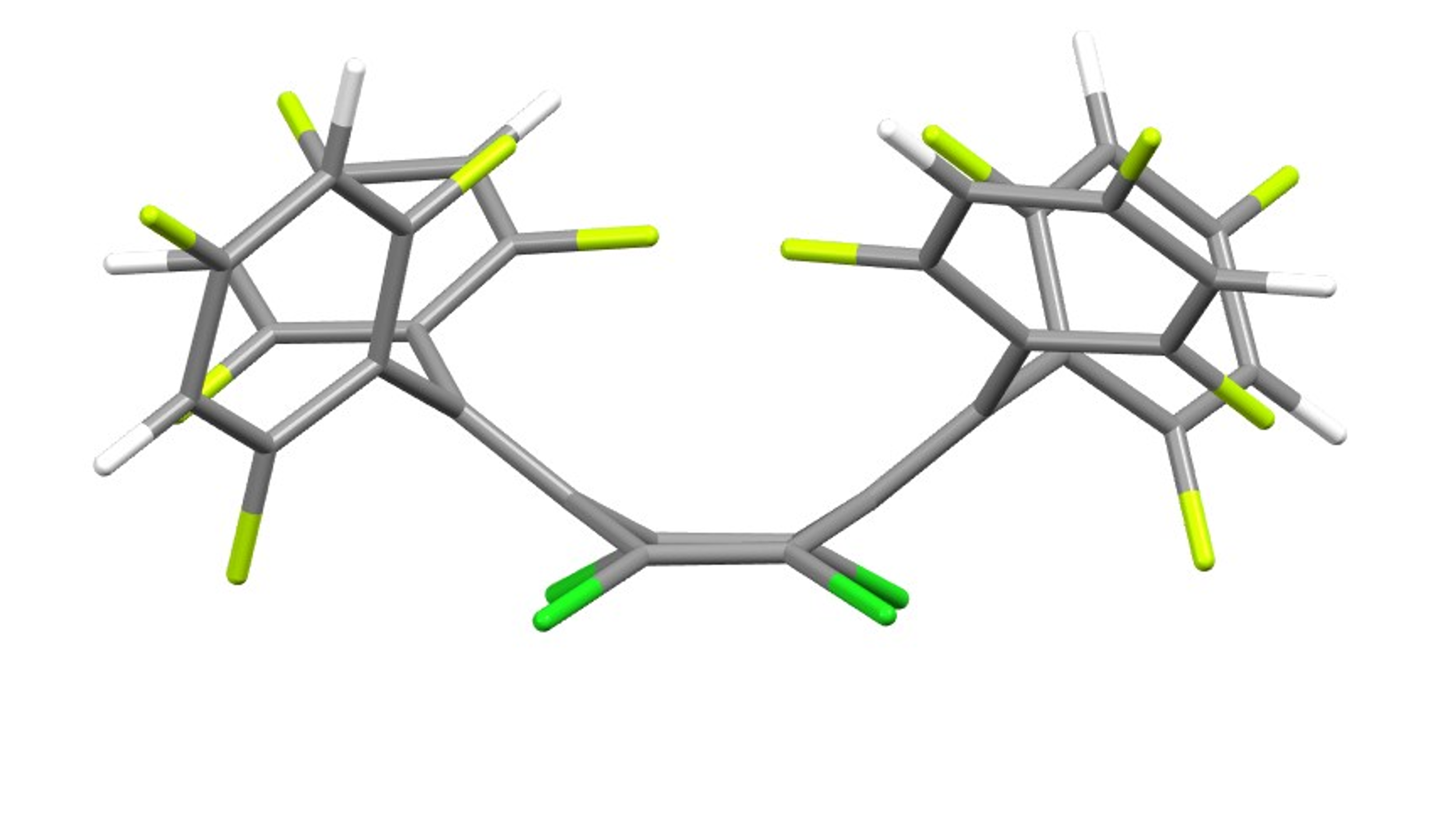


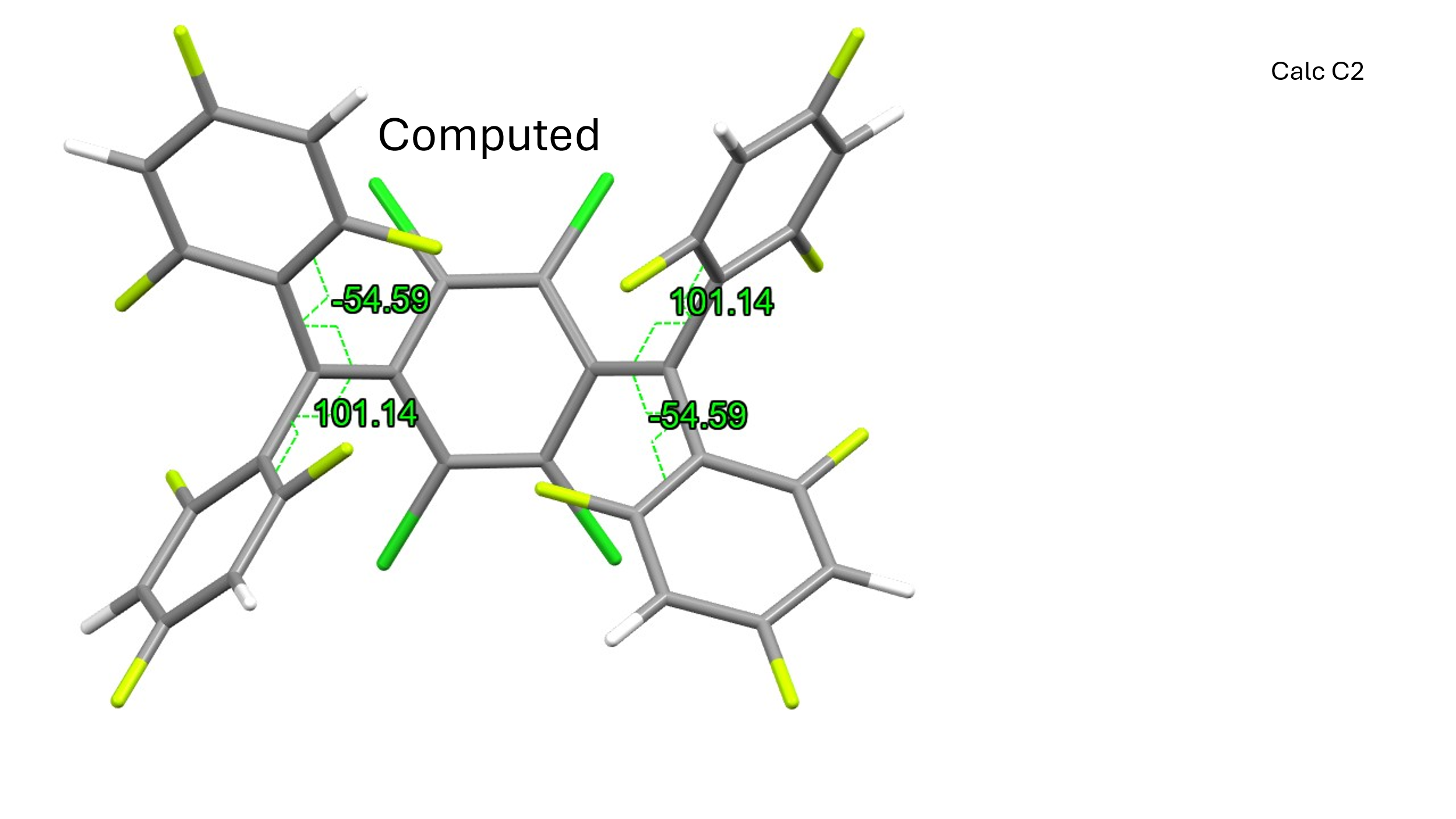

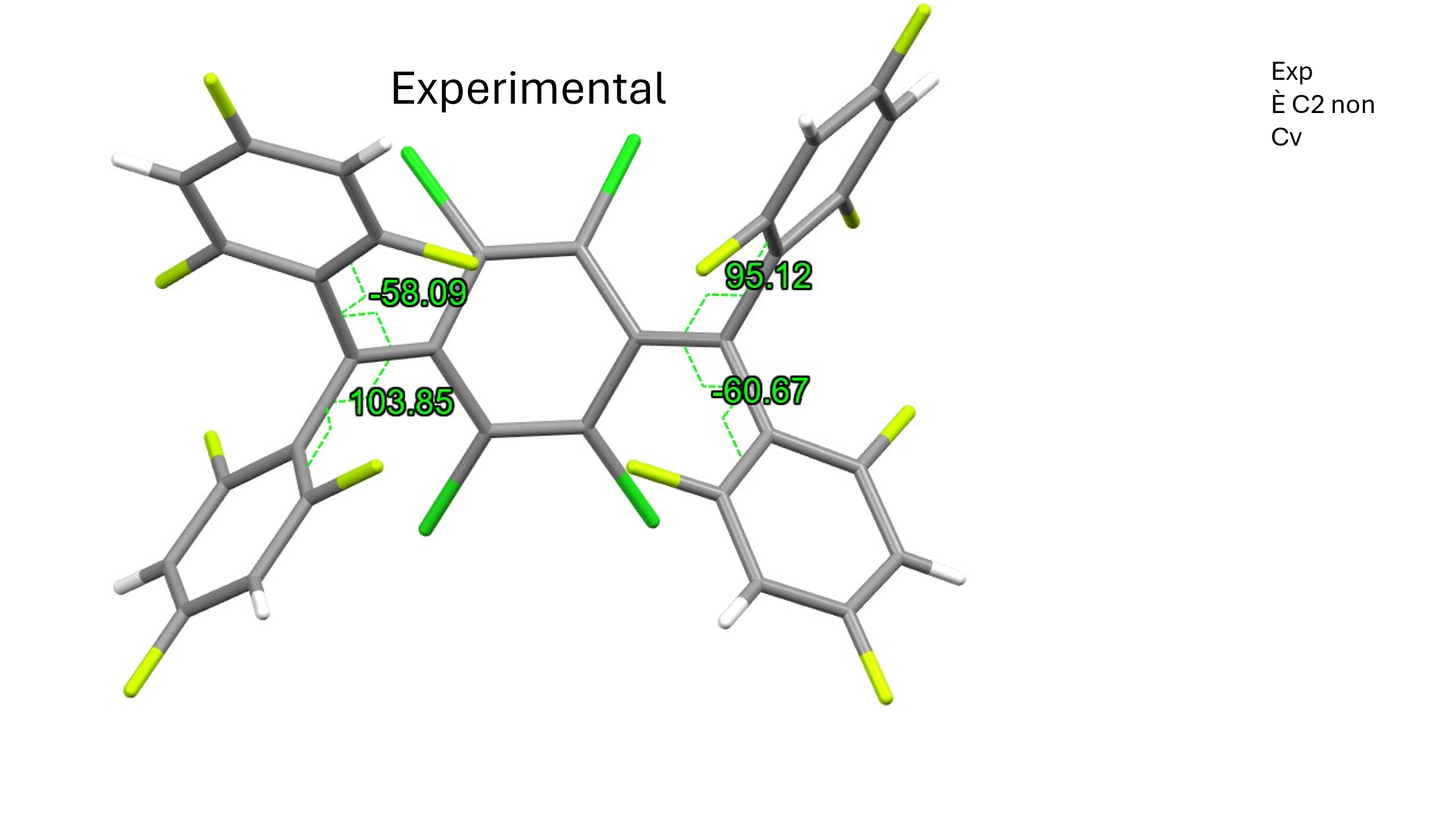


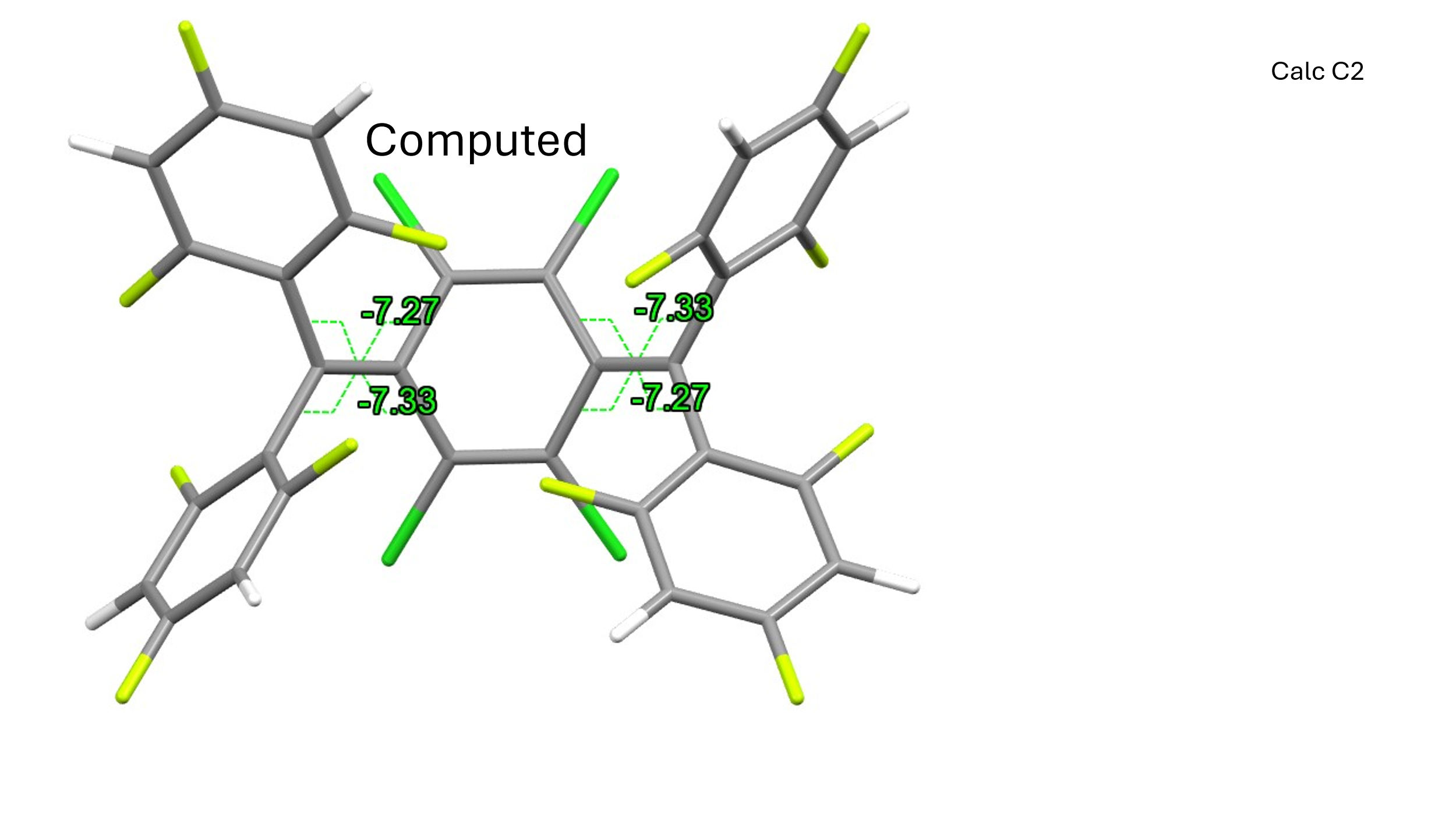

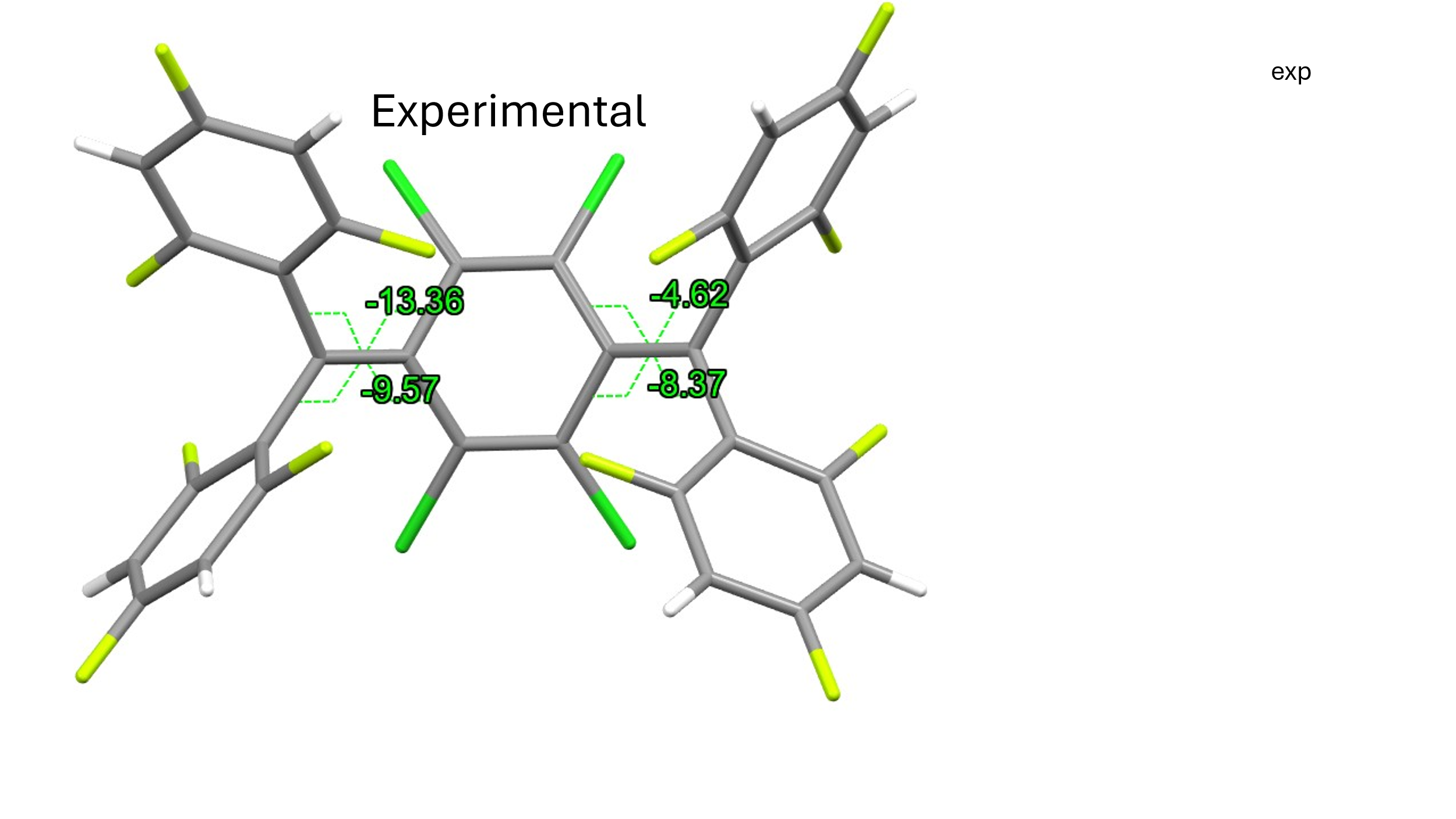


**Figure S47.** Computed bond lengths (Å) of the ground state of the C_2_ symmetry structure of **F,Cl-TTH** at M062X-D3/def2SVP level, side view and comparison with the crystal structure.


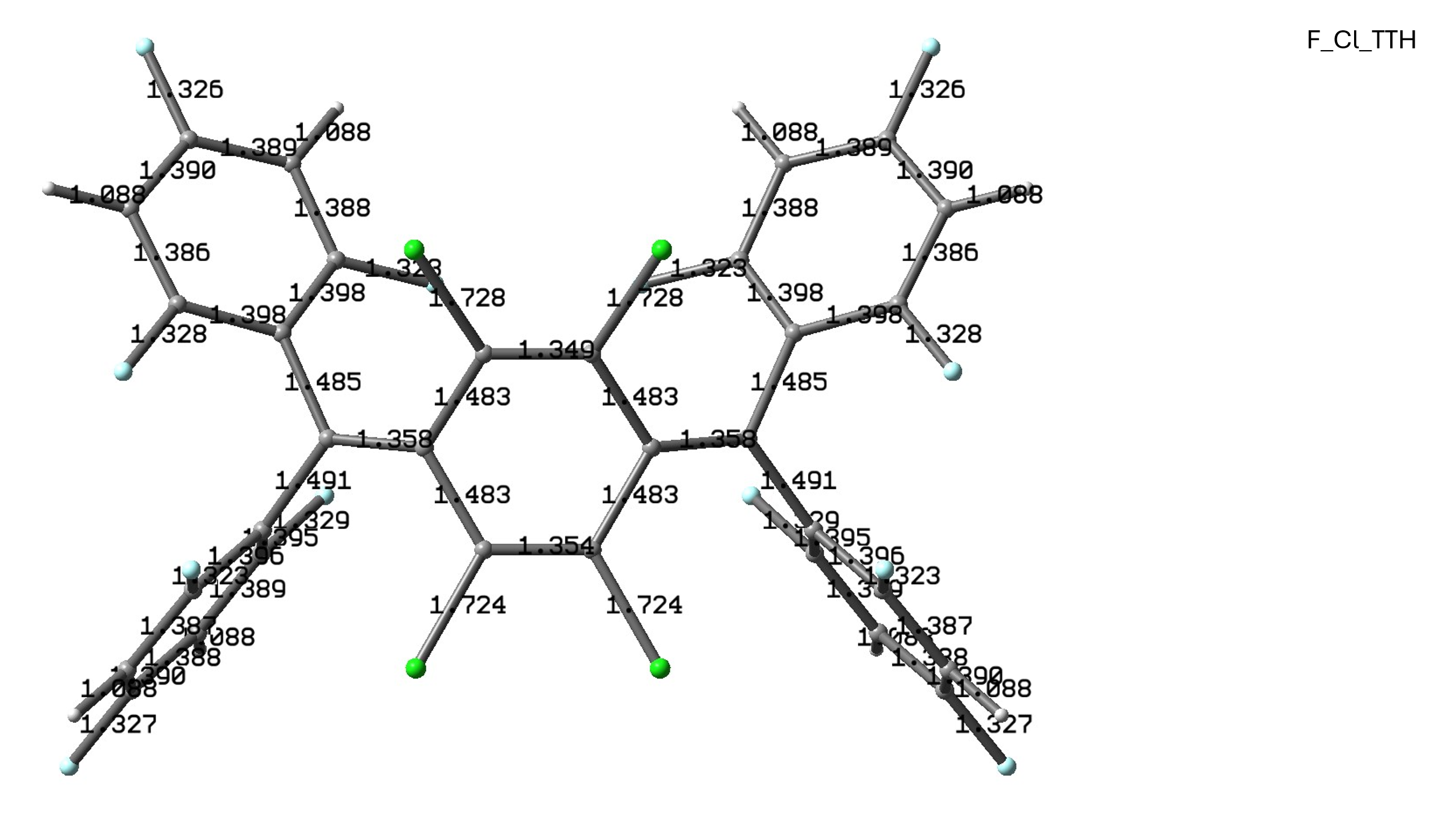

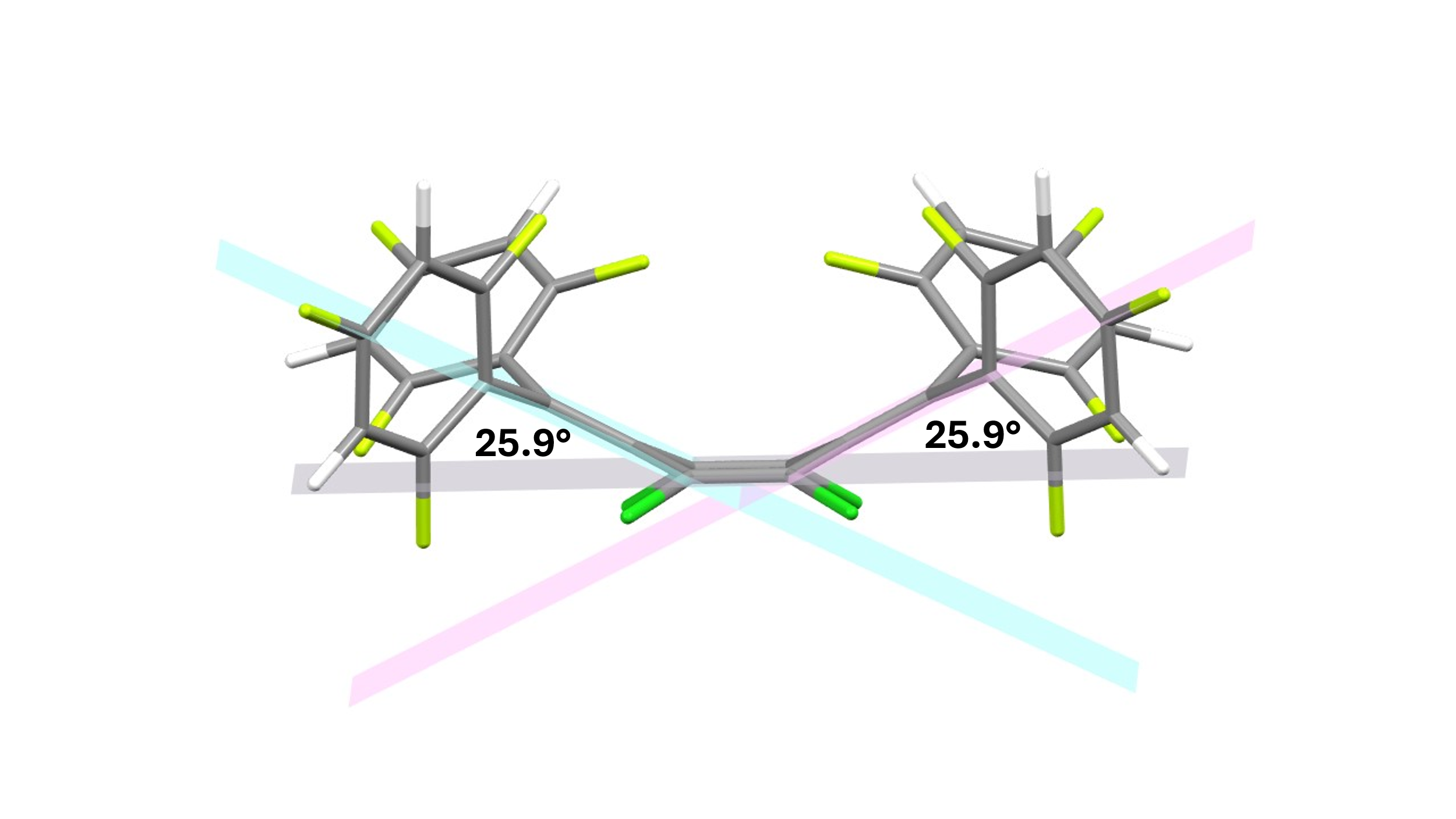


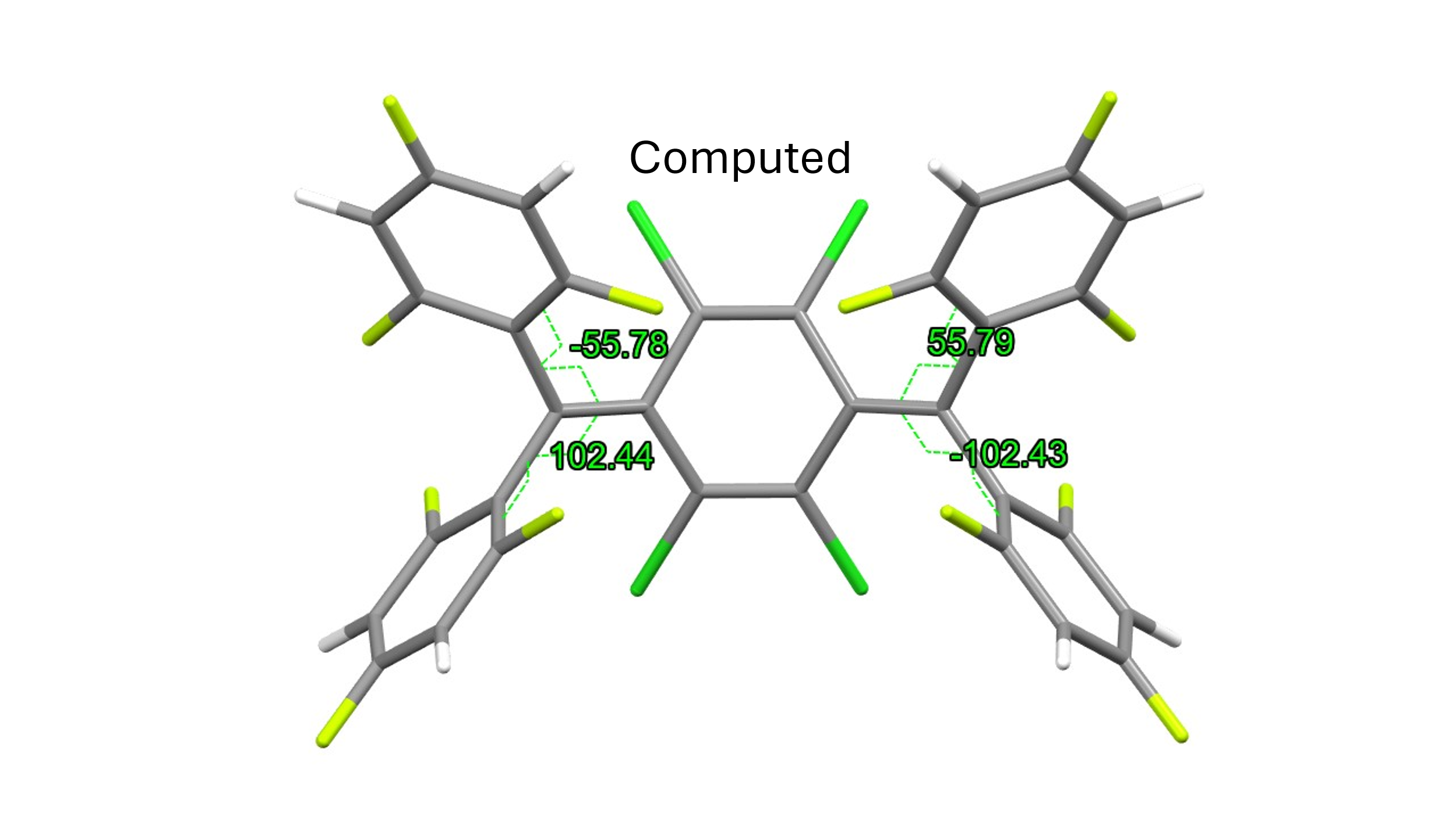

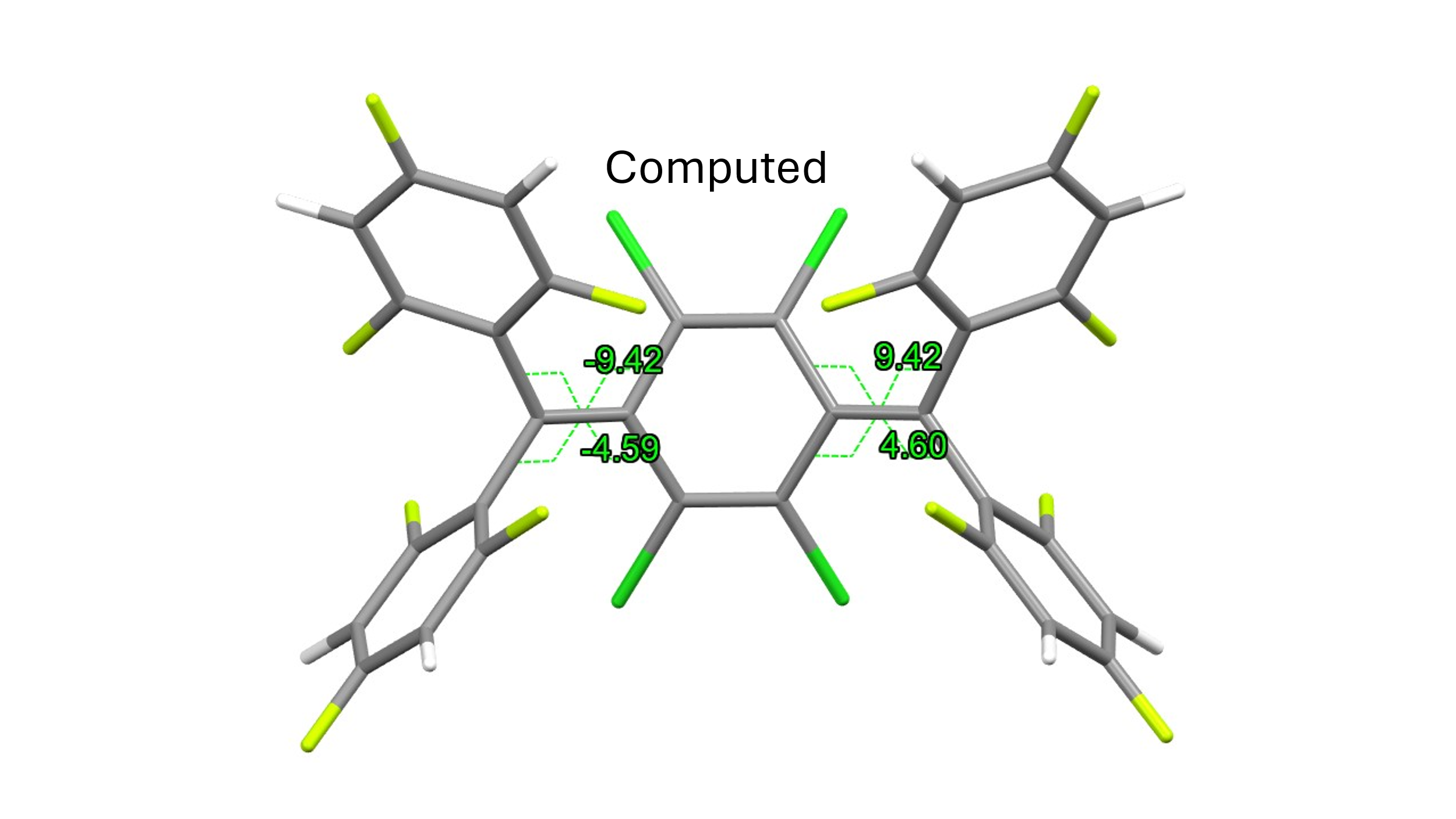


**Figure S48.** Computed bond lengths (Å) of the ground state of the C_s_ symmetry and less stable structure of **F,Cl-TTH** at M062X-D3/def2SVP level, side view and torsional angles.


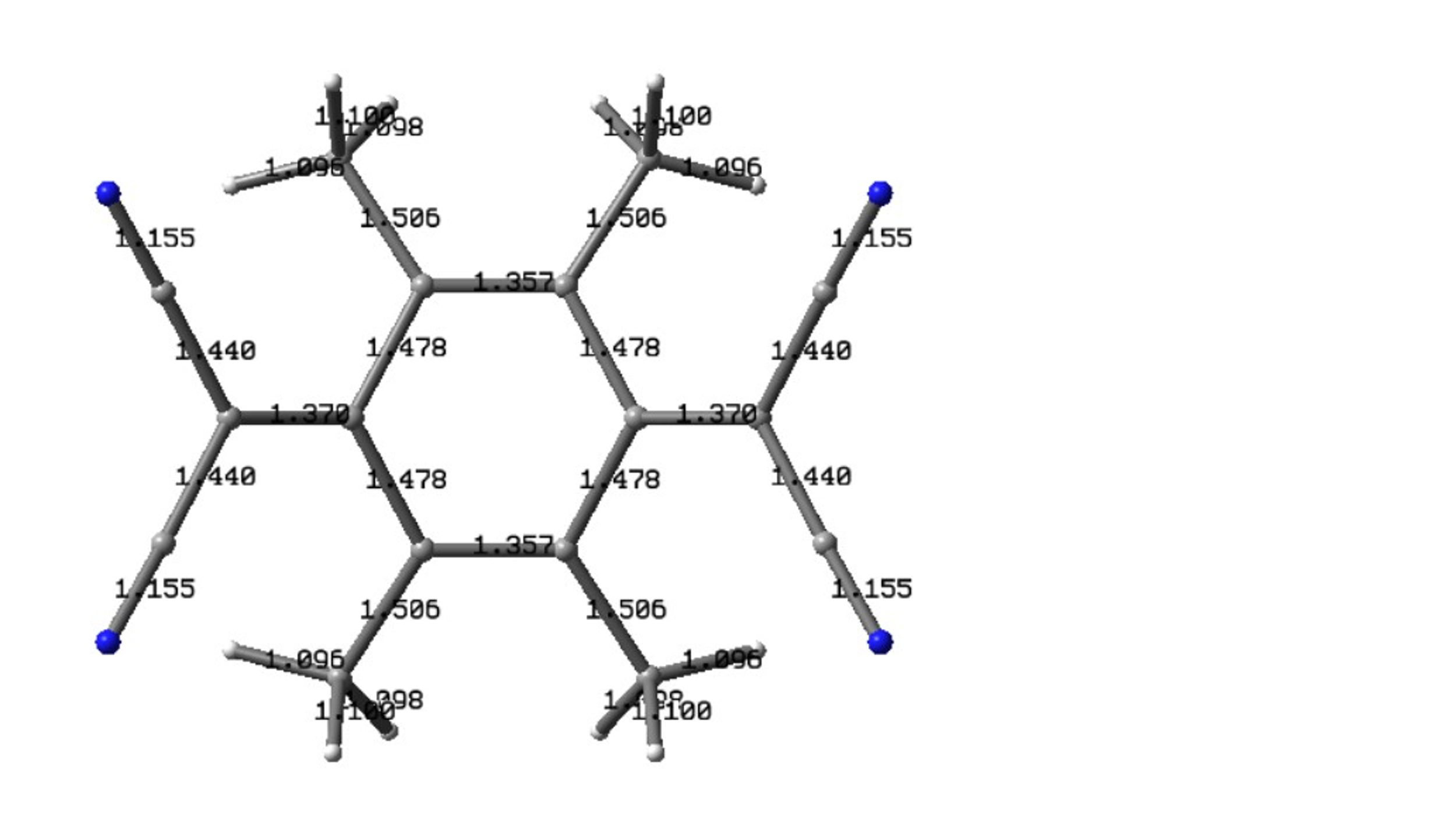

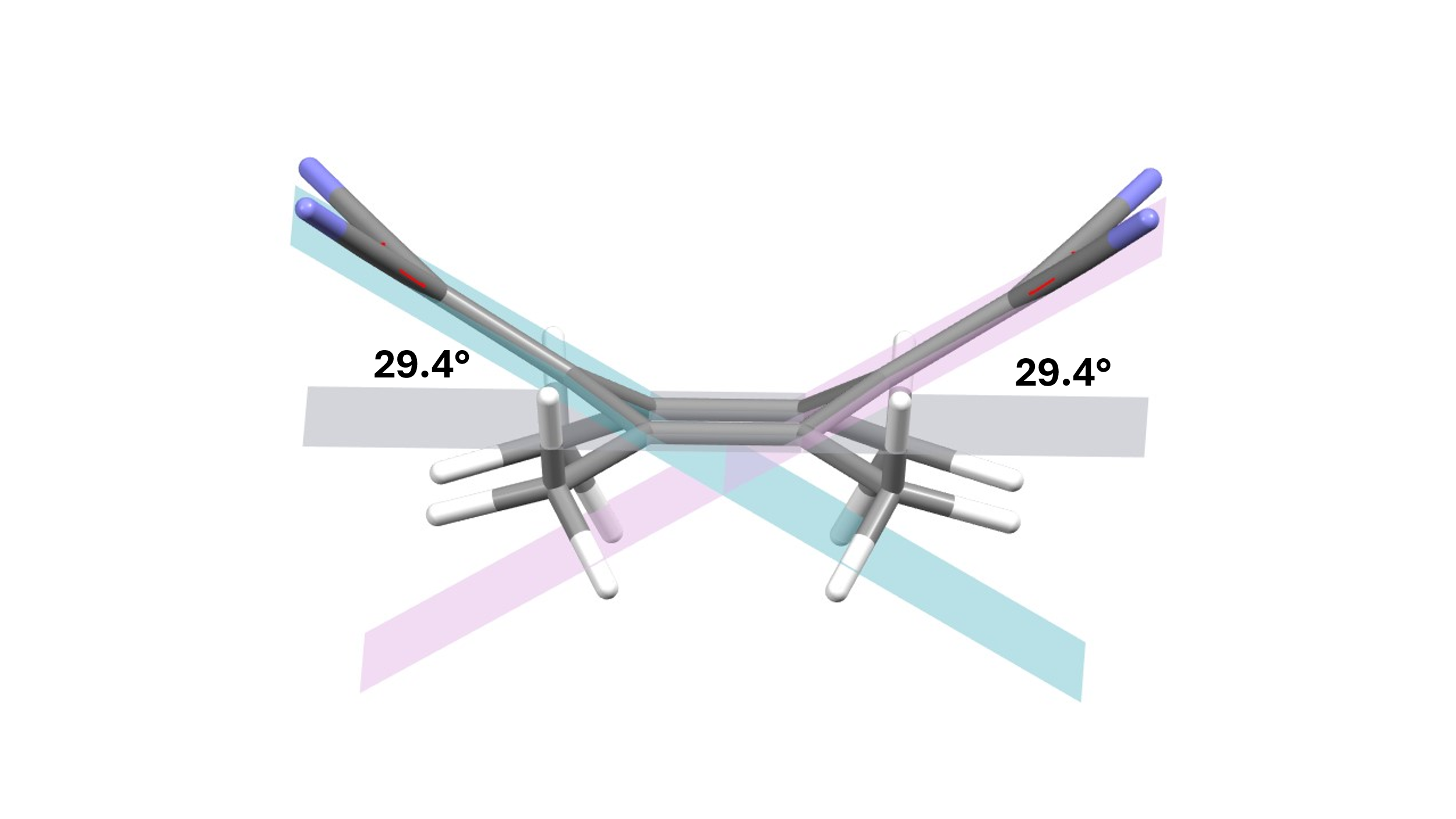


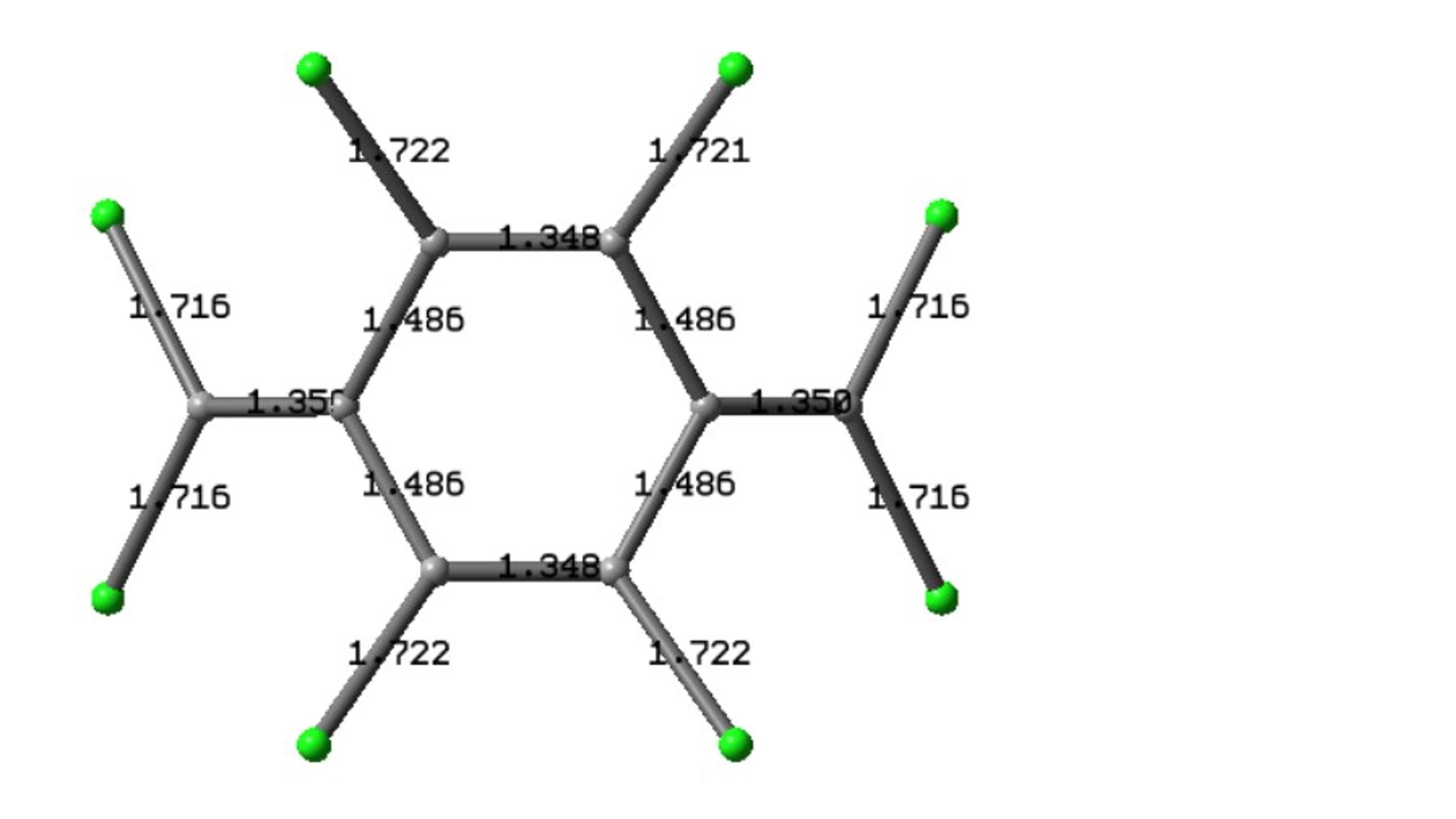

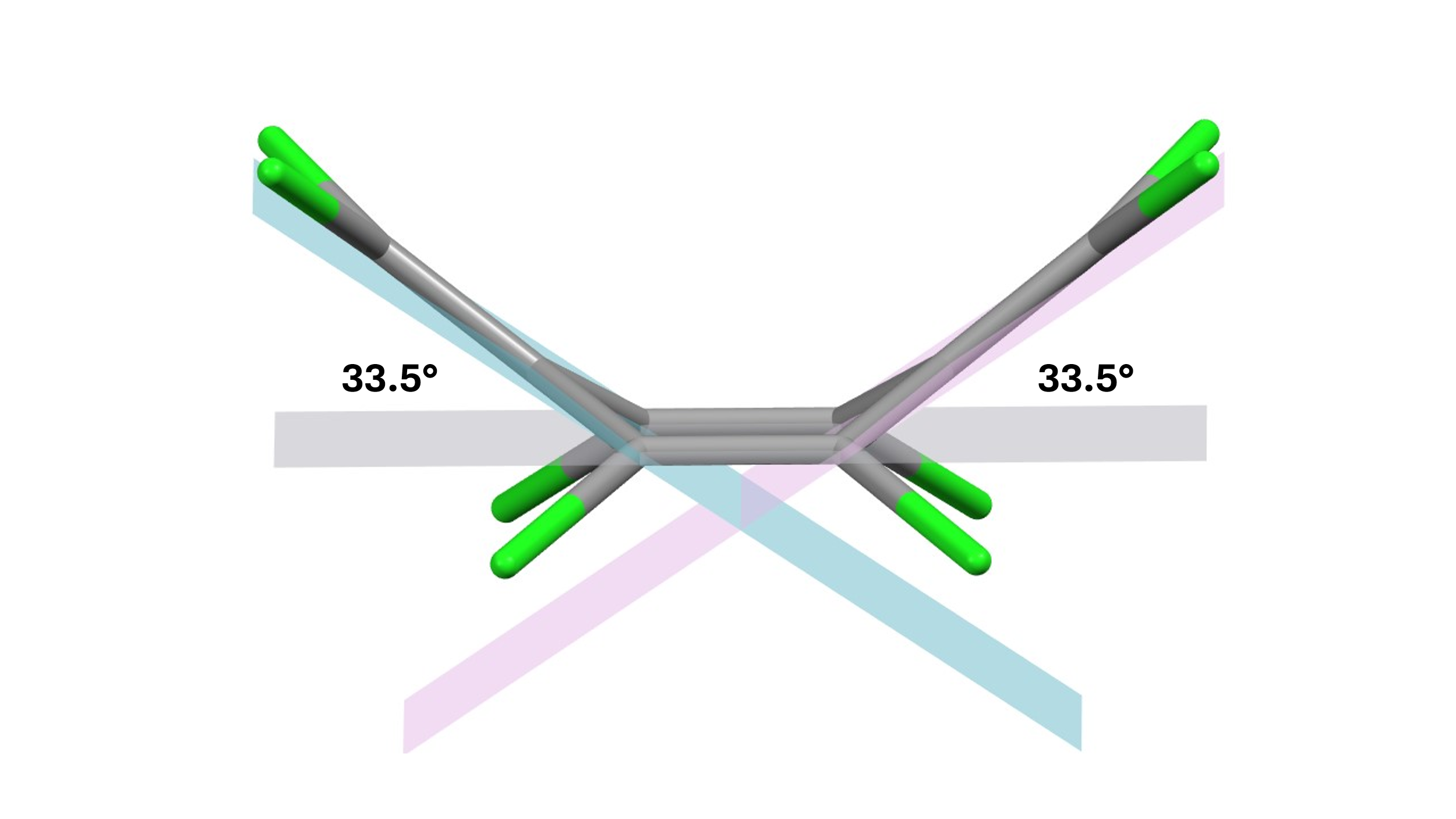


**Figure S49.** Computed bond lengths (Å) of (top) the ground state of **pQDM_1** at M062X-D3/def2SVP level and side view; (bottom) the ground state of **pQDM_2** at M062X-D3/def2SVP level and side view.


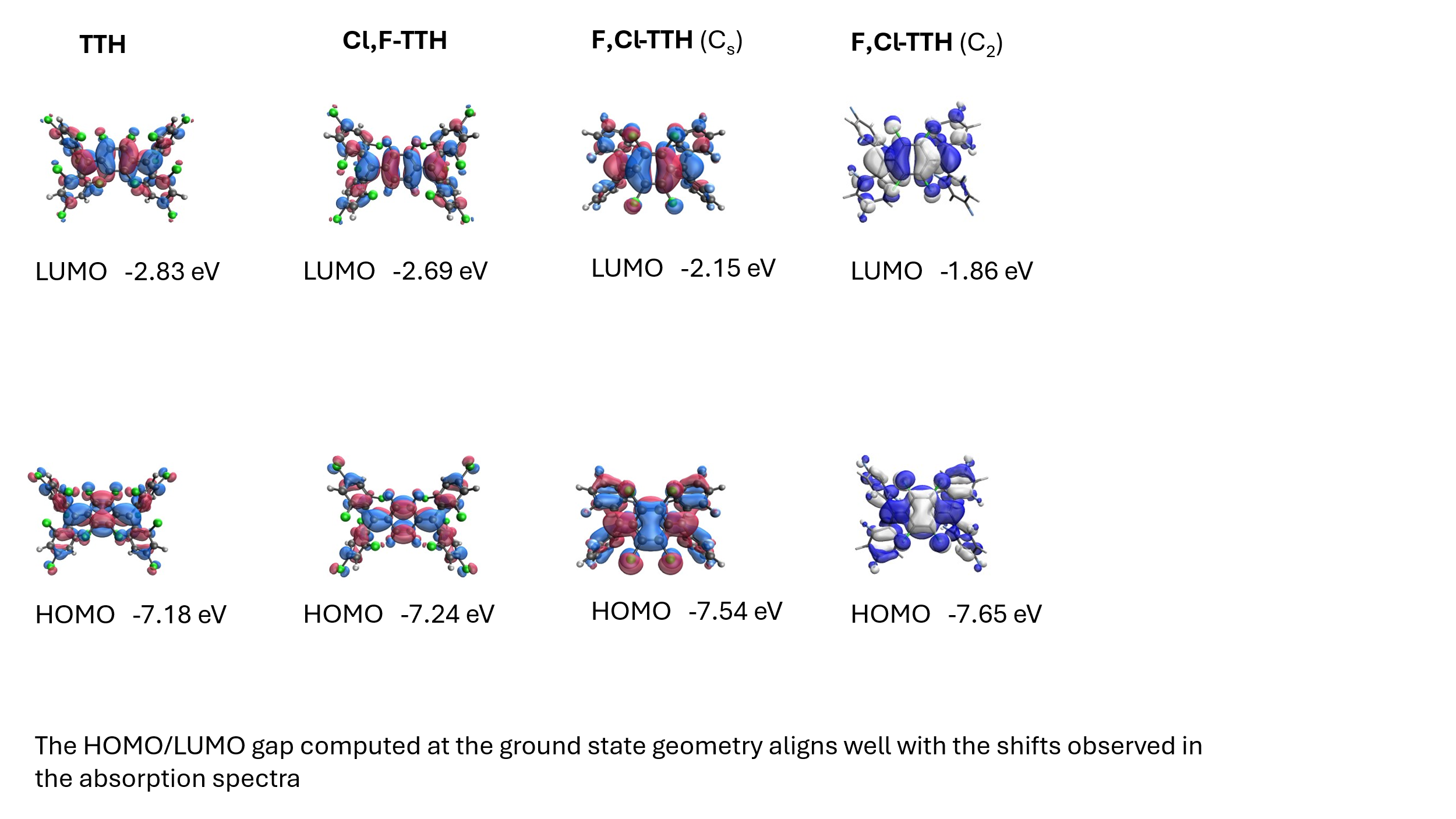


**Figure S50.** Frontier orbitals shapes and energies for **TTH** derivatives computed at M062X-D3/def2SVP level, at their respective ground state geometries.


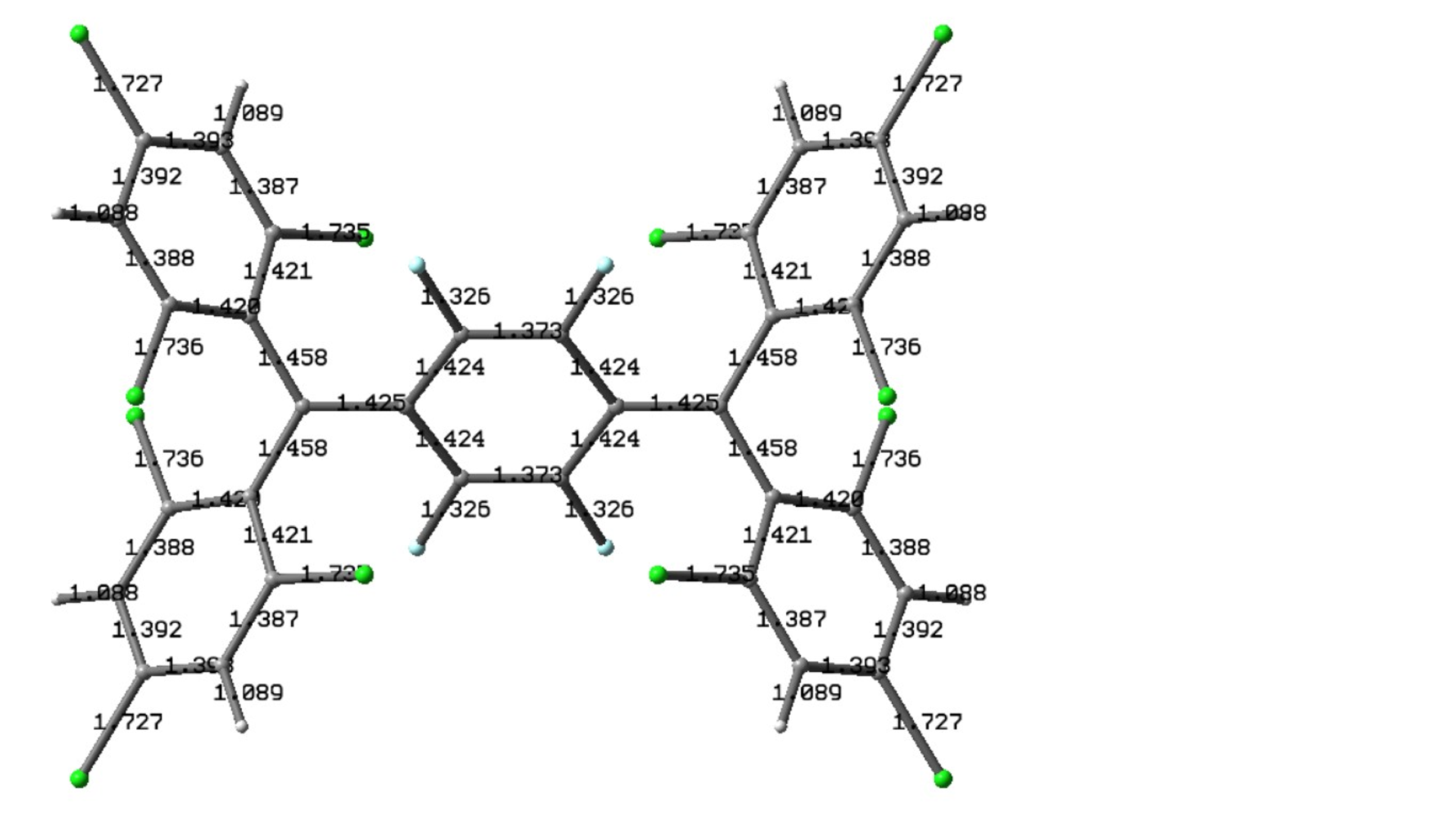

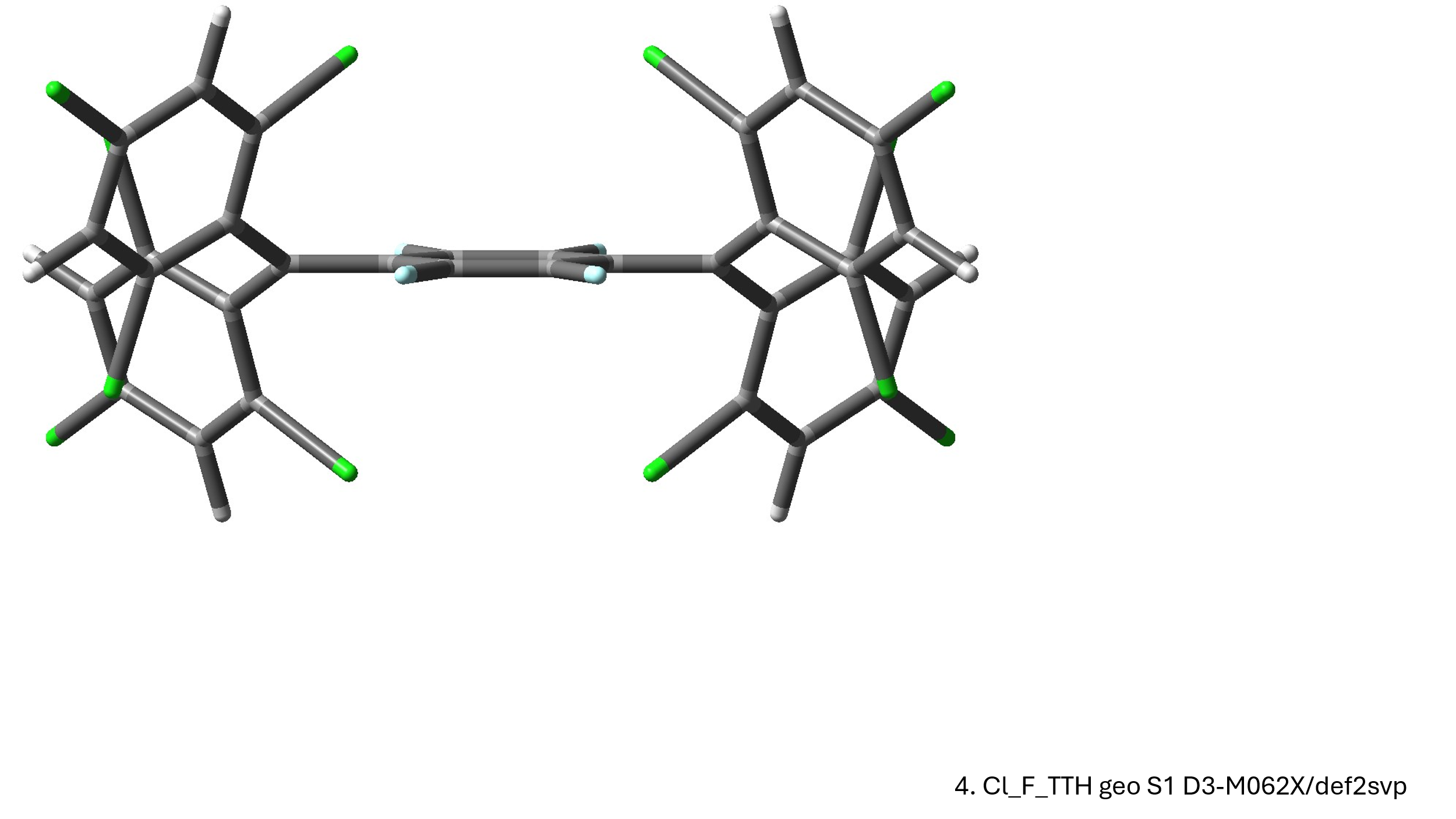

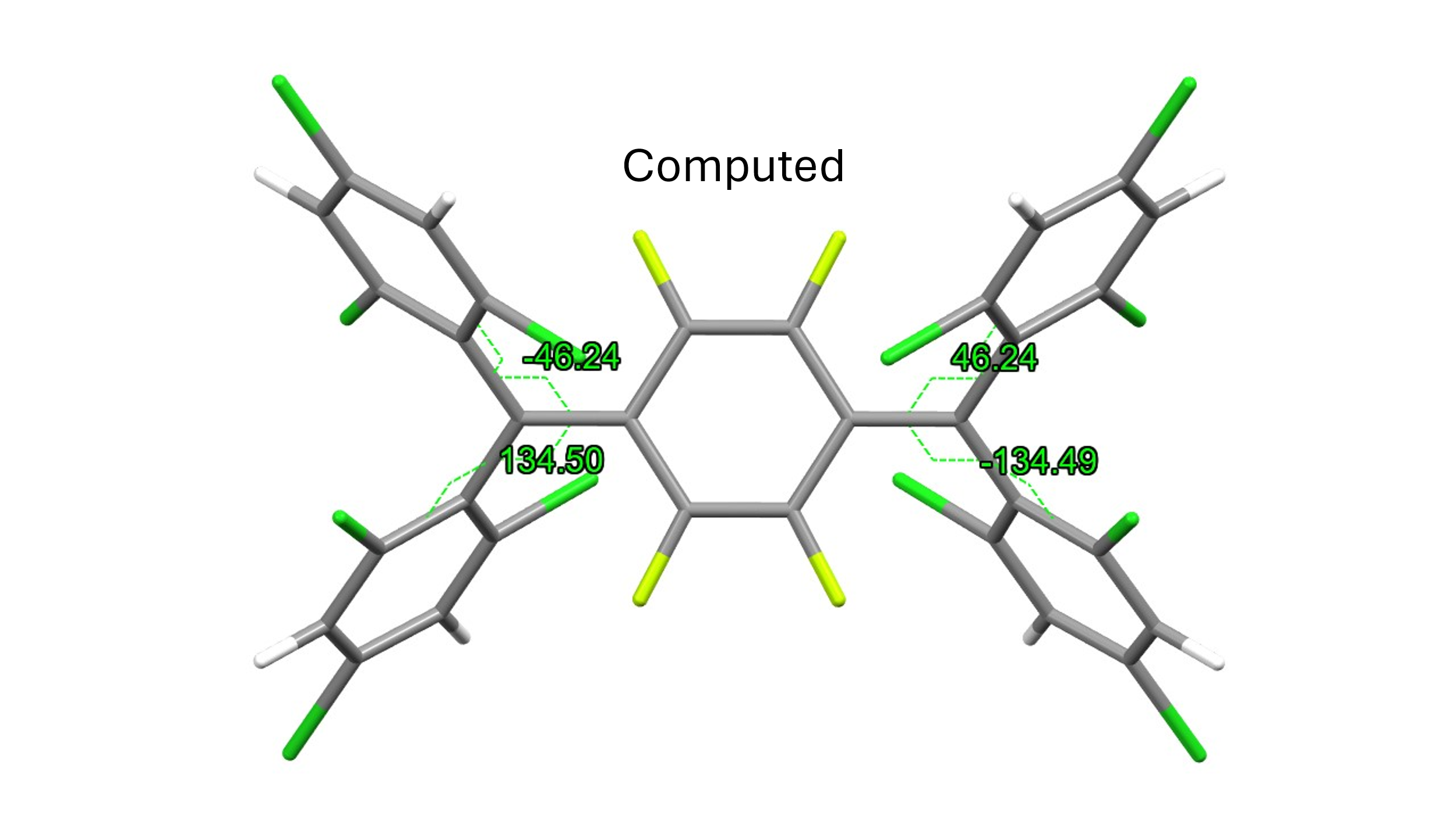

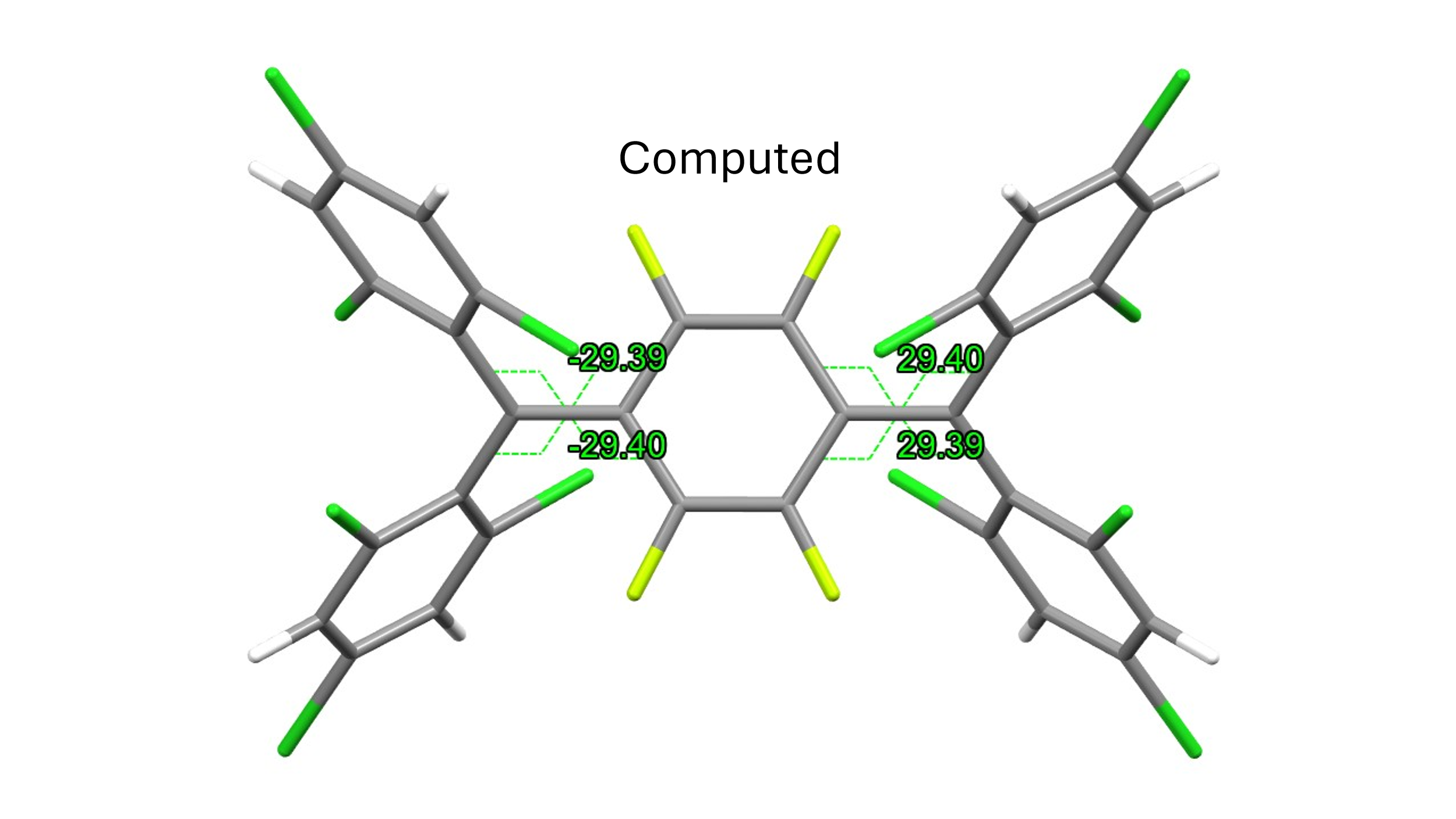


**Figure S51.** Computed bond lengths (Å) of the SE excited state of **Cl,F-TTH** at TD-M062X-D3/def2SVP level, side view and torsional angles.


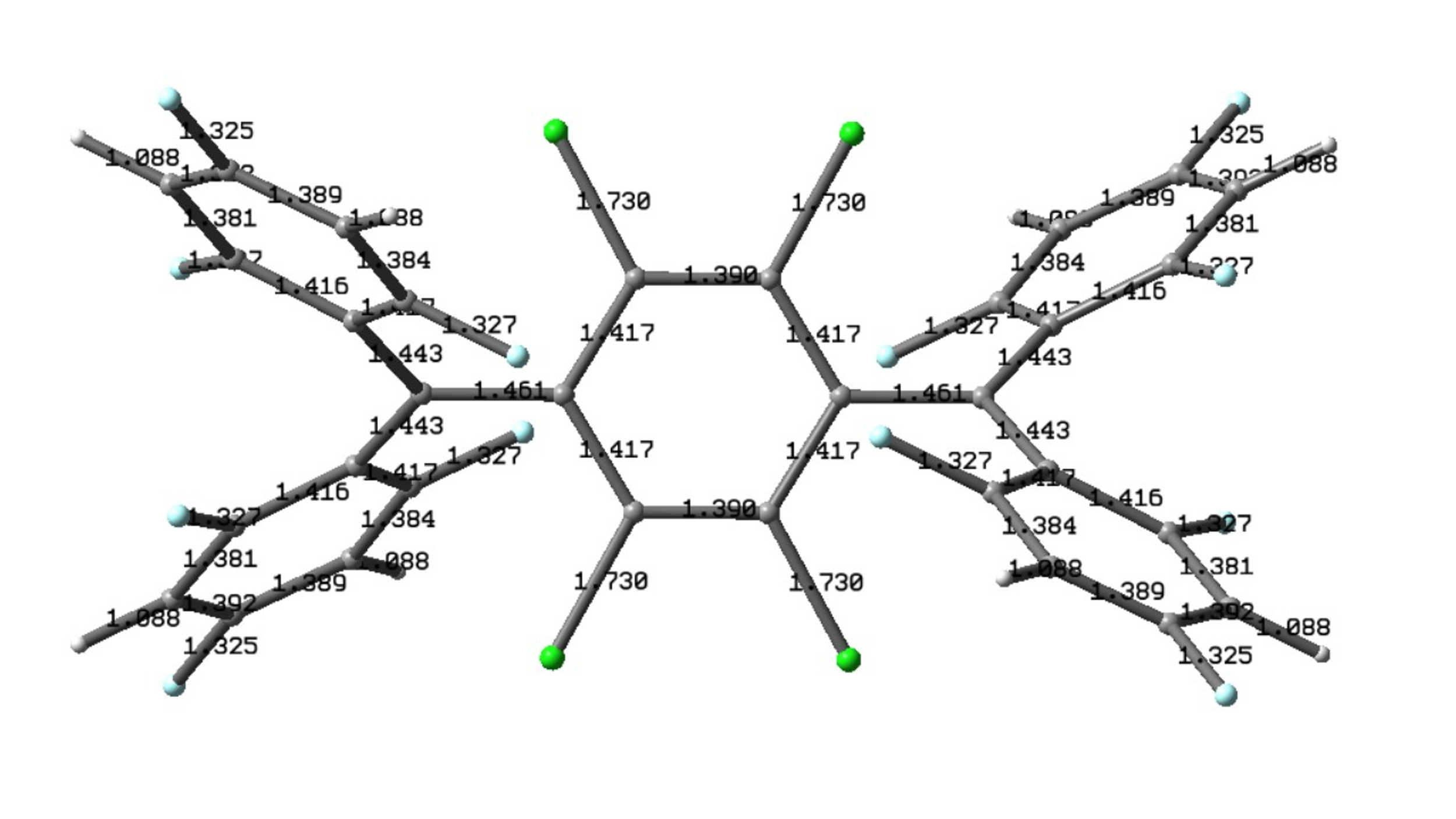

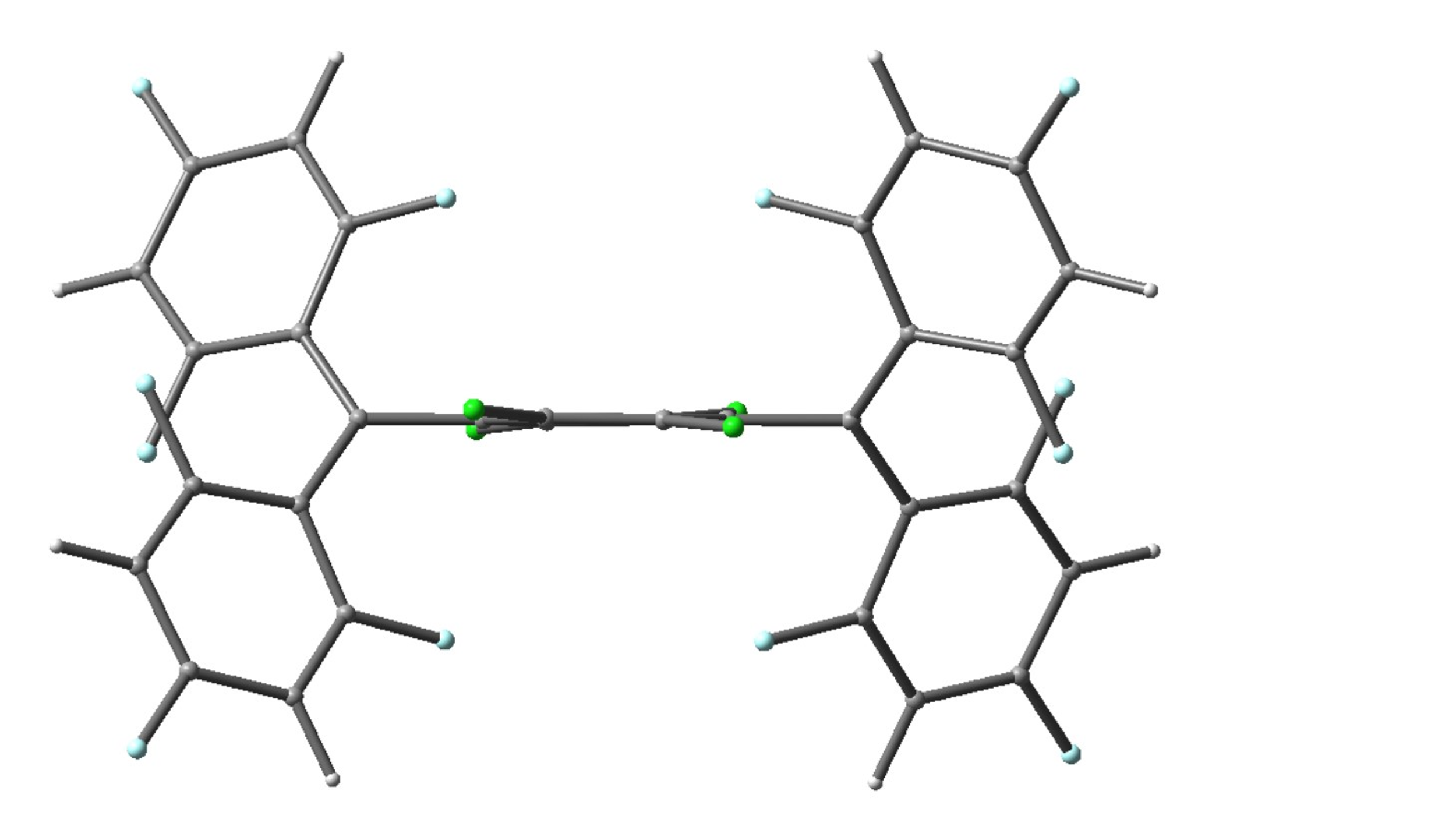


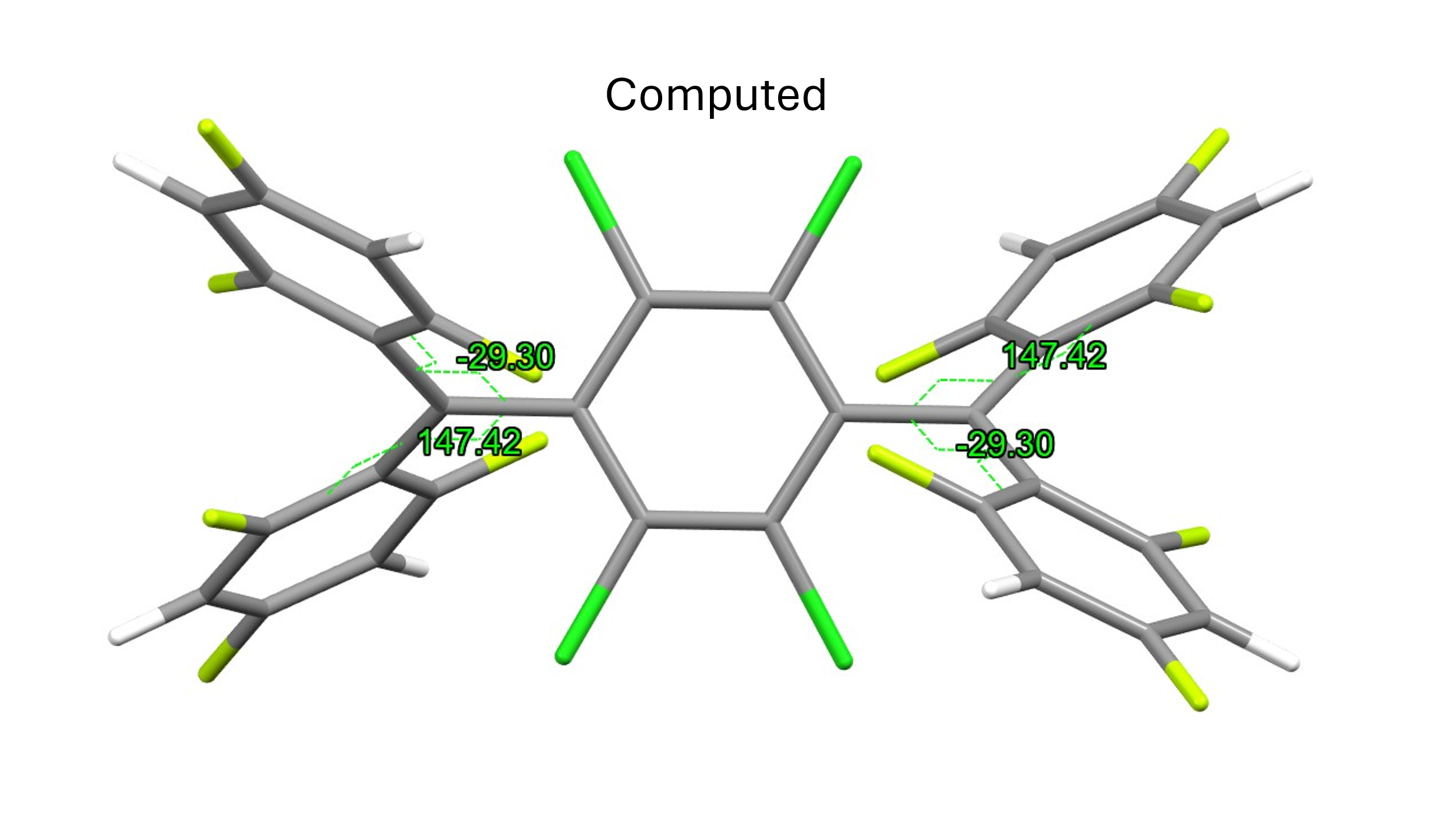


**Figure S52.** Computed bond lengths (Å) of the SE excited state of the C_2_ symmetry point group structure of **F,Cl-TTH** at TD-M062X-D3/def2SVP level, side view and torsional angles.

**Figure S53.** Computed bond lengths (Å) of the SE excited state of the C_s_ symmetry point group structure of **F,Cl-TTH** at TD-M062X-D3/def2SVP level, side view and torsional angles.

**Figure S54.** The simulated absorption spectra of **TTH**, **F,Cl-TTH** (both C_2_ and C_s_ structures) and **Cl,F-TTH**. From vertical excitations computed at TD-M062X-D3/def2SVP level. A Lorentzian linewidth of 0.2 eV was superimposed to each computed transition to facilitate comparison with experimental spectra. The overestimate of 0.2-0.3 eV is typical of the level of theory.

**Figure S55.** Computed bond lengths (Å) of the ground state of the planar (saddle-point) structure of **F,Cl-TTH** at M062X-D3/def2SVP level.

**Figure S56.** Schematic representation of the fragments (**Frag #1, #2 and #3**) used to partition the computed ground state charge distribution in **F,Cl-TTH** and **Cl,F-TTH**.

**Table S15.** Computed charge distributions partitioned according to the fragment definition in Figure S56. The quadrupolar character in both ground and excited states is shown in the last column. For **F,Cl-TTH**, the charge distribution is reported for both the C_2_ minimum and the planar saddle point.

| Molecule / electronic state | Fragment 1 | Fragment 2 | Fragment 3 | Quadrupolar character |
| --- | --- | --- | --- | --- |
| **F,Cl-TTH (C_2_) / S_0_** | -0.029 | 0.058 | -0.029 | ADA |
| **F,Cl-TTH (C_2_) / S_1_** | 0.070 | -0.140 | 0.070 | DAD |
| **F,Cl-TTH (Saddle) / S_0_** | -0.024 | 0.048 | -0.024 | ADA |
| **Cl,F-TTH / S_0_** | 0.029 | -0.058 | 0.029 | DAD |
| **Cl,F-TTH / S_1_** | 0.062 | -0.124 | 0.062 | DAD |

# References

(1) W. Kabsch, *Acta Crystallogr. D Struct. Biol.* **2010**, *66* (2), 125-132.

(2) C. Giacovazzo, Phasing in Crystallography: A Modern Perspective. **2013**, International Union of Crystallography/Oxford University Press.

(3) M. C. Burla, R. Caliandro, B. Carrozzini, G. L. Cascarano, C. Cuocci, C. Giacovazzo, M. Mallamo, A. Mazzone, G. Polidori, *J. Appl. Crystallogr*. **2015**, *48*, 306-309.

(4) G. M. Sheldrick, *Acta Crystallogr. Sect. C Struct. Chem*. **2015**, *71* (1), 3-8.

(5) L. J. Farrugia, *J. Appl. Crystallogr.* **2012**, *45*, 849-854.

(6) S. P. Westrip, *J. Appl. Crystallogr*. **2010**, *43*, 920-925.

(7) C. F. Macrae, I. Sovago, S. J. Cottrell, P. T. Galek, P. McCabe, E. Pidcock, M. Platings, G. P. Shields, J. S. Stevens, M. Towler, *J. Appl. Crystallogr*. **2020**, *53* (1), 226-235.

(8) I. J. Bruno, J. C. Cole, M. Kessler, J. Luo, W. D. S. Motherwell, L. H. Purkis, B. R. Smith, R. Taylor, R. I. Cooper, S. E. Harris, A. G. Orpen, *J. Chem. Inf. Comput. Sci*. **2004**, *44* (6), 2133-2144.

(9) <https://checkcif.iucr.org> (IUCr [checkCIF/PLATON service](http://checkcif.iucr.org/)).

(10) I. H. M. Van Stokkum, D. S. Larsen, R. Van Grondelle, *Biochim Biophys Acta Bioenerg* **2004**, *1657*, 82-104.

(11) J. J. Snellenburg, S. Laptenok, R. Seger, K. M. Mullen, I. H. M. van Stokkum, *J Stat Softw* **2012**, *49*, 1-22.

(12) K. M. Mullen, I. H. M. Van Stokkum, *J Stat Softw* **2007**, *18*, 1-46.

(13) W. R. Dolbier, Jr., P. Xie, L. Zhang, W. Xu, Y. Chang, K. A. Abboud, *J. Org. Chem.* **2008**, *73*, 2469-2472.

(14) C.-H. Liu,. Z. He,. C. Ruchlin, Y. Che, K. Somers, D. F. Perepichka, *J. Am. Chem. Soc.* **2023**, *145*, 15702-15707.

(15) C. R. Groom, I. J. Bruno, M. P. Lightfoot, S. C. Ward, *Acta Crystallogr. B Struct. Sci. Cryst. Eng. Mater*. **2016**, *72* (2), 171-179.

(16) B. Rosenau, C. Krieger, H. A. Staab, *Tetrahedron Lett*. **1985**, *26* (17), 2081-2084.

(17) S. Gali, C. Miravitlles, M. Font-Altaba, *Acta Crystallogr. Sect. B: Struct. Sci. Cryst. Eng. Mater*. **1976**, *32* (17), 3112-3114.

(18) A. Punzi, Y. Dai, C. N. Dibenedetto, E. Mesto, E. Schingaro, T. Ullrich, M. Striccoli, D. M. Guldi, F. Negri, G. M. Farinola, D. Blasi, *J. Am. Chem. Soc*. **2023**, *145* (37), 20229-20241.

(19) R. Wang, T. S. Dols, C. W. Lehmann, U. Englert, *Z. Anorg. Allg*. Chem. **2013**, *639* (11), 1933-1939

(20) B. K. Saha, S. A. Rather, A. Saha, *Cryst.Growth & Des*. **2016**, *16* (6), 3059-3062.

(21) E. A. Meyer, R. K. Castellano, F. Diederich, *Angew. Chem. Int. Ed*. **2003**, *42* (11), 1210-1250.

(22) J.-L. Wang, J.-S. Xu, D. Y. Wang, H. Wang, Z. T. Li, D.-W. Zhang, *CrystEngComm*. **2014**, *16*, 2078-2084.

(23) S. Grimme, J. Antony, S. Ehrlich, H. Krieg, *J. Chem. Phys.* **2010**, *132*, 154104.

(24) S. Grimme, S. Ehrlich, L. Goerigk, *J. Comput. Chem.* **2011**, *32*, 1456-1465.

(25) B. O. Roos, P. R. Taylor, P. E. M. Siegbahn, *Chem. Phys.* **1980**, *48*, 157-173.

(26) C. Angeli, R. Cimiraglia, S. Evangelisti, T. Leininger, J. P. Malrieu, *J. Chem. Phys.* **2001**, *114*, 10252.

(27) C. Angeli, R. Cimiraglia, J. P. Malrieu, *J. Chem. Phys.* **2002**, *117*, 9138-9153.

(28) F. Neese, *Wiley Interdiscip. Rev. Comput. Mol. Sci.* **2012**, *2*, 73-78.

(29) F. Neese, F. Wennmohs, U. Becker, C. Riplinger, *J. Chem. Phys.* **2020**, *152*, 224108.

(30) A. Schäfer, H. Horn, R. Ahlrichs, *J. Chem. Phys.* **1998**, *97*, 2571-2577.

(31) F. Weigend, *J. Comput. Chem.* **2008**, *29*, 167-175.

(32) S. Canola, J. Casado, F. Negri, *Phys. Chem. Chem. Phys.* **2018**, *20*, 24227-24238.

(33) K. Yamaguchi, *Chem. Phys. Lett.* **1975**, *33*, 330-335.

(34) M. J. Frisch, G. W. Trucks, H. B. Schlegel, G. E. Scuseria, M. A. Robb, J. R. Cheeseman, G. Scalmani, V. Barone, G. A. Petersson, H. Nakatsuji, X. Li, M. Caricato, A. V. Marenich, J. Bloino, B. G. Janesko, R. Gomperts, B. Mennucci, H. P. Hratchian, J. V. Ortiz, A. F. Izmaylov, J. L. Sonnenberg, D. Williams-Young, F. Ding, F. Lipparini, F. Egidi, J. Goings, B. Peng, A. Petrone, T. Henderson, D. Ranasinghe, J. Zakrzewski, V. G.; Gao, N. Rega, G. Zheng, W. Liang, M. Hada, M. Ehara, K. Toyota, R. Fukuda, J. Hasegawa, M. Ishida, T. Nakajima, Y. Honda, O. Kitao, H. Nakai, T. Vreven, K. Throssell, J. Montgomery, J. A., J. E. Peralta, F. Ogliaro, M. J. Bearpark, J. J. Heyd, E. N. Brothers, K. N. Kudin, V. N. Staroverov, T. A. Keith, R. Kobayashi, J. Normand, K. Raghavachari, A. P. Rendell, J. C. Burant, S. S. Iyengar, J. Tomasi, M. Cossi, J. M. Millam, M. Klene, C. Adamo, R. Cammi, J. W. Ochterski, R. L. Martin, K. Morokuma, O. Farkas, J. B. Foresman, D. J. Fox, *Revis. A.03, Gaussian, Inc., Wallingford CT,* **2016**.
